# Elucidating the Curtin–Hammett Principle in Glycosylation Reactions: The Decisive Role of Equatorial Glycosyl Triflates

**DOI:** 10.1021/jacs.5c03519

**Published:** 2025-06-23

**Authors:** Peter H. Moons, Frank F. J. de Kleijne, Teun van Wieringen, Floor ter Braak, Hero R. Almizori, Luuk J. H. Jakobs, Willem P. M. de Kleijne, Floris P.J.T. Rutjes, Jonathan Martens, Jos Oomens, Peter A. Korevaar, Paul B. White, Thomas J. Boltje

**Affiliations:** † Synthetic Organic Chemistry, Institute for Molecules and Materials, 6029 Radboud University Nijmegen, Heyendaalseweg 135, 6525 AJ Nijmegen, The Netherlands; ‡ FELIX Laboratory, Institute for Molecules and Materials, Radboud University Nijmegen, Toernooiveld 7, 6525 ED Nijmegen, The Netherlands; § Physical Organic Chemistry, Institute for Molecules and Materials, Radboud University Nijmegen, Heyendaalseweg 135, 6525 AJ Nijmegen, The Netherlands

## Abstract

The glycosylation reaction represents a crucial and challenging reaction used in oligosaccharide synthesis. Specifically, attaining complete stereocontrol during glycosylation reactions remains challenging. Its complex nature is defined by the highly reactive intermediates that form upon the activation of a glycosyl donor. Low-abundant species may afford the major product via Curtin–Hammett kinetics and have long been proposed to play a major role. Therefore, characterizing these elusive stereodirecting intermediates is key to understanding glycosylation reaction mechanisms. Herein, we applied a combination of (exchange) NMR techniques to establish the equilibration rates of glycosyl triflate reaction intermediates and their ensuing glycosylation reaction kinetics. To this end, we studied the glycosylation reactions of 6,3-mannuronic acid and 6,3-glucuronic acid lactone donors. Using the complete set of reaction kinetics data, we constructed a computational kinetic model that shows that these compounds indeed react according to a Curtin–Hammett scenario. Furthermore, we were able to rationalize the observed stereochemical reaction outcomes using quantum-chemically computed potential energy surfaces for these glycosylation reactions. Hence, this workflow can now be used to obtain a complete reaction kinetics overview to retrieve the reaction pathway(s) that drive product formation.

## Graphical abstract

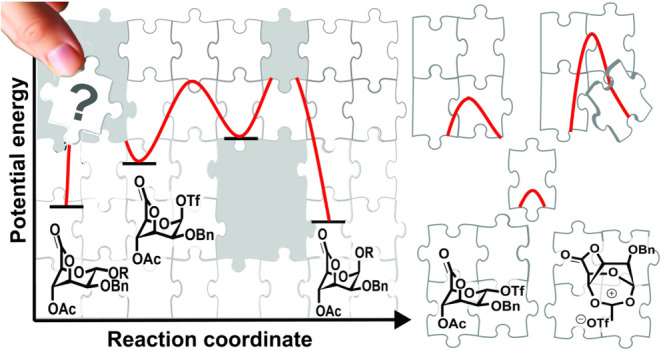

## Introduction

The glycosylation reaction is central to complex carbohydrate synthesis, yet attaining complete stereocontrol remains challenging. During a glycosylation reaction, a glycosyl donor is activated to form electrophilic reaction intermediates that react with a nucleophilic hydroxyl group on the glycosyl acceptor to form a glycoside product. Nucleophilic addition can occur from the α-face or β-face of the reactive intermediate, thereby forming diastereoisomeric α- or β-glycosides, respectively. Most promoter systems afford the *quasi*-stable axial glycosyl triflate as the main observable reaction intermediate after activation of the glycosyl donor at low temperature.^1,2^ Three generalized reaction pathways are often proposed that can explain the formation of the α- or β-glycoside product from this central intermediate (Figure 1).^3,4^

**Figure 1:**
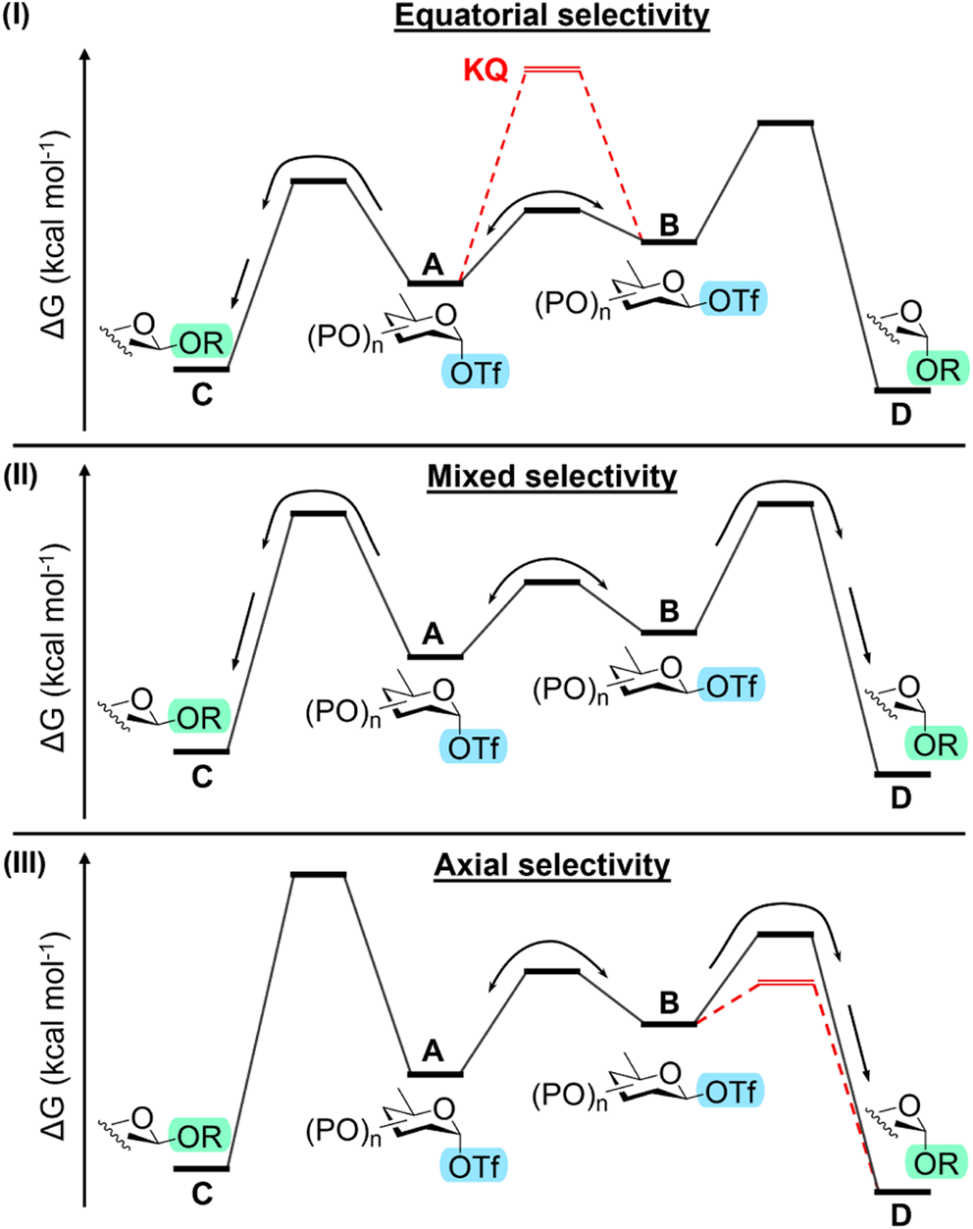
Illustrations of scenarios **I**–**III** with the axial triflate as the major species and equatorial triflate as the minor species. In scenario **I**, product formation originates from the main reaction intermediate observed. The red (dotted) line represents a kinetic quenching (KQ) scenario. In scenario **II**, product formation derives from both axial and equatorial glycosyl triflates with poor selectivity. In scenario **III**, the red line represents a Curtin–Hammett-like scenario, where the ratio A/B changes as the reaction progresses. The interconversion rate is faster than the equatorial product-forming step, but it is similar (*k*_AB_,*k*_BA_ ≈ *k*_BD_ | *k*_BD_ ≫ *k*_AC_) or slower (*k*_BD_ > *k*_AB_,*k*_BA_ > *k*_AC_) than the axial product-forming step, hence still providing axial selectivity. The black line represents a classic Curtin–Hammett scenario where almost exclusively D is formed via B, while the ratio of A/B remains constant over the course of the reaction.

In scenario **I**, the axial glycosyl triflate (A) acts as the reactive intermediate and is displaced by the nucleophile in an S_N_2-like manner to afford an equatorial glycoside (C) as the major product (Figure 1, I). Reaction mechanisms of this type have been most extensively studied in the context of the Crich β-mannosylation and, although they proceed with considerable dissociative character, displacement of the axial glycosyl triflate by the nucleophilic glycosyl acceptor is diastereospecific.^5–8^

The second scenario (**II**) represents a reaction where the axial glycosyl triflate (A) is observed as the main reaction intermediate, yet the formation of a mixture of glycoside products (C and D) is observed (Figure 1, II). In this case, glycosylation reactions are proposed to take place via a fast equilibration of the axial glycosyl triflate with the less stable, but more reactive, equatorial glycosyl triflate (B). Both glycosyl triflate species are product-forming reaction intermediates as the equilibration rate is faster than that of product formation (*k*_AB_,*k*_BA_ ≫ *k*_AC_,*k*_BD_).^3^ In this case, the stereochemical outcome of the reaction depends on the difference in the product-forming rates. If the difference in the rate of the product-forming steps is small, a mixture of products is obtained.

The third scenario (**III**) represents a situation in which the axial glycosyl triflate (A) is observed as the main reaction intermediate, yet it provides an axial glycoside product (D) with very high diastereoselectivity (Figure 1, III). Glycosylation reactions of this type are proposed to occur *via* fast equilibration of the axial glycosyl triflate with the equatorial glycosyl triflate, with the latter being the sole product-forming intermediate by S_N_2-like displacement. Hence, a “classical” Curtin–Hammett scenario is at play where the minor reactive intermediate produces the majority of product while maintaining the ratio of reaction intermediates by rapid equilibration. This reaction pathway has been proposed by Lemieux and co-workers in the context of glycosyl halide donors and can, in principle, be extended to other reactive intermediates such as glycosyl triflates.^9,10^ Herein, the stereochemical outcome of the reaction is the result of a large difference in the product-forming rates (*k*_BD_ > *k*_AC_) and an even faster rate of intermediate interconversion (*k*_AB_,*k*_BA_ ≫ *k*_AC_,*k*_BD_).^3,4^ Falling within the same kinetic scenario, **III** is a Curtin–Hammett-like scenario where the formation of D from B is faster than equilibration of A to B (Figure 1, III, red line). While this is not under Curtin–Hammett control according to its definition, it still results in the selective formation of D *via* the high-energy reaction intermediate B.

Considering these three mechanistic scenarios, we can understand that the overall stereochemical outcome of glycosylation reactions is determined by an intricate interplay of the equilibration and product-forming rates. This may explain why the stereochemical outcome of glycosylation reactions is so sensitive to parameters such as glycosyl donor choice,^11,12^ acceptor reactivity,^13,14^ stereoelectronic effects,^15–17^ solvent effects,^18,19^ concentration,^20,21^ and promoter choice.^22^

Gaining a detailed mechanistic understanding of the glycosylation reaction has been a long-standing challenge. Scenario **I** represents the most straightforward kinetic model and is well studied and understood.^23^ In contrast, mechanistic studies investigating scenario **II** and **III** are much more challenging as they require the detection of the extremely short-lived reactive species responsible for product formation (*e.g.*, equatorial glycosyl triflates), as well as a full itinerary of equilibration and product-forming rates. Asensio and co-workers have made an important contribution toward this goal by demonstrating that scenario **III** is operative in 4,6-benzylidene-protected glucosyl and allosyl donors that contain methyl ethers at C-2 and C-3.^24^ In these cases, the reactive intermediates involved (axial and equatorial glycosyl triflates) were relatively stable and could therefore be characterized using one-dimensional (1D), two-dimensional nuclear magnetic resonance (2D NMR). In the case of the allosyl donor, glycosyl triflate interconversion could be investigated with ^1^H EXSY NMR as the population of both species was sufficiently high. In contrast, the equatorial triflate population was too low to perform ^1^H EXSY NMR in the gluco-case. Finally, they recorded the reaction kinetics of the glycosylation reaction using variable-temperature spectroscopy (VT-NMR) to assign the kinetic scenario. However, the main limitation of 1D, 2D, and EXSY NMR is that only relatively high-abundance *quasi*-stable species can be investigated. Hence, very low-abundance reaction intermediates (<2%) cannot be studied, thus creating a major obstacle in the investigation of the glycosylation reaction mechanism.

To overcome this hurdle, we applied various forms of exchange NMR^25,26^ to detect “invisible” low-abundance, high-energy reaction intermediates.^27,28^ VT-NMR experiments were performed in which axial glycosyl triflates were generated under relevant conditions in an NMR tube (*e.g.*, −80 °C in CD_2_Cl_2_). More reactive and low-abundance reaction intermediates that equilibrate with the axial glycosyl triflate could be detected using chemical exchange saturation transfer (CEST) NMR. By exploiting the chemical equilibrium, saturation of the high-energy “invisible” species could be transferred to the visible species (axial glycosyl triflate), thereby enabling the indirect observation of highly reactive intermediates. We applied this technique to observe the equilibration of axial glycosyl triflates with high-energy intermediates such as dioxanium ions and equatorial glycosyl triflates.^27–30^ In addition, we were able to measure the rate of triflate dissociation that is associated with reaction intermediate equilibration using exchange spectroscopy (EXSY) NMR. The latter has been an essential tool to understand the mechanism of reaction intermediate equilibration.^24,31,32^ By variation of the mixing time and measurement of the degree of magnetization transfer, exchange kinetics of the equilibrating reaction intermediates can be extracted, thus providing critical information needed to understand scenarios **II** and **III**. However, previous studies did not provide the reaction kinetics of the product-forming step, which would be required to construct a complete kinetic model and hence conclusively assign a kinetic scenario (**I**–**III**). Obtaining such data *via* NMR has been challenging due to the high speed of the glycosylation reactions.

Herein, we report a comprehensive integrated NMR-based workflow and computational model to assign the three distinct kinetic scenarios **I**–**III**. To enable this, we unraveled the structure and exchange kinetics of glycosylation reaction intermediates derived from 6,3-uronic acid lactone glycosyl donors and determined their reaction kinetics with a glycosyl acceptor (Scheme 1). We showed that 6,3-mannuronosyl lactones provided axial (β) glycoside products, while the corresponding glucuronosyl derivative was much less stereoselective. In both cases, a C-4 acyl group was needed to prevent the formation of 1,4-anhydro sugar byproduct.^33,34^ By employing the newly developed workflow, we were able to retrieve the reasons for the contrasting glycosylation behavior of glucuronosyl and mannuronosyl lactones. To this end, we used (exchange) NMR to detect equatorial glycuronosyl triflates and measure their exchange rates and glycosylation reaction kinetics. This data was used to construct a kinetic computer model describing the kinetic scenario by fitting the intermediate interconversion and product-forming rates with high accuracy. Lastly, we calculated the transition state (TS) energies of these steps to further increase the confidence in the assignment of the kinetic scenario. These calculations reproduced the relative barrier heights for reaction intermediate interconversion and product formation. From these experiments, we conclude that glycosylations with the glucuronosyl derivative and a strong nucleophile proceeded *via* the axial glycosyl triflate intermediate (scenario **I**). In case a weaker nucleophile was used, both axial and equatorial glycosyl triflates are reactive intermediates producing product with a comparable rate, hence producing a mixture of products (scenario **II**). The glycosylation with the corresponding mannuronosyl derivative also involves both the axial and equatorial glycosyl triflates, yet only the latter is a product-forming intermediate irrespective of nucleophile strength, providing the glycoside product with excellent stereoselectivity (scenario **III**). Hence, the presented NMR and computational workflow now enables the assignment of different kinetic scenarios, which bolsters our understanding of glycosylation reactions that proceed according to the Curtin–Hammett principle.

**Scheme 1:**
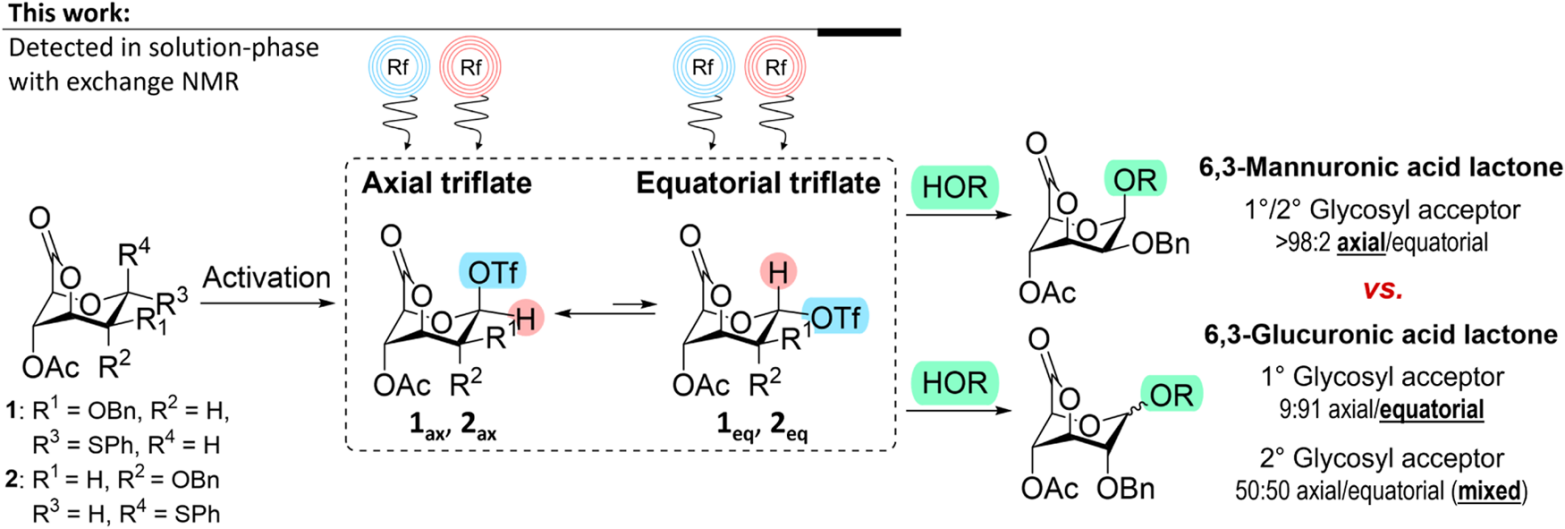
Stereodirecting Glycosylation Reaction Intermediates of 6,3-Mannuronic Acid Lactone **1** and 6,3-Glucuronic Acid Lactone **2** ^a^

a In this work, we use EXSY and CEST NMR to detect which intermediate is responsible for the observed selectivity.

## Results and Discussion

### Glycosyl Donor Synthesis

First, we prepared glycuronosyl lactones **1** and **2**, which were activated *in situ* to generate their corresponding glycuronosyl triflate reaction intermediates (**1**_**ax**_ and **2**_**ax**_, Scheme 1). Previously, we performed VT-NMR studies with glycosyl sulfoxide derivatives. Here, we chose thioglycoside derivatives **1** and **2** because the NMR spectra of the activated thioglycoside derivatives were very similar to the activated glycosyl sulfoxides (Figures S2 and S3), while the latter required an extra synthetic step. Mannuronosyl lactone **1** was prepared starting from d-mannose (Scheme S2). The synthesis was executed according to previously reported procedures.^30,34^ Glucuronosyl lactone **2** was prepared in an analogous procedure starting from peracetylated d-glucose (Scheme S3).

### VT-NMR

Next, VT-NMR experiments were conducted to characterize the reaction intermediates. Glycuronosyl donors **1** and **2** were dissolved in CD_2_Cl_2_ and activated using diphenyl sulfoxide (Ph_2_SO) (1.1 equiv), triflic anhydride (Tf_2_O) (1.5 equiv) in the presence of the non-nucleophilic base tri*tert*-butylpyrimidine (TTBP) (2.5 equiv) at −80 °C and were then heated to −60 °C. Activation of both donors was complete within 15 min (Figures S4 and S5). In both mannuronosyl and glucuronosyl derivatives, the major species formed was the axial glycuronosyl triflate (**1**_**ax**_ and **2**_**ax**_), consistent with earlier observations.^34^ Subsequently, we studied the reactivity of the axial glycuronosyl triflates using ^19^F EXSY by measuring the exchange between **1**_**ax**_ or **2**_**ax**_ and unbound triflate (^−^OTf) at varying temperatures (Figures S19 and S27). Magnetization from the excited axial glycuronosyl triflate’s CF_3_ resonance was transferred to ^–^OTf through ZZ-exchange (Figure 2A). Plotting the degree of magnetization transfer against mixing time provided the observed triflate dissociation rate, R_ax→OTf, EXSY_, as determined with EXSY (Figure 2B). R_ax→OTf, EXSY_ is the normalized triflate dissociation rate that is expected for the two possible mechanisms in which the triflate can dissociate from the glycoside, namely, through a unimolecular or bimolecular process (Figure 2C). Notably, R_ax→OTf, EXSY_ could only be measured in a narrow temperature range because (1) the glycuronosyl triflate dissociation is too slow to be detected with EXSY below −40 °C; and (2) the glycuronosyl triflates decompose above −20 °C. The lower detection limit is dictated by the T_1_-relaxation rate of the observed nucleus; exchange should occur faster compared to the relaxation of the nuclei. We previously determined that the minimal exchange rate is about 0.1 s^–1^.^28^ The obtained glycuronosyl triflate dissociation rates (R_ax→OTf,EXSY_) were about 3-fold faster for mannuronoside **1** than glucuronoside **2** (Figure 2B), which prompted us to investigate the triflate dissociation mechanism. Multiple mechanisms of glycuronosyl triflate dissociation can be expected, namely: (1) formation of a solvent-separated ion pair (SSIP) by dissociation of ^–^OTf; (2) C-4 neighboring group participation (NGP), which yields a dioxepanium ion; or (3) direct displacement of the axial triflate by free ^–^OTf, affording the equatorial glycuronosyl triflate (Figure 2C). Notably, the kinetics of the first two pathways are independent of [^−^OTf] while the kinetics of the latter mechanism would be first order in [^−^OTf]. Therefore, we assessed the axial glycuronosyl triflate dissociation rate at different ^–^OTf concentrations using sequential additions of 1 M tetrabutylammonium triflate (TBAT) in CD_2_Cl_2_ (Figures 2D and S28–S35). Both the mannuronosyl and glucuronosyl species demonstrated first-order [^−^OTf]-dependence. We have previously shown that C-4 acyl NGP participation is observed, in the absence of counterions, in the gas-phase.^34^ However, the solution phase experiments (Figure 2) suggest that the formation of an equatorial glycuronosyl triflate rather than an oxocarbenium or dioxepanium ion formation is responsible for triflate dissociation. This is consistent with a recent report by Crich and co-workers who demonstrated that the axial C-4 acyl group on galactosides adopts a rotamer that disfavors C-4 NGP.^35,36^ Only the introduction of a C-4 methyl group in galactosides facilitated the detection of the corresponding 1,4-dioxepanium ion by increasing the C-4 acyl rotation. Hence, we conclude that for 6,3-uronic acid glycosyl donors, formation of an equatorial glycuronosyl triflate is responsible for triflate dissociation in the solution phase.

**Figure 2:**
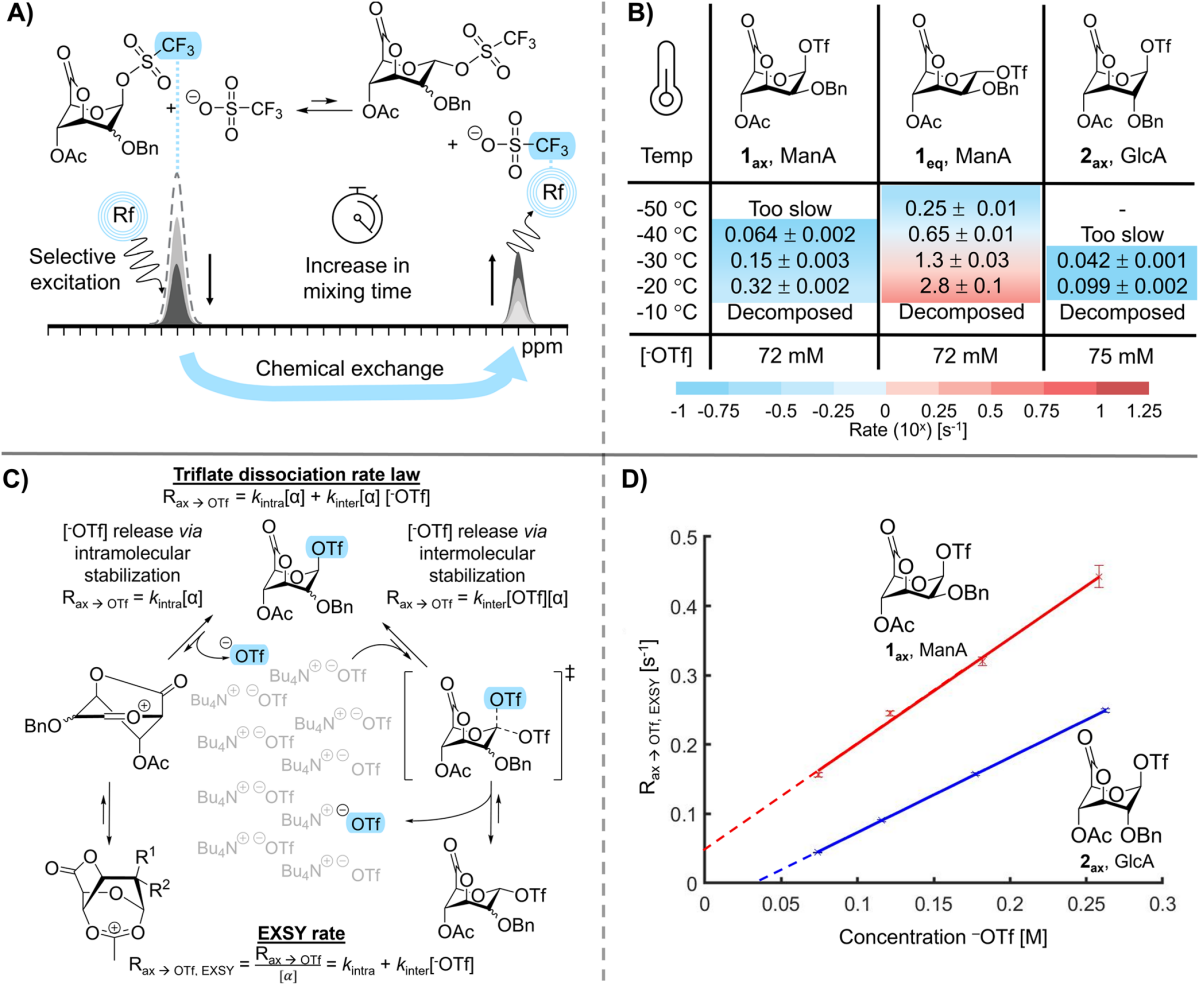
^19^F EXSY NMR experiments of lactones **1** and **2**. (A) Illustration of ^19^F EXSY NMR experiments on glycosyl triflates. (B) Dissociation rate kinetics of **1**_**ax**_ and **2**_**ax**_ as a measure of the triflate reactivity at different temperatures. (C) Theoretical unimolecular and bimolecular mechanisms of triflate dissociation in 6,3-uronic acid lactones. (D) Triflate dissociation rate kinetics of lactones **1** and **2** at −30 °C plotted against an increasing triflate concentration.

In order to confirm the existence of equatorial glycuronosyl triflates **1**_**eq**_ and **2**_**eq**_, we employed ^1^H and ^19^F CEST NMR to scan for low-abundant transient reaction intermediates.^28^ ^1^H CEST profiles were obtained by plotting the relative intensity of a selected resonance, namely, the H-1 signal belonging to the axial glycuronosyl triflate, against the degree of saturation transfer to this peak while scanning the saturation offset frequency (Figure 3A). The profiles displayed a clear difference between the mannuronosyl and glucuronosyl lactones. A low-abundance exchanging species was only found for axial mannuronosyl triflate **1**_**ax**_ at δ_H_ ≈ 5.6 ppm (Figure 3B). In contrast, no exchanging species was detected for axial glucuronosyl triflate **2**_**ax**_ (Figure 3C), even though both molecules exhibited first-order triflate dissociation kinetics. This may be explained by the detection limits of the CEST experiment, which are sensitive to variables that affect the resonance frequency difference between exchanging species (Δω) and the exchange rate between exchanging species, which must be slow on the NMR time scale (Δϖ > *k*_1_ + *k*_–1_).^25,26,37^ As the relative population of the exchanging species affects the viewing window, there is likely only a very low population of equatorial glucuronosyl triflate **2**_**eq**_, plausibly because the axial glucuronosyl triflate is more stable due to the Δ2-effect.^38,39^ ^19^F CEST profiles were generated using the CF_3_ resonance belonging to the free triflate (Figure 3D). In the manno-type lactone, the ^19^F CEST experiment shows that there are two resonances in chemical exchange with ^–^OTf, namely the axial mannuronosyl triflate **1**_**ax**_ (δ_F_ ≈ −75.7 ppm) as well as a minor species (δ_F_ ≈ −74.6 ppm) (Figure 3E). In the case of axial glucuronosyl triflate **2**_**ax**_ (δ_F_ ≈ −75.6 ppm), no other species could be detected (Figure 3F). An overlay of the ^1^H and ^19^F 1D spectra and CEST profiles revealed that the CEST dips correlated with a doublet at δ_H_ = 5.63 ppm and a ^19^F resonance at δ_F_ = −74.51 ppm, thus showing that there is a significant population of exchanging species present. As the latter is in chemical exchange with both the axial mannuronosyl triflate and free triflate, this species was tentatively assigned as the equatorial mannuronosyl triflate **1**_**eq**_.

**Figure 3:**
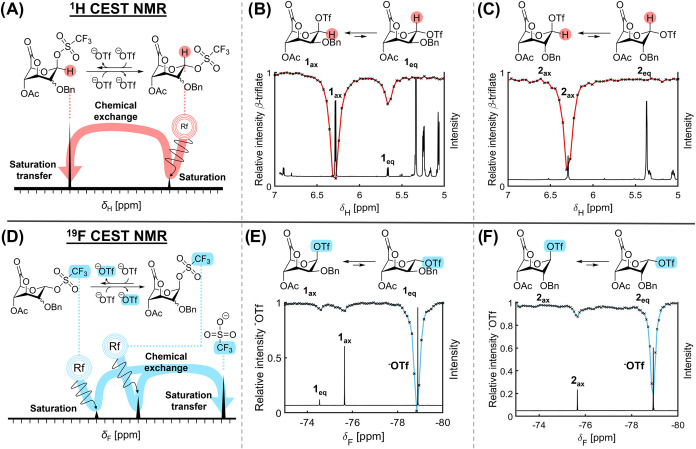
^1^H CEST NMR experiments of lactones **1** and **2** overlaid with ^1^H NMR spectra. (A) Illustration of ^1^H CEST NMR experiments on glycosyl triflates. (B) ^1^H CEST profile of activated mannuronic acid lactone **1**. (C) ^1^H CEST profile of activated glucuronic acid lactone **2**. (D) Illustration of ^19^F CEST NMR experiments on glycosyl triflates. (E) ^19^F CEST profile of activated mannuronic acid lactone **1**. (F) ^19^F CEST profile of activated glucuronic acid lactone **2**.

As the low-abundant mannuronosyl intermediate **1**_**eq**_ was detectable in ^1^H NMR, its three-bond scalar couplings (^3^ *J*_H–H_) and one-bond scalar couplings (^1^ *J*_C–H_) were measured to assign the anomeric configuration and the ring conformation of the minor species (Figures S6 and S7). The axial mannuronosyl triflate, which contains an equatorial anomeric proton, displayed a ^3^ *J*_H–H_ of 5.0 Hz and ^1^ *J*_C–H_ of 188 Hz, *versus* ^3^ *J*_H–H_ = 6.4 Hz and ^1^ *J*_C–H_ = 175 Hz in the case of the minor species. Hence, the minor exchanging species was determined to be the equatorial mannuronosyl triflate based on its large axial–axial coupling and relatively small ^1^ *J*_C–H_, which is characteristic of an axial anomeric proton as described by the Perlin effect.^40–42^ Notably, scalar couplings in **1**_**ax**_, **1**_**eq**_ and **2**_**ax**_ all deviate from values expected for a ^1^ *C*_4_-conformation, suggesting a distorted ring system. Therefore, the lowest-energy conformation was calculated using quantum-chemistry (Figures S8 and S9). Interestingly, the calculations demonstrated that O-4 is distorted and show that intermediates **1**_**ax**_ and **1**_**eq**_ acquired an *E*_4_-like envelope conformation. Dihedral angles between H-1 and H-2 were 41° and 156°, respectively, and this explains why the scalar couplings deviate from the values expected for a ^1^ *C*_4_-chair conformation in accordance with the Karplus relationship.

Having established that the minor resonance observed with ^19^F NMR belongs to the equatorial mannuronosyl triflate intermediate (**1**_**eq**_), we postulated that its population was sufficient to conduct EXSY experiments that could be used to determine the rate of dissociation (R_eq→OTf,EXSY_, Figure 2B). Notably, the triflate dissociation rate was considerably faster compared to its axial counterpart and confirmed the high reactivity of equatorial mannuronosyl triflates. Moreover, we reasoned that we could use the rates determined with EXSY, in combination with glycosylation kinetic data, to determine whether the reaction proceeds according to a Curtin–Hammett scenario.

### Reaction Kinetics

Hence, we set out to measure the reaction kinetics of product formation by reacting **1** and **2** with methyl 2,3,6-tri-*O*-benzyl-α-d-glucopyranose (**20**, secondary acceptor) or 2,3,4-tri-*O*-benzoyl-α-d-glucopyranoside (**21**, primary acceptor). Reaction progress was measured using NMR spectroscopy by identifying unique resonances in ^1^H NMR spectroscopy to track the consumption of the starting material and the formation of products over time (Figure 4A). The resonance signals of all starting materials and products could be directly correlated *via* an internal standard to their concentrations, required to fit the experimental data to kinetic models. However, studying reaction kinetics at low temperature poses issues with respect to the mixing of reagents while maintaining sample temperature control. We resolved this issue by utilizing a double-walled NMR tube (Figure 4B). This tube allows us to fill both chambers separately, cooling them both in the probe to ensure an equal temperature and allow fast mixing of the solutions by lifting the inner tube (Figure 4C). The glycosylation reactions were carried out by filling the outer tube with a CD_2_Cl_2_ solution containing the thioglycoside donor, Ph_2_SO, TTBP, and trimethyl­(4-trifluoromethylphenyl)­silane as an internal standard. The inner tube was filled with a CD_2_Cl_2_ solution containing a glycosyl acceptor. After the sample was cooled to −60 °C, Tf_2_O was added to the outer tube in order to activate the thioglycoside donor. After activation, the tube was lifted from the probe and the inner tube was lifted while the sample was floating on cold compressor gas. The lifting of the inner tube ensures mixing of the activated donor and glycosyl acceptor solutions. The sample was reinserted into the probe within 15–20 s after ejection. After locking and shimming, ^1^H NMR spectra were acquired typically every 5 min, with the exception of fast reactions in which case a spectrum was acquired every two minutes. Acquisition of one spectrum took about two minutes (80 s for fast reactions), followed by an interscan delay of at least 40 s to update the shims. Key ^1^H NMR signals of the axial glycuronosyl triflate, equatorial glycuronosyl triflate, axial glycoside, and equatorial glycoside products were measured to establish the relative abundance of each compound in time (Figures S10 and S17). Reactions were typically carried out at −60 °C, except for glycosylation of the glucuronosyl donors with the secondary acceptor; this reaction proceeded too slowly to allow sufficient conversion within a reaction time of 8 h.

**Figure 4:**
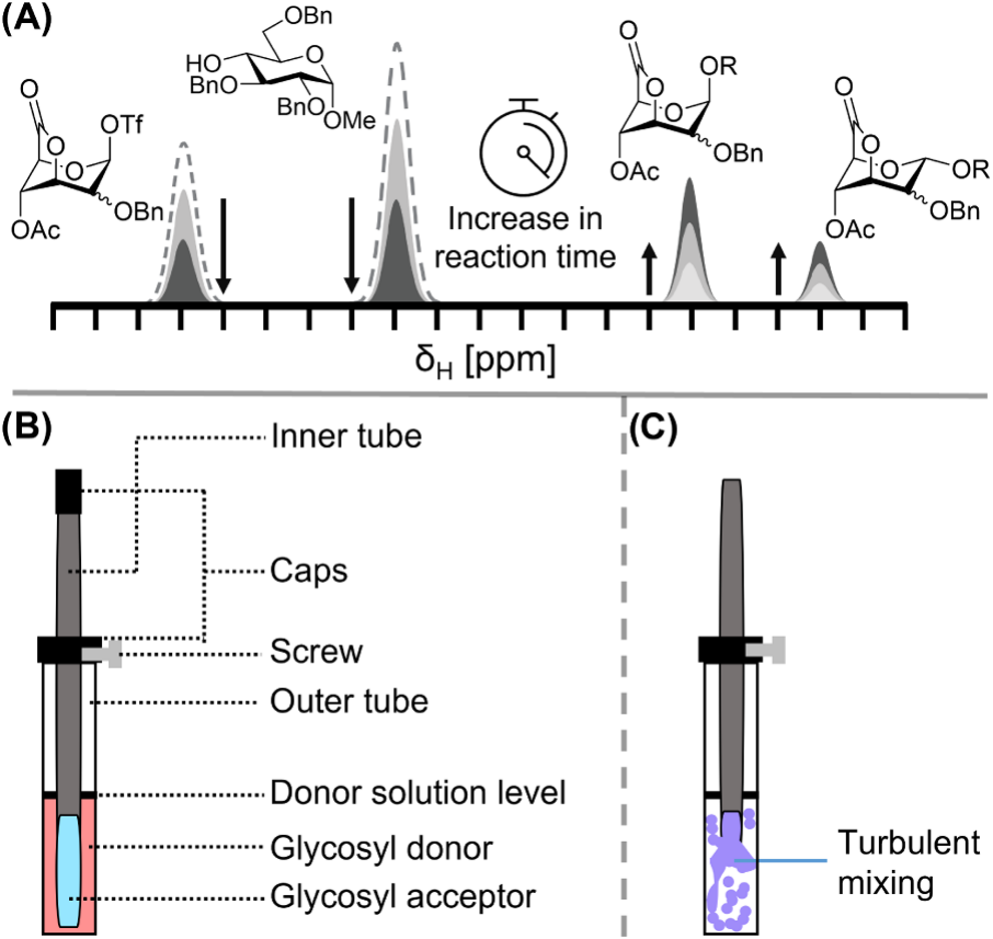
Graphical visualization of reaction kinetic experiments. (A) Key NMR signals tracked over time. (B, C) Schematic representation of the reaction monitoring tube used for the experiment and its mixing mechanism.

As expected, the glycosylation reactions with mannuronosyl donor **1** provided the axial product with both primary and secondary glycosyl acceptors (>98:2 ax/eq). Glycosylations with glucuronosyl donor **2** provided no selectivity (50:50 ax/eq) with the secondary acceptor **20** and predominantly equatorial product (9:91 ax/eq) with the primary acceptor **21**. Next, we used the experimental data to develop a computational kinetic model to fit the data and extract product-forming pathways. The following assumptions were made to set up the kinetic model that describes the experimental results. As both axial glycuronosyl triflates **1**_**ax**_ and **2**_**ax**_ exhibited first-order triflate release kinetics, we assumed that both are in rapid equilibrium with the corresponding equatorial glycuronosyl triflates **1**_**eq**_ and **2**_**eq**_. This was supported by the characterization of the former species in the mannuronosyl case. We assumed that both species react in a bimolecular fashion to provide the respective β- and α-products. Furthermore, the reaction of glycosyl acceptor with excess Tf_2_O was observed, and hence, this side reaction was also included in the model as it affects the glycosyl acceptor and free triflate concentrations.

In the case of mannuronosyl donor **1**, the concentration of axial mannuronosyl triflate (**1**_**ax**_), equatorial mannuronosyl triflate (**1**_**eq**_), equatorial products (**22**_**eq**_ and **23**_**eq**_), and byproducts **26** or **27** could be measured experimentally during the glycosylation reaction. Furthermore, the rate of axial–equatorial glycuronosyl triflate interconversion at the reaction temperature was extrapolated from the EXSY data (Figure 3). To further analyze this data, we attempted to accurately describe product formation by the computational kinetic model, specifically by fitting the rate constants of the equatorial-axial interconversions (*k*_1_, *k*_2_) as well as equatorial glycuronosyl triflate (*k*_3_), axial product (*k*_4_), and side product (*k*_5_) formation. In the case of glucuronosyl donor **2**, the same modeling approach was used. However, the concentration of equatorial glucuronosyl triflate could not be measured experimentally, and therefore, its concentration was also modeled. In the fitting procedure, the unknown parameters (*i.e.*, rate constants and unknown starting concentrations) were optimized to fit the kinetic model to the data by minimizing the sum of the squared residuals, using the Matlab *lsqnonlin* solver with 100 different initial parameter sets (Supporting Information).

The kinetic model provided an excellent description of the experimental data (Figures 5B and S18). We note that for all four reactions, the computed reaction rates of the equatorial-axial glycuronosyl triflate interconversions (with rate constants *k*_1_, *k*_2_) were significantly higher than those of the product-forming glycosylation reactions (with *k*_3_, *k*_4_). This is consistent with a Curtin–Hammett scenario, where glycuronosyl triflate interconversion is faster than the product-forming steps. Therefore, differences in product distribution solely depend on the rate difference in product formation. In the mannuronosyl case, only the axial glycoside product was formed, irrespective of the nucleophile used. Mannuronosyl triflate interconversion was predicted to be much faster than product formation, and hence a classic Curtin–Hammett scenario where the major product derives from the minor reaction intermediate (scenario **III**). In the glucuronosyl case, the rates of glucuronosyl triflate interconversion were also modeled to be faster than those of product formation. The product-forming rates with a secondary glycosyl acceptor are similar, thus leading to poor stereoselectivity (scenario **II**). In the case of the more reactive primary glycosyl acceptor, the kinetic scenario is different, as the reaction with the axial glucuronosyl triflate is faster, thus leading to the predominant formation of the equatorial glycoside product (scenario **I**). These results plausibly explain the kinetic scenario differences and highlight that the rate difference in product-forming steps drives stereoselectivity as glycuronosyl triflate interconversion was faster than product formation in all cases.

**Figure 5:**
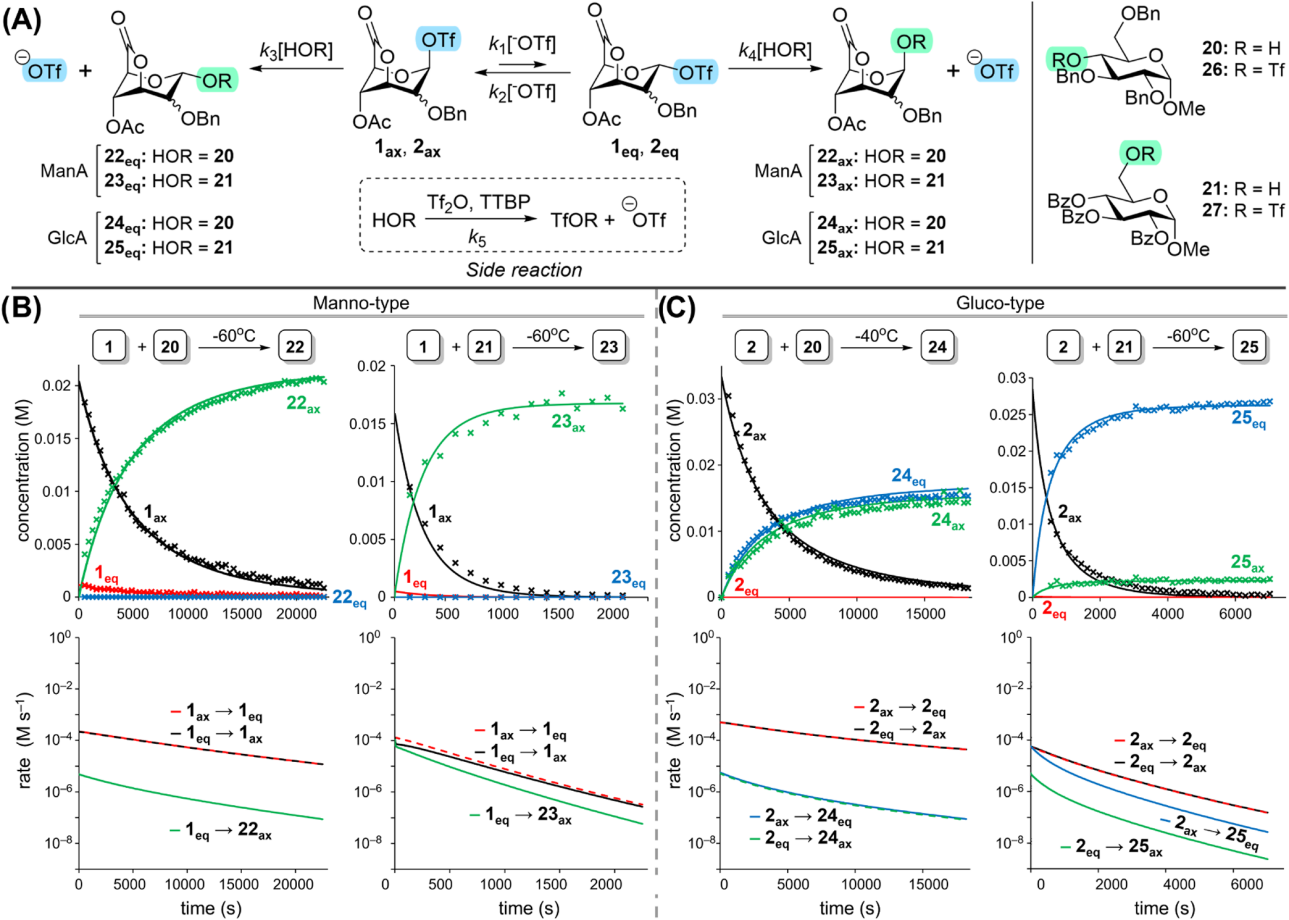
Reaction kinetics of preactivated donors **1** and **2**. Donors were activated using Ph_2_SO/Tf_2_O/TTBP in CD_2_Cl_2_. (A) General reaction scheme featuring nucleophilic displacement of the axial or equatorial glycosyl triflate intermediates. (B, C) Concentrations of intermediates (**1**_**ax**_, **1**_**eq**_; **2**_**ax**_, **2**_**eq**_) and products (**22**_**eq**_–**25**_**eq**_; **22**_**ax**_–**25**_**ax**_) *vs* time, acquired by time-dependent ^1^H NMR for 4 different glycosylation reactions (top row). The solid lines represent the best predictions of the kinetic model that was fitted to the data. Next, the kinetic model allowed to predict the rates of the equatorial-axial interconversion (**1**_**ax**_/**2**_**ax**_ → **1**_**eq**_/**2**_**eq**_; **1**_**eq**_/**2**_**eq**_ → **1**_**ax**_/**2**_**ax**_) as well as the glycosylation reactions (**1**_**ax**_/**2**_**ax**_ → **22**_**eq**_ – **25**_**eq**_; **1**_**eq**_/**2**_**eq**_ → **22**_**ax**_ – **25**_**ax**_) (bottom row). We note that the concentration of **2**_**eq**_ was below the detection limit of the ^1^H NMR.

### Transition State Energy Profiles

To further investigate the assignment of the kinetic scenario, we used quantum chemical calculations to determine the positions of the reaction intermediates in the energy landscape and establish the transition state (TS) energies for glycuronosyl triflate interconversion and product formation. To this end, energies and geometries were computed in dichloromethane and given relative to the corresponding axial glycuronosyl triflate ground state at PCM­(CH_2_Cl_2_) MP2/aug-cc-pVTZ//PCM­(CH_2_Cl_2_) B3LYP-GD3BJ/def2-TZVP level using ORCA (ver. 6.0.1).^43^ The MP2 single point energy was combined with the B3LYP thermal correction at −60 °C to give the Gibbs free energy. The ground state structures were confirmed by verifying the absence of imaginary frequencies. These calculations predict that the axial glycuronosyl triflate intermediate is more stable than the equatorial glycuronosyl triflate in the mannuronosyl and glucuronosyl case, as observed and consistent with stabilization by the anomeric effect of the former species (Figure 6A,C).^44–46^ Furthermore, the ground state energy difference between the axial and equatorial glycuronosyl triflate is larger for the glucuronosyl derivative (Figure 6C) than the mannuronosyl derivative (Figure 6A), consistent with the Δ2-effect in the former species due to the ^1^C_4_ conformation and axial positioning of the C-2 substituent.^38,39^ The calculated lowest-energy structure for the equatorial glucuronosyl derivative is distorted toward the E_4_ conformation, making the triflate pseudoaxial (Figure S9). The computed Boltzmann ratio of axial and equatorial glycuronosyl triflate at equilibrium is 79:21 for the mannuronosyl derivative (*vs* ∼94:6 found by integrating key ^19^F NMR signals) and 99.98:0.02 for the glucuronosyl derivative (below the detection limit in ^19^F NMR, <∼1%).

**Figure 6:**
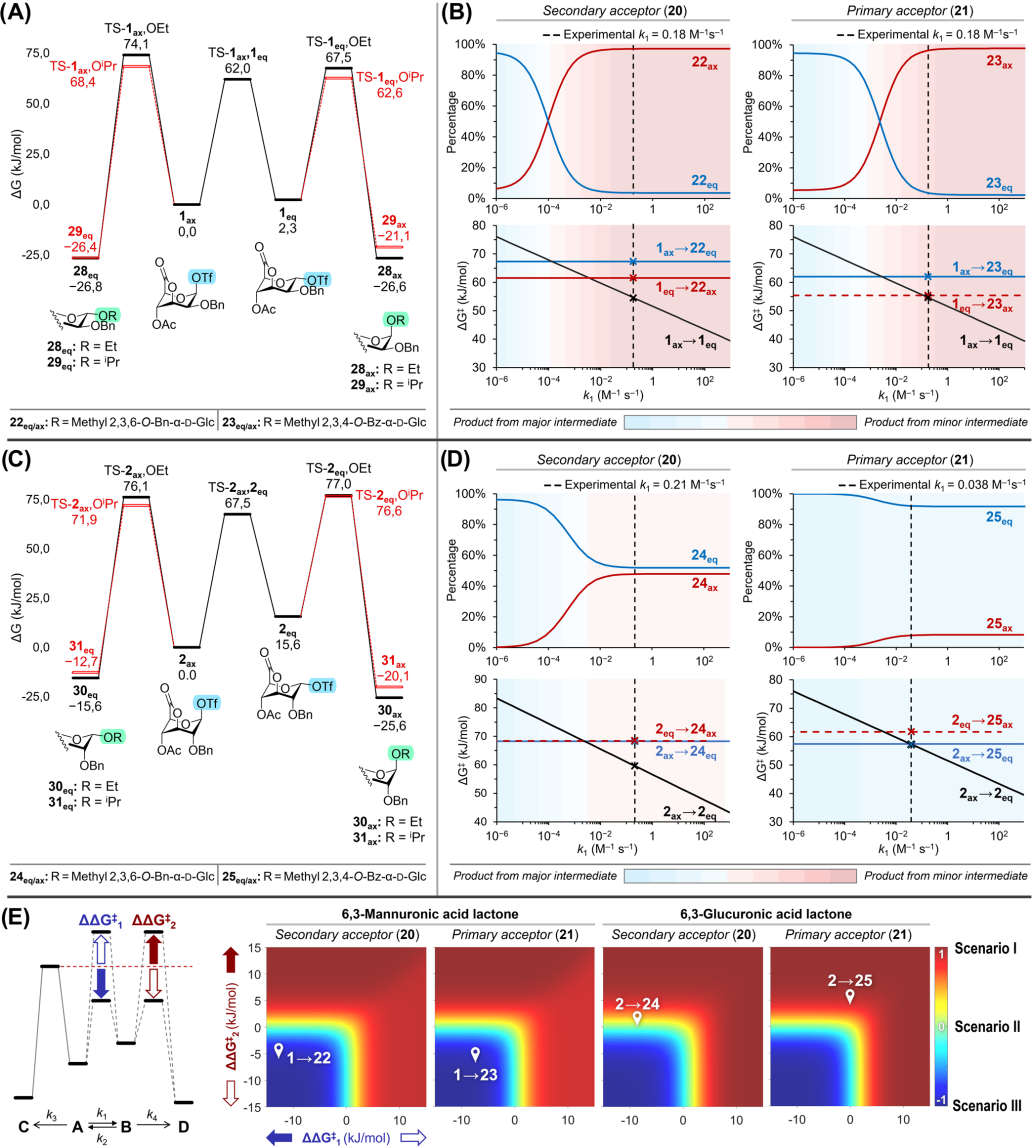
(A) Quantum-chemically computed Gibbs free energy surface in DCM (Δ*G*_DCM_) for the reaction of mannuronosyl donor **1** with EtOH (black) and ^i^PrOH (red). (B) Computed product fractions of **1** with glycosyl acceptor **20** or **21** at varying *k*_1_ (top), as well as the computed TS energy barriers at varying *k*_1_ (bottom). TS energies are derived from the rate constants found *via* the fitting of the kinetic model to the data. For the TS energy of the equatorial product-forming step, we used the difference in TS energies (ΔΔ*G*_ax‑eq_^‡^) between the respective TSs involved in the formation of the equatorial and axial product as found in the quantum chemical calculations, which was added to the TS energy of the axial product-forming reaction derived from *k*_4_. (C) Quantum-chemically computed Gibbs free energy surface in DCM (Δ*G*_DCM_) for the reaction of glucuronosyl donor **2** with EtOH (black) and ^i^PrOH (red); (D) computed product fractions of **2** with glycosyl acceptor **20** or **21** at varying *k*_1_ (top), as well as the computed TS energy barriers at varying *k*_1_ (bottom). (E) In analogy to the 4 different simulations shown in (B, D), the kinetics are simulated while varying the relative differences in ΔΔ*G*_1_^‡^ and ΔΔ*G*_2_^‡^ (left scheme). The 2D heat maps indicate the relative excess in products C and D (*i.e.*, ([C] – [D])/([C] + [D], with +1 indicating only product C formed, and −1 only product D formed). The location pins indicate the values of ΔΔ*G*_1_^‡^ and ΔΔ*G*_2_^‡^ that have been determined upon analysis of the experimental kinetic data, complemented by the values of ΔΔ*G*_ax‑eq_^‡^ found *via* the quantum chemical calculations for the reaction of mannuronosyl donor **1** (in analogy to the analysis shown in B).

Next, the TS energies of glycuronosyl triflate interconversion, as well as axial and equatorial product formation were calculated using the same level of theory. To simplify the system and save on computation time, we modeled the product-forming TSs using ethanol (EtOH) and isopropanol (^i^PrOH) as primary and secondary nucleophile substitutes, respectively (Figure 6A,C). The TS geometries were optimized to a pentacoordinate reaction center, as we assumed S_N_2-type TSs. The triflate interconversion has an anionic TS, while the product-forming TSs is neutral, with the hydroxyl hydrogen on the acceptor. The TSs were checked for the presence of a single imaginary vibrational frequency for which the vibrational mode corresponds to the substitution reaction coordinate. In all cases, the barrier toward glycuronosyl triflate interconversion was lower than that of glycoside product formation (Figure 6A,C). This suggests that the triflate anion is a good nucleophile capable of inducing rapid glycuronosyl triflate interconversion at a faster rate than glycoside product formation upon reaction with an alcohol nucleophile, which is consistent with previous computations and reports on the unexpected nucleophilicity of triflate toward electrophiles.^47,48^

Quantum chemical calculations of the product-forming TSs for the mannuronosyl case show that the barrier for equatorial glycoside formation was considerably higher than that for glycuronosyl triflate interconversion and axial glycoside formation for both glycosyl acceptors (Figure 6A). This situation would represent scenario **III**, where the minor reaction intermediate produces the majority of the product. As the glycuronosyl triflates are in rapid equilibrium according to a Curtin-Hammet scenario, the product ratio can be determined from the difference in energy of the product-forming TSs (ΔΔ*G* ^‡^) as $\frac{\left[\mathrm{Ax}\right]}{\left[\mathrm{Eq}\right]}=\exp\left(-\Delta \Delta G^{‡}/\mathrm{RT}\right)$ . The calculated stereoselectivity for mannuronosyl donor of 98:2 ax/eq and 96:4 ax/eq for the primary and secondary acceptors, respectively, is in line with the experimental selectivity of only the axial product (>98:2 ax/eq) for both acceptors (Figure 6A).

In the glucuronosyl case, interconversion of the glucuronosyl triflate proceeded with the lowest barrier consistent with a Curtin–Hammett scenario where (*k*_1_,*k*_2_ > *k*_3_,*k*_4_). However, the TS energies of axial and equatorial product formation were found to be very similar for both glycosyl acceptor types, thus resulting in poor stereoselectivity (Figure 6C). The calculated selectivities of 37:63 (ax/eq, EtOH) and 8:92 (ax/eq, ^i^PrOH at −40 °C) are in line with the mixed selectivity observed, although they deviate from the exact experimental ratios (9:91 ax/eq and 50:50 ax/eq, respectively). The deviations can be explained by the computational errors that can arise in modeling the solvent, model acceptor difference, and theory and can be expected to be larger than 4 kJ/mol.^49^ However, the mechanistic scenarios and general selectivity differences between the mannuronosyl and glucuronosyl compounds are reproduced by the quantum chemical calculation and align well with the kinetic model. The contrasting glycosylation behavior of the mannuronosyl and glucuronosyl system is reminiscent of other conformationally restricted glycosyl donors such as 4,6-benzylidene protected mannosyl and glucosyl analogues.^24,50^ In the mannuronosyl lactone system, a ^1^C_4_ conformation induced by the lactone bridge causes the C-2 substituent to adopt an equatorial conformation. Similarly, 4,6-benzylidene protected glucosides are locked in the ^4^C_1_ conformation, also positioning the C-2 substituent in an equatorial conformation (no Δ2-effect). Both compounds produce axial glycosyl triflates, which react to provide axial glycoside products, through rapid equilibration with an equatorial glycosyl triflate according to a Curtin–Hammett­(-like) scenario. In contrast, glucuronosyl lactone and 4,6-benzylidene mannosyl donors exhibit a conformation in which the C-2 position adopts an axial position (Δ2-effect).^38,39^ In these cases, the axial glycosyl triflates formed upon activation can directly react with strong nucleophiles to afford equatorial glycoside products. Hence, the stereodirecting effect of the C-2 substituent may be used to explain, predict, and design more stereoselective glycosylation reactions derived from other types of glycosyl donors.

We investigated the robustness of the computational model that describes the reaction kinetics and TS energy differences. A critical component of the Curtin–Hammett scenario is glycuronosyl triflate interconversion rates that exceed those of product formation. With the kinetic model established, we investigated how sensitive the investigated glycosylation reactions are to perturbation of the glycuronosyl triflate interconversion rates (Figure 6B,D). To this end, the rate constants of the interconversion *k*_1_ were varied to assess its effect on the predicted fractions of the axial and equatorial glycoside products. In these simulations, the other rate constants were kept at the values determined *via* the fitting procedure, and also the ratio *k*_1_/*k*_2_ was kept constant. At low values of *k*_1_, the species formed from the low-energy glucuronosyl triflate compound (*i.e.*, the major intermediate) dominates the product mixture (Figure 6B,D). In contrast, at high values of *k*_1_, the predicted fractions of axial and equatorial product reflect the difference in the energy of the TS involved in the formation of the products, such that the product with the lowest Δ*G* ^‡^ dominates the product mixture, as anticipated in the Curtin–Hammett regime. At the rate constants *k*_1_ that have been determined experimentally, we observe that the minor intermediate dominates product formation in the mannuronosyl system. In the case of the glucuronosyl system, a significant contribution of the minor intermediate to the product formation was predicted only with the secondary acceptor, as observed in the kinetic experimental data as well.

Finally, we used the kinetic model to further unravel the effect of the TS energies of the interconversion as well as the axial product-forming steps on the ratio of different products that were obtained. To this end, we kept the TS of the equatorial product-forming step (C) constant, and varied ΔΔ*G*_1_^‡^, the difference in TS energies between the interconversion (A, B) and equatorial product-forming step (Figure 6E). In addition, we varied ΔΔ*G*_2_^‡^, the difference in TS energies between the axial product formation step (D) and the equatorial product-forming step. The experimental results obtained with the mannuronosyl donor and secondary or primary acceptor, respectively, were in the regime of ΔΔ*G*_1_^‡^, ΔΔ*G*_2_^‡^ < 0, where product D is mainly formed (minor intermediate → product, scenario **III**). If ΔΔ*G*_1_^‡^ > 0 and/or ΔΔ*G*_2_^‡^ > 0, product C is mainly formed (major intermediate → product, scenario **I**), as is observed with the glucuronosyl donor and primary acceptor. Scenario **II**, where a mixture of both products C and D is formed, can be obtained with ΔΔ*G*_1_^‡^ < 0, ΔΔ*G*_2_^‡^ ≈ 0, as is experimentally observed with the glucuronosyl donor and the secondary acceptor. Intriguingly, also with ΔΔ*G*_1_^‡^ ≈ 0, ΔΔ*G*_2_^‡^ < 0, a mixture of products C and D is predicted to be formed by the kinetic model. Furthermore, the 2D heat maps show that the product type formed from the mannuronosyl donor and the secondary or primary acceptor is relatively insensitive to minor changes in either ΔΔ*G*_1_^‡^ or ΔΔ*G*_2_^‡^. For the glucuronosyl donor with the secondary acceptor, the product type will be very sensitive to minor changes in ΔΔ*G*_2_^‡^, but not to changes in ΔΔ*G*_1_^‡^, whereas for the glucuronosyl donor with the primary acceptor, an increase in ΔΔ*G*_1_^‡^ and/or a decrease in ΔΔ*G*_2_^‡^ will significantly affect the product ratio.

## Conclusions

The presented workflow leverages the power of exchange NMR techniques to establish the presence and exchange kinetics of very low-abundance glycuronosyl triflate reaction intermediates. Using a double-walled NMR tube, nucleophiles could be added in a temperature- and time-controlled manner and glycosylation reaction kinetics could be measured. The combined collection of kinetic data could be used to construct a computational reaction kinetic model and assign the kinetic scenario of four glycosylation reactions. This model shows that glycuronosyl triflate interconversion is faster than glycoside product formation, thus leading to a Curtin–Hammett-controlled glycosylation reaction. A clear difference was observed between the mannuronosyl and the glucuronosyl donors. The former affords axial glycosides, while the latter stereoselectively produced equatorial glycosides in reactions with strong nucleophiles. Quantum chemical calculations further confirmed this kinetic assignment. Finally, we expect that the presented workflow using (exchange) NMR, kinetic modeling, and quantum chemical calculations can be used to unravel the mechanistic scenarios of other glycosylation reaction types, such as those involving solvent or additive adducts, and neighboring group participation. This may lead to novel insights that allow for the design of novel, more robust methods for glycosylation reactions.

## Supplementary Material





## Notes

Experimental details; formulas; correlation of EXSY mix time to intensity selected; comparison of glycosylsulfoxide and thioglycoside activation; kinetics; quantum chemical procedures and methods; VT-NMR procedures and studies; experimental procedures; ^1^H and ^13^C NMR spectra; and supporting NMR data (PDF)
Coordinates of the quantum chemical calculations (ZIP)
