# Supplementary material for: Elucidating the Curtin–Hammett Principle in Glycosylation Reactions: The Decisive Role of Equatorial Glycosyl Triflates
Source: J Am Chem Soc. 2025 Jun 23;147(26):22597–608. doi: 10.1021/jacs.5c03519 (PMC12232320; doi:10.1021/jacs.5c03519)
Supplement: Supplementary file 1 [file ja5c03519_si_001.pdf]

Supplementary Information

Elucidating the Curtin-Hammett Principle in Glycosylation Reactions: The Decisive Role of Equatorial Glycosyl Triflates

Peter H. Moons,[‡a] Frank F.J. de Kleijne,[‡a] Teun van Wieringen,[b] Floor ter Braak,[a] Hero R. Almizori,[a] Luuk J.H. Jakobs,[a] Willem P.M. de Kleijne,[a] Floris P.J.T. Rutjes,[a] Jonathan Martens,[b] Jos Oomens,[b] Peter A. Korevaar,*[c] Paul B. White,*[a] and Thomas J. Boltje*[a]

[a] Synthetic Organic Chemistry, Institute for Molecules and Materials, Radboud University Nijmegen, Heyendaalseweg 135, 6525 AJ, Nijmegen, The Netherlands
[b] FELIX Laboratory, Institute for Molecules and Materials, Radboud University Nijmegen, Toernooiveld 7, 6525 ED, Nijmegen, The Netherlands
[C] Physical Organic Chemistry, Institute for Molecules and Materials, Radboud University Nijmegen, Heyendaalseweg 135, 6525 AJ, Nijmegen, The Netherlands
‡ These authors have contributed equally to this work.
* Corresponding authors: thomas.boltje@ru.nl; paul.white@science.ru.nl; p.korevaar@science.ru.nl.

1) Experimental NMR

Instrumentation

Variable temperature NMR (VT NMR) experiments were conducted on a Bruker 300 MHz Avance III HD nanobay equipped with a BBFO probe and on the JEOL 500 ECZ-R spectrometer equipped with a ROYAL-HFX or ROYAL probe. Low temperature VT operations were achieved with the aid of LN2 evaporator to supply the cold gas, which the probe heated to the desired temperature. The temperature for VT experiments was calibrated against a pure MeOH standard to accurately determine the probe temperature.

1.1) CEST & EXSY experiment settings

Chemical Exchange Saturation Transfer (CEST) NMR spectroscopy

CEST NMR methods were used for ^{1}H and ^{19}F NMR spectroscopy. CEST NMR profiles were recorded by incrementing the saturation over a domain of interest. ^{1}H CEST was typically performed in a window between δ_H = 9.0 to 5.0 ppm. ^{19}F CEST was typically performed in a window between δ_F = −72 to −81 ppm. Before each experiment, the 90° pulse was calibrated. A saturation field strength was chosen with regard to experimental duration, resolution, and signal intensity. High resolution (small saturation field strength) leads to weak signals and long experimental times. In contrast, low resolution (large saturation field strength) leads to strong signals and faster experiments. Typically, saturation field strengths were 20 – 40 Hz for ^{1}H CEST and 30 – 60 Hz for ^{19}F CEST. The saturation was achieved either by CW saturation (Bruker) or by pulsed saturation using laminar pulses (JEOL). Finally, typical saturation times were set to 2 – 3 seconds. The relaxation delay was set one second longer then the saturation time (3 – 4 seconds) and the number of scans were typically 2 – 4 per frequency; Two dummy scans were executed.

CEST profiles were made using 1D ^{1}H NMR or ^{19}F NMR spectra with saturation at varying positions. A spectrum with no saturation or with off-resonance saturation to both the major and minor observable signal is required as reference named $M_z(0)$. This is typically a 1D spectrum with saturation at δ_H = 9.0 ppm or δ_F = −72 ppm. The peak intensity for the axial triflate (^{1}H CEST) and $^-$OTf (^{19}F CEST) resonances, which are the reporter peaks, were determined for all individual spectra after applying phasing and baseline correction. The peak intensity of every spectrum was divided by the peak intensity of the unaltered spectrum ($M_z(0)$) in order to obtain the relative peak intensity of the main observable species as a function of saturation frequency. Plotting the relative intensity of the reporter peak as function of the saturation frequency provides the CEST profile.

Selective ^{19}F Exchange Spectroscopy (EXSY) NMR

EXSY NMR was utilized in ^{19}F NMR spectroscopy. Before each experiment, the 90° pulse was calibrated and then the selective excitation offset was set to the resonance of interest. The selection pulse typically spanned 0.2 ppm and was on-resonance with the glycosyl triflate ($\delta_F \approx -75.7$ ppm or $\delta_F \approx -74.4$ ppm for axial and equatorial triflate respectively). The power levels of the excitation pulse were calculated against the actual 90-degree pulse and the selection window. Relaxation delays were typically set to 5xT_1 of the triflates, and the number of scans were set to 8 or 16 with 2 dummy scans. The longest mix times were determined empirically so that they fit within the initial rate approximation (≈10% conversion). For experiments where the shortest mix time was >80 ms, a pulse sequence that contains a Z-gradient element was used to crush zero-quantum magnetization and clean up artifacts (Bruker: selnogp, JEOL: noesy_1d). When the shortest mix time was <<80 ms, the above experiments were performed without the Z-gradient element.

1.2) Determining exchange rates with EXSY

Initial rate approximation

The axial triflate dissociates to form triflate anion in two possible mechanisms (Eq. S1 and S2). The rate law of the corresponding reactions are described in equation S3 and S4 for the intramolecular glycosyl stabilization and intermolecular glycosyl stabilization respectively. Both processes can proceed simultaneously, hence the overall rate law could be a combination of both (Eq. S5).

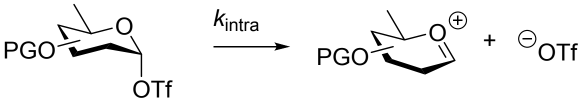

Eq. S1

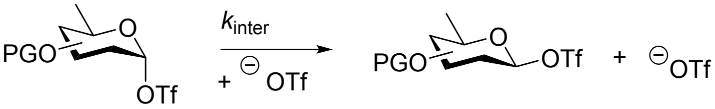

Eq. S2

$$\frac{d[OTf]}{dt} = -\frac{d[\text{axial}]}{dt} = R_{\text{axial}\rightarrow OTf} = k_{intra}[\text{axial}] \qquad \text{Eq. S3}$$

$$\frac{d[OTf]}{dt} = -\frac{d[\alpha]}{dt} = R_{\text{axial}\rightarrow OTf} = k_{inter}[\text{axial}][^{-}OTf] \qquad \text{Eq. S4}$$

$$\frac{d[OTf]}{dt} = -\frac{d[\text{axial}]}{dt} = R_{\text{axial}\rightarrow OTf} = k_{intra}[\text{axial}] + k_{inter}[\text{axial}][^{-}OTf] \qquad \text{Eq. S5}$$

By applying a selective excitation pulse on the axial triflate resonance in a selective 1D ^{19}F EXSY NMR experiment, formation of triflate anion can be measured, despite the high population triflate already present in the reaction mixture. This is possible for two main reasons: 1) the resonances of both the axial triflate and triflate anion are sufficiently separated to selectively excite the axial triflate in ^{19}F NMR, and, 2) only excited-state nuclei are detected in EXSY NMR. Varying the delay (mix time, τ_m) between the excitation of the axial triflate resonance and the spectrum acquisition allows to measure different degrees of conversion for the axial triflate into the triflate anion resonance. Eventually, by applying an 'infinitely long' mix time, the equilibrium ratio of axial triflate and triflate anion is obtained Figure S1).

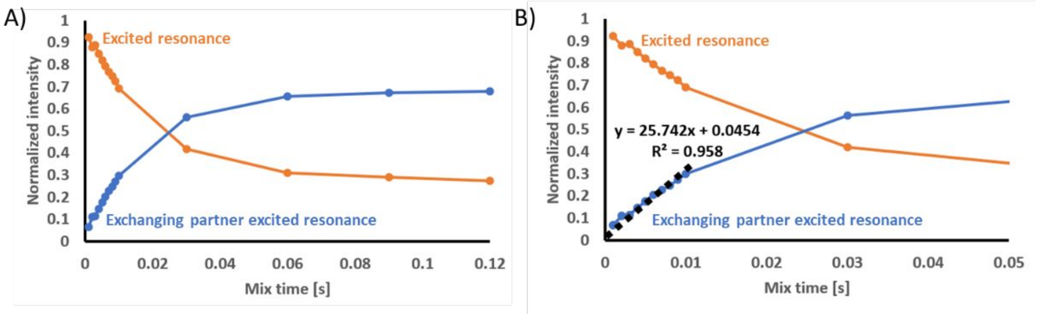

Figure S1: A) Correlation of EXSY mix time to intensity selected/excited resonance and its exchanging partner that is forming. **B)** Displaying the linear initial rate approximation.

The initial triflate formation is linear and kinetics could be described according to the initial rate approximation. Over the initial linear interval, the reaction rate is the axial triflate consumption and triflate anion formation (Eq. S6 and S7). Herein, [**axial**]$_t$ = concentration excited state α-triflate a set mix time after applying the excitation pulse on the axial triflate resonance; [**axial**]$_0$ = concentration excited state α-triflate directly after applying a selective excitation pulse on the axial triflate resonance; [$^{-}$**OTf**]$_t$ = concentration excited state $^{-}$OTf a set mix time after applying the excitation pulse on the axial triflate resonance; [$^{-}$**OTf**]$_0$ = concentration excited state $^{-}$OTf directly after applying a selective excitation pulse on the axial triflate resonance (hence, [$^{-}$**OTf**]$_0$ = 0). Substituting equation S5 into equation S6 gives the concentration excited state axial triflate in terms of concentration and mix time (Eq. S8).

$$\frac{[\mathbf{axial}]_t - [\mathbf{axial}]_0}{\tau_m} = -R_{\alpha\rightarrow OTf} \qquad \text{Eq. S6}$$

$$\frac{[^{-}\boldsymbol{OTf}]_t - [^{-}\boldsymbol{OTf}]_0}{\tau_m} = R_{\alpha\rightarrow OTf} \qquad \text{Eq. S7}$$

$$[\mathbf{axial}]_t = [\mathbf{axial}]_0 - (k_{intra}[\mathbf{axial}] + k_{inter}[\mathbf{axial}][^{-}OTf]) \times \tau_m \qquad \text{Eq. S8}$$

At the very start of the reaction, the concentration axial triflate deviates only marginally compared to the starting concentration ($[\text{axial}]_0$). In accordance to the initial rate approximation, equation S8 becomes equation S9. Additionally, within the initial rate approximation, only an axial triflate conversion of about 5-15% is recorded. Therefore, the concentration excited state triflate ($[^-\text{OTf}]$) is sufficiently small (especially compared to the bulk concentration non-excited triflate anion ($[^-\text{OTf}]$)) such that the backwards reaction can be neglected. Subsequently, dividing the equation by $[\text{axial}]_0$ simplifies the equation to S10.

$$[\text{axial}]_t = [\text{axial}]_0 - (k_{intra}[\text{axial}]_0 + k_{inter}[\text{axial}]_0[^-OTf]) \times \tau_m \quad \text{Eq. S9}$$

$$\frac{[\text{axial}]_t}{[\text{axial}]_0} = 1 - (k_{intra} + k_{inter}[^-OTf]) \times \tau_m \quad \text{Eq. S10}$$

Within NMR spectroscopy, the concentration is proportional (with a constant, c) related to the absolute integral ($\int I_x$) of the observed resonances (Eq. S11 and S12). Substituting equation S10 with S11 and S12 gives the absolute integral of the excited state axial triflate resonance as function of mix time (Eq. S13).

$$\int I_{\text{axial},t} = c \times [\text{axial}]_t \quad \text{Eq. S11}$$

$$\int I_{\text{axial},0} = c \times [\alpha\text{axial}]_0 \quad \text{Eq. S12}$$

$$\frac{\int I_{\text{axial},t}}{\int I_{\text{axial},0}} = 1 - (k_{intra} + k_{inter}[^-OTf]) \times \tau_m \quad \text{Eq. S13}$$

One complication is that T_1 relaxation occurs during the mixing time, which will reduce the absolute integral or intensity of the selected and exchanged resonances over time and plotting S13 will result in a multiexponential decay process if $k \leq T_1^{-1}$. This can be easily taken into account if the T_1 is known for each in the absence of exchange. However, if the T_1s for the species are very similar and thus experience similar rates of relaxation, then an approximation can be made where the integral of the axial triflate at t=0 ($I_{\alpha,0}$) is the sum of the integrals of the axial triflate and triflate anion at a given mix time (Eq. S14). This then allows Equation S13 to be rewritten in an internally-consistent manner where the decay is normalized by the measurable peaks for each given mix time. The slope of the plot (Eq. S16) is directly related to the rate constants of both exchange processes (Eq. S1 and S2).

$$\int I_{\text{axial},0} = \int I_{\text{axial},t} + \int I_{OTf,\,t} \quad \text{Eq. S14}$$

$$\frac{\int I_{\text{axial},t}}{\int I_{\text{axial},t} + \int I_{OTf,\,t}} = 1 - (k_{intra} + k_{inter}[^-OTf]) \times \tau_m \quad \text{Eq. S15}$$

$$Slope = k_{intra} + k_{inter}[^-OTf] \quad \text{Eq. S16}$$

The rates measured and determined by selective ^{19}F EXSY spectroscopy is, as described above, directly the slope of the normalised absolute integral of the axial triflate ($\int I_{\text{axial},t}$) versus mixing time. Hence, equation S16 is in the main text referred to as $R_{ax\rightarrow OTf,\,EXSY}$ (Eq. S17).

$$R_{ax\rightarrow OTf,EXSY} = Slope = k_{intra} + k_{inter}[^-OTf] \quad \text{Eq. S17}$$

1.3) Sample preparation VT NMR

General sample preparation characterization of intermediates and temperature dependent EXSY experiments

Glycosyl thioether donor (1.0 eq, typically ≈ 15 mg), Ph_2SO (1.1 eq), and TTBP (2.5 eq.) were dissolved in DCM-d_2 (600 μL) under Schlenk conditions. Two spherical activated molecular sieves (5 Å) were added to the NMR tube and the tube was then transferred to an analytical scale where internal standard (trimethyl(4-trifluoromethylphenyl)silane) was added. A stock solution of Tf_2O was prepared in DCM-d_2 such that upon addition of stock solution (50 μL), the desired amount Tf_2O (1.5 eq) could be added. When the NMR sample and Tf_2O stock solution were ready, the NMR tube was cooled to −80°C (dry ice/acetone bath). To the cold tube was added the freshly prepared Tf_2O stock solution (50 μL). The solution generally becomes (light) yellow upon addition of Tf_2O. The tube was quickly shaken (3x) and carefully transferred to the NMR instrument at −80°C. In the probe, the

temperature was heated to −40°C. After 30 minutes, the sample was then cooled or heated to the desired temperature at which the kinetic and characterization experiments were conducted.

Sample preparation EXSY experiments at varying TBAT concentrations

A 1.0 M solution of tetrabutylammonium triflate (TBAT) was prepared in DCM-d_2. Activated molecular sieves (4 Å) were added to the solution, after which the solution was stored under argon at −80°C. This solution was removed from the −80°C fridge one hour before the NMR experiment. NMR experiments at various concentrations of triflate anion were executed as described in the general sample preparation section. An initial amount of 500 μL DCM-d_2 was used during the experiments. The samples were activated at −60°C and then heated to -30°C. At this temperature, the sample displayed an exchange rate ($R_{ax \rightarrow OTf, EXSY}$) of about 0.1 s^{-1}, which allowed sufficient exchange at the lowest concentration. In addition, starting off at a rate at ≈0.1 s^{-1} without additional TBAT in the sample provided sufficient opportunity for the triflate dissociation rate to increase as a consequence of the increased triflate concentration, before falling out the window of EXSY NMR ($R_{ax \rightarrow OTf, EXSY}$ > 100 s^{-1}). After recording the triflate dissociation under standard conditions, the sample was removed from the probe and quickly stored in a dry ice/acetone bath (−80°C). TBAT solution was added (20 μL), the sample was quickly shaken to homogenize the solution (3x) and then carefully transferred to the probe. The sample was locked to DCM-d_2, tuned, and shimmed before performing NMR experiments. After finishing the EXSY and CEST experiments, the cycle was repeated twice by adding 30 μL and 50 μL TBAT solution. In the data workup, the internal standard was used to accurately correct the TBAT concentration.

Sample preparation glycosylation kinetics

Glycosyl donor (1.0 eq., typically ≈ 15 mg), Ph_2SO (1.1 eq.), and TTBP (2.5 eq.) were dissolved in anhydrous DCM-d_2 (400 μL) under Schlenk conditions. Glycosyl acceptor (1.5 eq.) was separately dissolved in dried DCM-d_2 (250 μL). A spherical activated molecular sieve (5 Å) was added to both solutions. After two minutes, the solutions were transferred to a 5 mm OD Reaction Monitoring system NMR Tube (purchased from Wilmad-Labglass). First, the outer tube was capped by tightening the black and white cap. Secondly, the inner tube was pushed down to assure a solid seal between the inner and outer tube. While pressing down the inner tube, the screw was secured to maintain the inner tube its position. Next, the inner tube was carefully filled with the glycosyl acceptor solution using a glass Hamilton syringe and the inner tube was directly capped. The outer tube was filled with glycosyl donor solution using a glass Hamilton syringe. The tube was placed on a scale, after which the internal standard (trimethyl(4-trifluoromethylphenyl)silane) was added to the outer tube using a glass Hamilton syringe. The tube was carefully shaken to mix the internal standard with the donor solution. Finally, a stock solution of Tf_2O was prepared in DCM-d_2 such that, upon addition of stock solution (50 μL), the desired amount Tf_2O (1.5 eq) could be added.

When the NMR sample and Tf_2O stock solution were ready, the tube was placed in a spinner cooled to −60°C (dry ice/acetone bath). Freshly prepared Tf_2O stock solution (50 μL) was added to the outer tube using a glass Hamilton syringe. The outer tube solution generally becomes (light) yellow upon addition of Tf_2O. The tube was carefully quickly shaken (3x) and was then transferred to the NMR with a probe temperature of -40°C. After 30 minutes, the sample was cooled to the desired temperature (either −60 °C or held at −40 °C), after which the system was shimmed and tuned to ^{19}F. The sample was lifted from the probe; the inner tube its cap was removed; the screw was loosened; the innertube was lifted; the inner tube was pushed down and up for two times to assure better mixing; the innertube was secured using the screw having the Teflon tip ≈7 cm above the bottom of the inner tube; and the sample was inserted again. Typically this process took 15 – 20 seconds while the tube was floating above the instrument on cold gas. The sample was locked to the solvent, shimmed and a ^{19}F spectrum was recorded. Subsequently, a ^{1}H kinetic sequence was recorded. The ^{1}H kinetic sequence recorded a ^{1}H spectrum every five minutes. The acquisition time took 115 seconds followed by a 185 seconds interscan delay. The relaxation delay was set to 10 seconds; number of scans 8; number of dummy scans 1; acquisition time was 4.4 seconds. The number of repetitions was set such that an experiment took about 8 hrs. During the interscan delay, shims were updated to maintain good shimming over the course of the experiment. After the glycosyl triflate was fully consumed (>95 %), the kinetic sequence was stopped, the sample was re-shimmed, and a ^{1}H and HSQC (coupled and decoupled) NMR experiments were performed in order to derive the final product ratio and product stereochemistry.

In case of mannuronic acid donor x with the primary acceptor, four scans were used for the ^{1}H experiments. In order to most accurately determine the population of the equatorial triflate at any timepoint ^{19}F NMR was used. ^{1}H and ^{19}F spectra were subsequently recorded in a repetition time of 2.5 minutes.

2) Kinetic model

Fitting the kinetic model to kinetic data of glycosylation reactions with donors 1 and 2

First, we describe the kinetic model that has been fitted to the kinetic data of the glycosylation reactions. To this end, the carbohydrates are labelled **c**, **d**, **HOR**, **eq** and **ax** as shown in the scheme below:

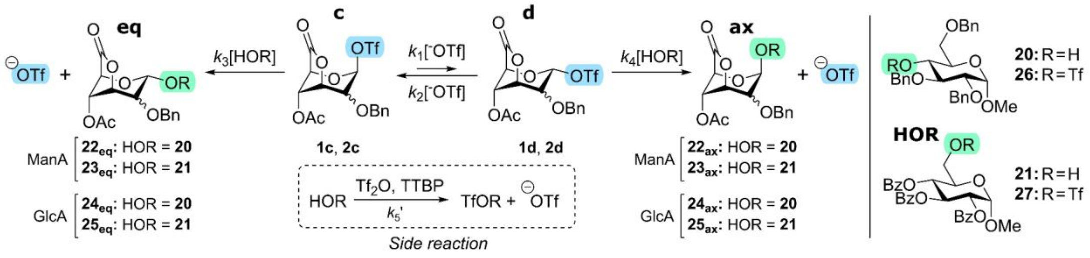

Scheme S1: General reaction scheme featuring the nucleophilic displacement of glycosyl triflate intermediates.

Next, we define the reaction rate equations via the rate constants k_1, k_2, k_3, k_4 and k_5':

$$\frac{d[\mathbf{c}]_t}{dt} = k_2[\mathbf{d}]_t[^{-}\mathbf{OTf}]_t - k_3[\mathbf{c}]_t[\mathbf{HOR}]_t - k_1[\mathbf{c}]_t[^{-}\mathbf{OTf}]_t \quad \text{Eq. S18}$$

$$\frac{d[\mathbf{d}]_t}{dt} = k_1[\mathbf{c}]_t[^{-}\mathbf{OTf}]_t - k_4[\mathbf{d}]_t[\mathbf{HOR}]_t - k_2[\mathbf{d}]_t[^{-}\mathbf{OTf}]_t \quad \text{Eq. S19}$$

$$\frac{d[\mathbf{HOR}]_t}{dt} = -k_5'[\mathbf{Tf_2O}][\mathbf{HOR}]_t - k_3[\mathbf{c}]_t[\mathbf{HOR}]_t - k_4[\mathbf{d}]_t[\mathbf{HOR}]_t \quad \text{Eq. S20}$$

$$= -k_5[\mathbf{HOR}]_t - k_3[\mathbf{c}]_t[\mathbf{HOR}]_t - k_4[\mathbf{d}]_t[\mathbf{HOR}]_t$$

$$\frac{d[\mathbf{eq}]_t}{dt} = k_3[\mathbf{c}]_t[\mathbf{HOR}]_t \quad \text{Eq. S21}$$

$$\frac{d[\mathbf{ax}]_t}{dt} = k_4[\mathbf{d}]_t[\mathbf{HOR}]_t \quad \text{Eq. S22}$$

$$\frac{d[^{-}\mathbf{OTf}]_t}{dt} = k_5[\mathbf{HOR}]_t + k_3[\mathbf{c}]_t[\mathbf{HOR}]_t + k_4[\mathbf{d}]_t[\mathbf{HOR}]_t \quad \text{Eq. S23}$$

Here, we assume that the concentration of $\mathbf{Tf_2O}$ stays constant over the course of the reaction, such that k_5'[$\mathbf{Tf_2O}$] can be defined as k_5.

In the fitting procedure, the unknown parameters (i.e. rate constants and unknown starting concentrations, *vide infra*) are optimized to fit the kinetic model to the data, using Matlab. The optimized parameters are found via a non-linear least-squares analysis where the sum of the squared residuals Σ_{res} is minimized using the Matlab *lsqnonlin* solver. To avoid the fitting program getting trapped in a local minimum, we defined 100 different initial parameter sets. We took the best fit – i.e., with the lowest Σ_{res}, min(Σ_{res}) – as the final solution for the values of the fitted parameters. Furthermore, we have included the results of fits with $\Sigma_{res} \leq 1.05 \cdot \min(\Sigma_{res})$ in the box whisker plots featuring the spread of the predicted rate constants.

The different initial parameter sets are defined using a latin hypercube sampling method (Matlab function *lhsdesign*). Next, the differential equations (S18 – S23) are solved in Matlab using the *ode15s* solver, where starting values of the concentrations at t = 0 (i.e. [**c**]$_0$, [**d**]$_0$, [**HOR**]$_0$, [**eq**]$_0$, [**ax**]$_0$ and [$^{-}$**TfO**]$_0$) are either provided as a pre-determined value, or included as an unknown parameter to be optimized in the fitting procedure. In Table S1, we have summarized for the fitting results shown in Figure 4B the parameter values corresponding to the best fits (displayed in bold), as well as the bandwidth of the initial parameter sets defined via the latin hypercube sampling method, displayed as *[10^p – 10^q],* and the lower and upper boundaries to the respective parameter in the fitting procedure, displayed as *{p – q}*. The values of the parameters that have been fixed in the respective fitting procedure are displayed in the grey boxes.

Table S1: Parameter values corresponding to the fitting results shown in Figure 5B.

**Parameter**	**1 → 22**	**1 → 23**	**2 → 24**	**2 → 25**
k_1 (M^{-1} s^{-1})	0.17902	0.17902	0.21	0.03838
k_2 (M^{-1} s^{-1})	k_1/0.0573	k_1/0.0573	*[10^{1} – 10^{5}]*<br>*{0 – ∞}*<br>**8.71□10^{3}**	([**c**]$_0$/[**d**]$_0$)□k_1 = 1.68□10^{2}
k_3 (M^{-1} s^{-1})	0	0	*[10^{-3} – 10^{0}]*<br>*{0 – ∞}*<br>**2.45□10^{-3}**	*[10^{-2} – 10^{1}]*<br>*{0 – ∞}*<br>**0.0371**
k_4 (M^{-1} s^{-1})	*[10^{-3} – 10^{0}]*<br>*{0 – ∞}*<br>**0.0629**	*[10^{-1} – 10^{2}]*<br>*{0 – ∞}*<br>**2.04**	*[10^{0} – 10^{3}]*<br>*{0 – ∞}*<br>**94.0**	*[10^{0} – 10^{3}]*<br>*{0 – ∞}*<br>**14.7**
k_5 (s^{-1})	*[10^{-6} – 10^{0}]*<br>*{0 – ∞}*<br>**1.18□10^{-5}**	*[10^{-6} – 10^{0}]*<br>*{0 – ∞}*<br>**1.94□10^{-4}**	*[10^{-6} – 10^{0}]*<br>*{0 – ∞}*<br>**2.80□10^{-5}**	*[10^{-6} – 10^{0}]*<br>*{0 – ∞}*<br>**5.85□10^{-5}**
[**c**]$_0$ (M)	*[10^{-3} – 10^{-1}]*<br>*{0.017 – 0.04}*<br>**0.0204**	*[10^{-3} – 10^{-1}]*<br>*{0.01 – 0.03}*<br>**0.0158**	*[10^{-3} – 10^{-1}]*<br>*{0.025 – 0.05}*<br>**0.0333**	*[10^{-3} – 10^{-1}]*<br>*{0.01 – 0.04}*<br>**0.0285**
[**d**]$_0$ (M)	[**c**]$_0$□0.0573	[**c**]$_0$□0.0573	[**c**]$_0$□(k_1/k_2)	*[10^{-5} – 10^{-3}]*<br>*{0 – 0.0003}*<br>**6.53□10^{-6}**
[**HOR**]$_0$ (M)	*[10^{-3} – 10^{-1}]*<br>*{0.06 – 0.1}*<br>**0.0669**	*[10^{-3} – 10^{-1}]*<br>*{0.045 – 0.08}*<br>**0.0577**	*[10^{-3} – 10^{-1}]*<br>*{0.055 – 0.15}*<br>**0.0700**	*[10^{-3} – 10^{-1}]*<br>*{0.035 – 0.06}*<br>**0.0540**
[**eq**]$_0$ (M)	0	0	0	0
[**ax**]$_0$ (M)	0	0	0	0
[$^{-}$**OTf**]$_0$ (M)	*[10^{-3} – 10^{-1}]*<br>*{0.02 – 0.065}*<br>**0.0608**	*[10^{-3} – 10^{-1}]*<br>*{0.01 – 0.055}*<br>**0.0456**	*[10^{-3} – 10^{-1}]*<br>*{0.02 – 0.08}*<br>**0.0730**	*[10^{-3} – 10^{-1}]*<br>*{0.04 – 0.07}*<br>**0.0514**

The time-dependent rates of the reactions **c** → **d**; **d** → **c**; **c** → **eq** and **d** → **ax**, $R(\mathbf{c} \rightarrow \mathbf{d})_t$, $R(\mathbf{d} \rightarrow \mathbf{c})_t$, $R(\mathbf{c} \rightarrow \mathbf{eq})_t$ and $R(\mathbf{d} \rightarrow \mathbf{ax})_t$, respectively, are calculated in M s^{-1} via:

$$R(\mathbf{c}{\rightarrow}\mathbf{d})_t = k_1[\mathbf{c}]_t[^{-}\mathbf{OTf}]_t \quad \text{Eq. S24}$$
$$R(\mathbf{d}{\rightarrow}\mathbf{c})_t = k_2[\mathbf{d}]_t[^{-}\mathbf{OTf}]_t \quad \text{Eq. S25}$$
$$R(\mathbf{c}{\rightarrow}\mathbf{eq})_t = k_3[\mathbf{c}]_t[\mathbf{HOR}]_t \quad \text{Eq. S26}$$
$$R(\mathbf{d}{\rightarrow}\mathbf{ax})_t = k_4[\mathbf{d}]_t[\mathbf{HOR}]_t \quad \text{Eq. S27}$$

To further assess the rate constant values that were found through the fitting procedures, we calculated the production rate of the compounds **eq** and **ax** via the steady state approximation, indicated as *SSA_ax* and *SSA_eq* in Figure S18. Via the steady state approximation on the rate equation of **c**, i.e. d[**c**]$_t$/dt = 0, we find that:

$$[\mathbf{c}]_t = \frac{k_2[\mathbf{d}]_t[^{-}\mathbf{OTf}]_t}{k_3[\mathbf{HOR}]_t + k_1[^{-}\mathbf{OTf}]_t},$$

and hence, at t = 0:

$$SSA_{eq} = k_3[\mathbf{HOR}]_t[\mathbf{c}]_t = \frac{k_3[\mathbf{HOR}]_t[k_2[\mathbf{d}]_t[^{-}\mathbf{OTf}]_t}{k_3[\mathbf{HOR}]_t+k_1[^{-}\mathbf{OTf}]_t}. \quad \text{Eq. S28}$$

In analogy, via d[**d**]$_t$/dt = 0, we find that:

$$[\mathbf{d}]_t = \frac{k_1[\mathbf{c}]_t[^{-}\mathbf{OTf}]_t}{k_4[\mathbf{HOR}]_t + k_2[^{-}\mathbf{OTf}]_t},$$

and hence

$$SSA_{ax} = k_4[\mathbf{HOR}]_t[\mathbf{d}]_t = \frac{k_4[\mathbf{HOR}]_t k_1[\mathbf{c}]_t[^{-}\mathbf{OTf}]_t}{k_4[\mathbf{HOR}]_t+k_2[^{-}\mathbf{OTf}]_t}. \quad \text{Eq. S29}$$

In Figure 6BD, we assessed the fraction of the axial and equatorial products, as predicted by the kinetic model upon varying the rate constant k_1 involved in the interconversion reaction in the range [10^{-6} - 10^3] M^{-1} s^{-1}. For the other rate constants and starting concentrations, we used the values as mentioned in Table S1. Here, we note that $k_2 = k_1/K_{12}$, such that the ratio between k_1 and k_2 was kept constant while varying the value of k_1. For the simulations of the reactions of mannuronosyl donor **1**, we assumed $k_5 = 0$.

Via the Eyring equation, assuming $\kappa = 1$, we approximated the ΔG‡ values of the transition states via the respective rate constants:

$$k = \frac{\kappa k_B T}{h} exp\left(-\frac{\Delta G^‡}{RT}\right) = Aexp\left(-\frac{\Delta G^‡}{RT}\right), \quad \text{Eq. S30}$$

such that

$$\Delta G^‡(\mathbf{2_{ax}}{\rightarrow}\mathbf{prod_{eq}}) = RT(ln\,A - ln\,k_3); \quad \text{Eq. S31}$$

\`

$$\Delta G^‡(\mathbf{1/2_{ax}}{\rightarrow}\mathbf{1/2_{eq}}) = RT(ln\,A - ln\,k_1);\ and \quad \text{Eq. S32}$$

$$\Delta G^‡(\mathbf{1/2_{eq}}{\rightarrow}\mathbf{prod_{ax}}) = RT(ln\,A - ln\,k_4) + RT\left(ln\frac{1}{K_{12}}\right), \quad \text{Eq. S33}$$

where the energy level of the **1/2ax** state is defined at 0.0 kJ/mol. Here, we note that eq. 32 and 33 were used for the reactions of mannuronosyl donor **1** as well as glucuronosyl donor **2**. However, for the ΔG‡ value for the reaction $\mathbf{1_{ax}}$ → $\mathbf{prod_{eq}}$, we used the difference $\Delta\Delta G^‡_{ax\text{-}eq}$ between the respective transition states involved in the formation of $\mathbf{prod_{eq}}$ and $\mathbf{prod_{ax}}$, as found in the quantum-chemical simulations, such that

$$\Delta G^‡(\mathbf{1_{ax}} \rightarrow \mathbf{prod_{eq}}) = \Delta G^‡(\mathbf{1_{eq}} \rightarrow \mathbf{prod_{ax}}) + \Delta\Delta G^‡_{ax\text{-}eq}. \quad \text{Eq. S34}$$

In Figure 6E, we simulated the relative excess in products **C** and **D**, i.e. ([**C**] – [**D**])/ ([**C**] + [**D**])$_{equil}$, that was obtained at the completion of the reaction (equilibrium), while varying the transition states of the interconversion and the axial product formation with respect to the transition state of the equatorial product formation (i.e. $\Delta\Delta G^‡_1$ and $\Delta\Delta G^‡_2$, respectively). The transition state for the equatorial product formation was found via eq. 31 (glucuronosyl

donor **2**, using k_3 found via the fitting procedure) and eq. 34 (mannuronosyl donor **1**, by combining the value of k_4 found via the fitting procedure with $\Delta\Delta G^{\ddagger}_{ax\text{-}eq}$ from the quantum-chemical calculations). The values of the starting concentrations and $K_{12} = k_1/k_2$ were used as shown in Table S1. For all reactions, we assumed $k_5 = 0$. The other rate constants were calculated via the Eyring equation (eq. 30), using the respective transition state $\Delta G^{\ddagger}$. For k_4, we subtracted ΔG_{12}, i.e. the Gibbs free energy difference between preactivated donors in the **eq** and **ax** state (which is set at 0.0 kJ/mol), from $\Delta G^{\ddagger}$(**1/2$_{eq}$ → prod$_{ax}$**). In the simulations, both $\Delta\Delta G^{\ddagger}_1$ and $\Delta\Delta G^{\ddagger}_2$ have been varied in the range [-15, 15] kJ/mol in 99 linear steps, such that the results of 100x100 simulations are presented in the 2D heat maps in Figure 6E.

3) Computational method

The conformational space was explored with the software package CREST[1] version 2.12 using the GFN2-xTB method.[2] The distance matrix for all pairs of conformers (excluding hydrogens) up to 40 kJ/mol from the lowest energy conformation found in CREST was evaluated using the RDKit AllChem.GetBestRMS algorithm. The 60 most unique conformers were subsequently selected by hierarchical clustering of the distance matrix and further optimized using Gaussian 16[3] revision C.02 at PCM(dichloromethane)-B3LYP/6-31++G(d,p) level of theory, followed by a vibrational analysis at the same level of theory and a single point energy calculation at PCM(dichloromethane)-MP2/6-31++G(d,p) level of theory. Initial guesses for the transition state were made by combining the structure of the reactant and product and manually changing the distances and angles around the C_1 atom.

Every structure was further optimized at PCM(dichloromethane)-B3LYPD3BJ/def2-TZVP level of theory and the Gibbs free energy determined by a frequency analysis using ORCA6.0.1[4]. Every stationary point was checked for the absence of imaginary frequencies for energy minima or for the presence of only a single imaginary frequency for transition states. Single point PCM(dichloromethane)-MP2/aug-cc-pVTZ calculations were performed on the B3LYPD3BJ optimized geometries and combined with the thermal correction from the frequency analysis to give the Gibbs free energies.

4) Supporting Figures

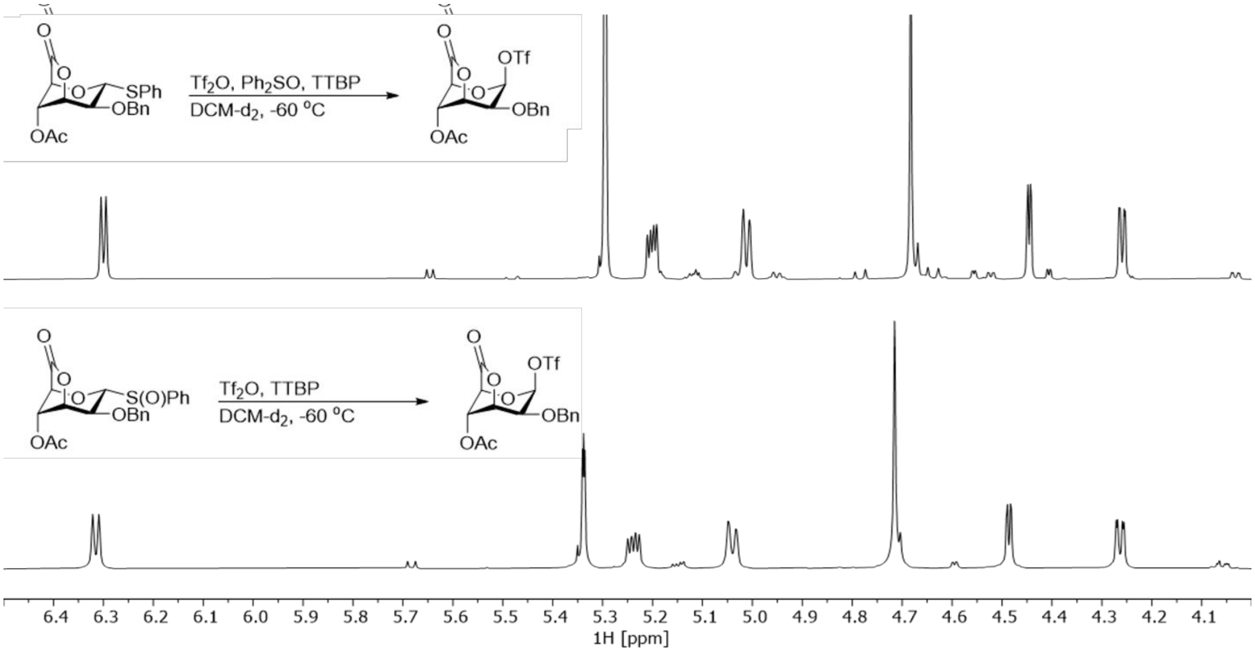

Figure S2: Comparison of sulfoxide activation with thioether activation. ^{1}H NMR spectrum of thioether **1** activated with Ph_2SO and Tf_2O in the presence of TTBP (top) and ^{1}H NMR spectrum of the sulfoxide derivative of **1** that was activated with Tf_2O in the presence of TTBP as previously reported (bottom).[5]

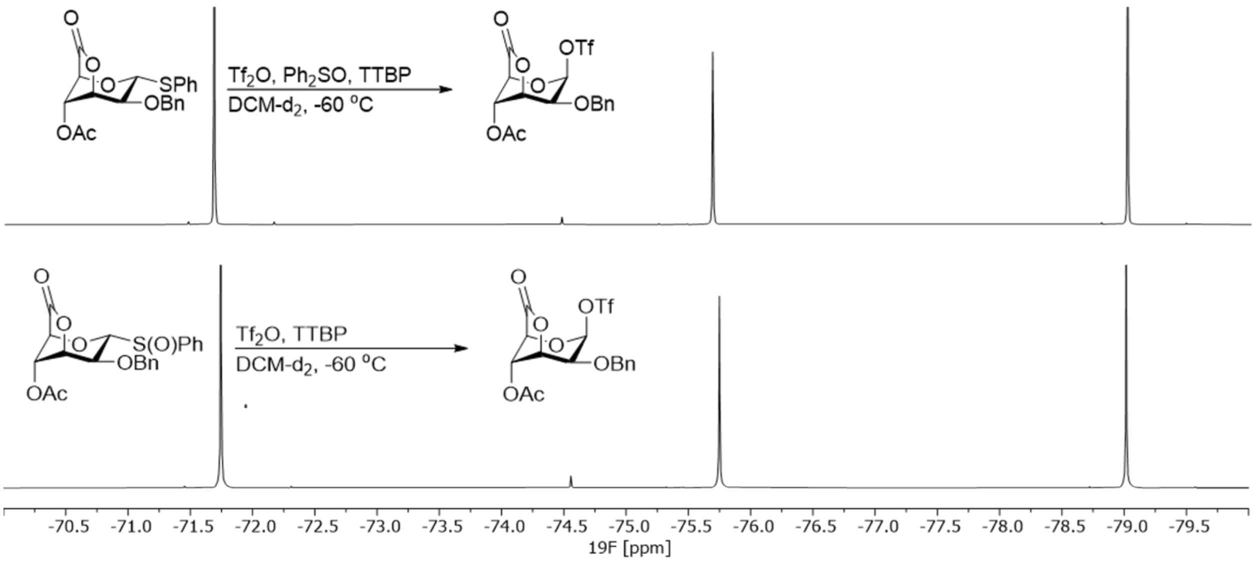

Figure S3: Comparison of thioether activation with sulfoxide activation. ^{19}F NMR spectrum of thioether **1** activated with Ph_2SO and Tf_2O in the presence of TTBP (top) and ^{19}F NMR spectrum of the sulfoxide derivative of **1** that was activated with Tf_2O in the presence of TTBP as previously reported (bottom).[5]

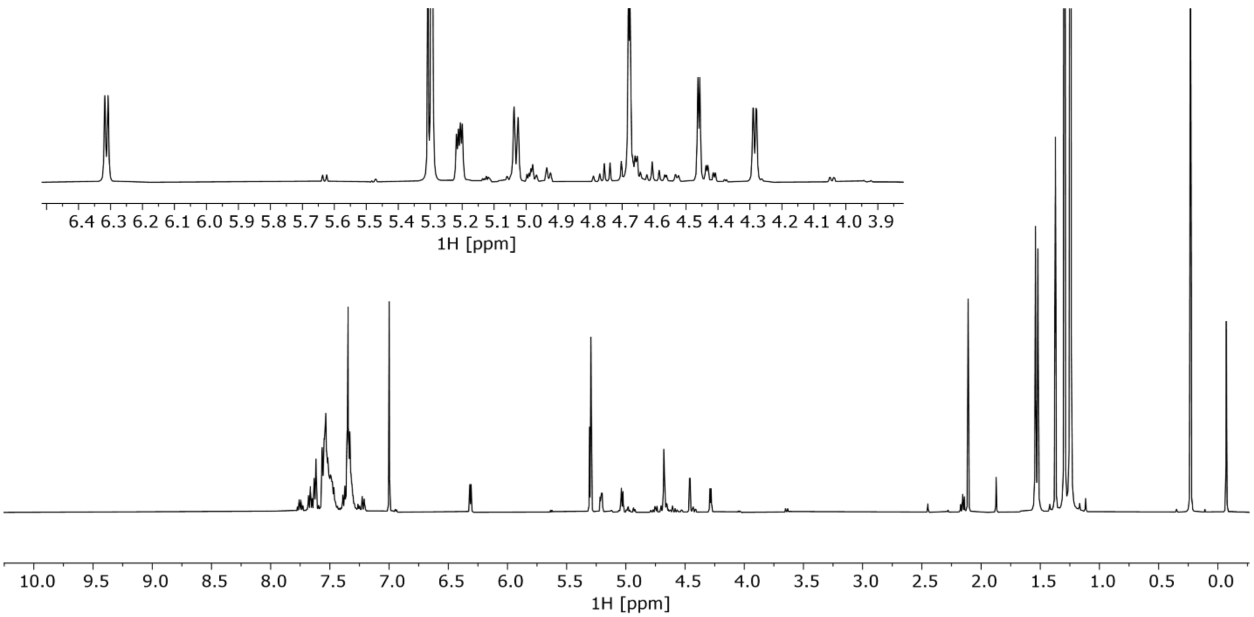

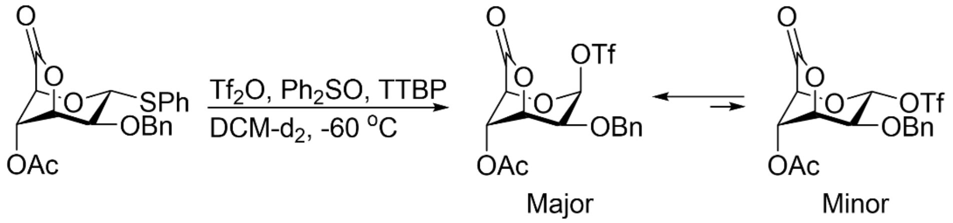

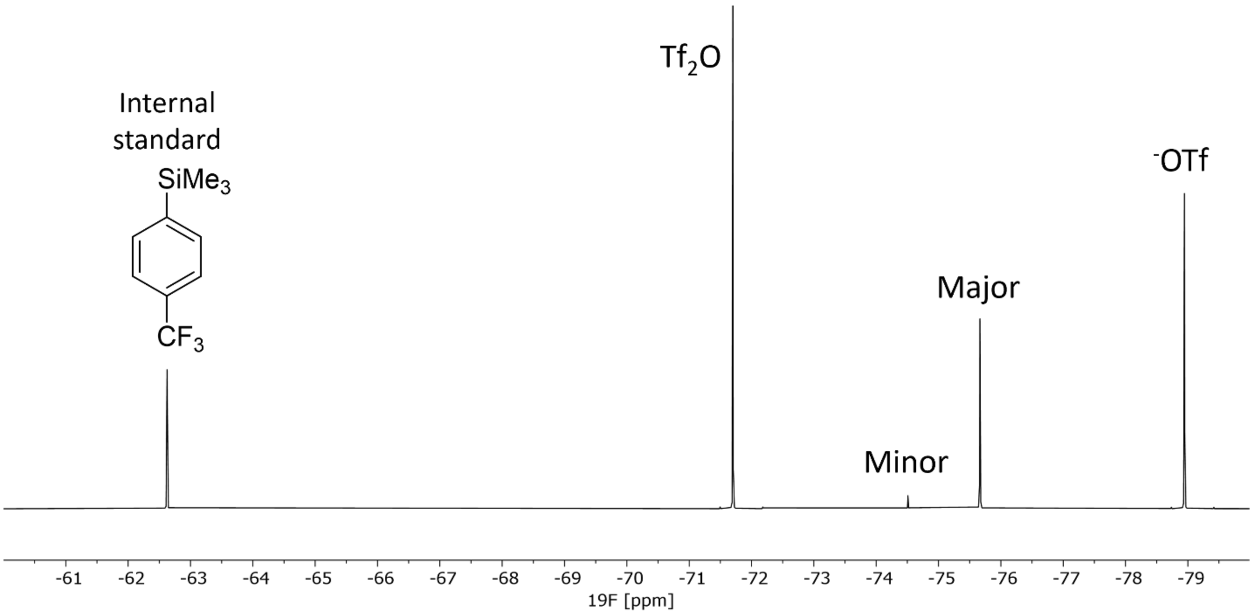

Figure S4: 6,3-Mannuronic acid lactone **1** activation. ^{1}H NMR spectrum (top) and ^{19}F NMR spectrum (bottom). An additional exchanging species (minor) can be found apart from the axial glycosyl triflate. This is possibly the equatorial glycosyl triflate.

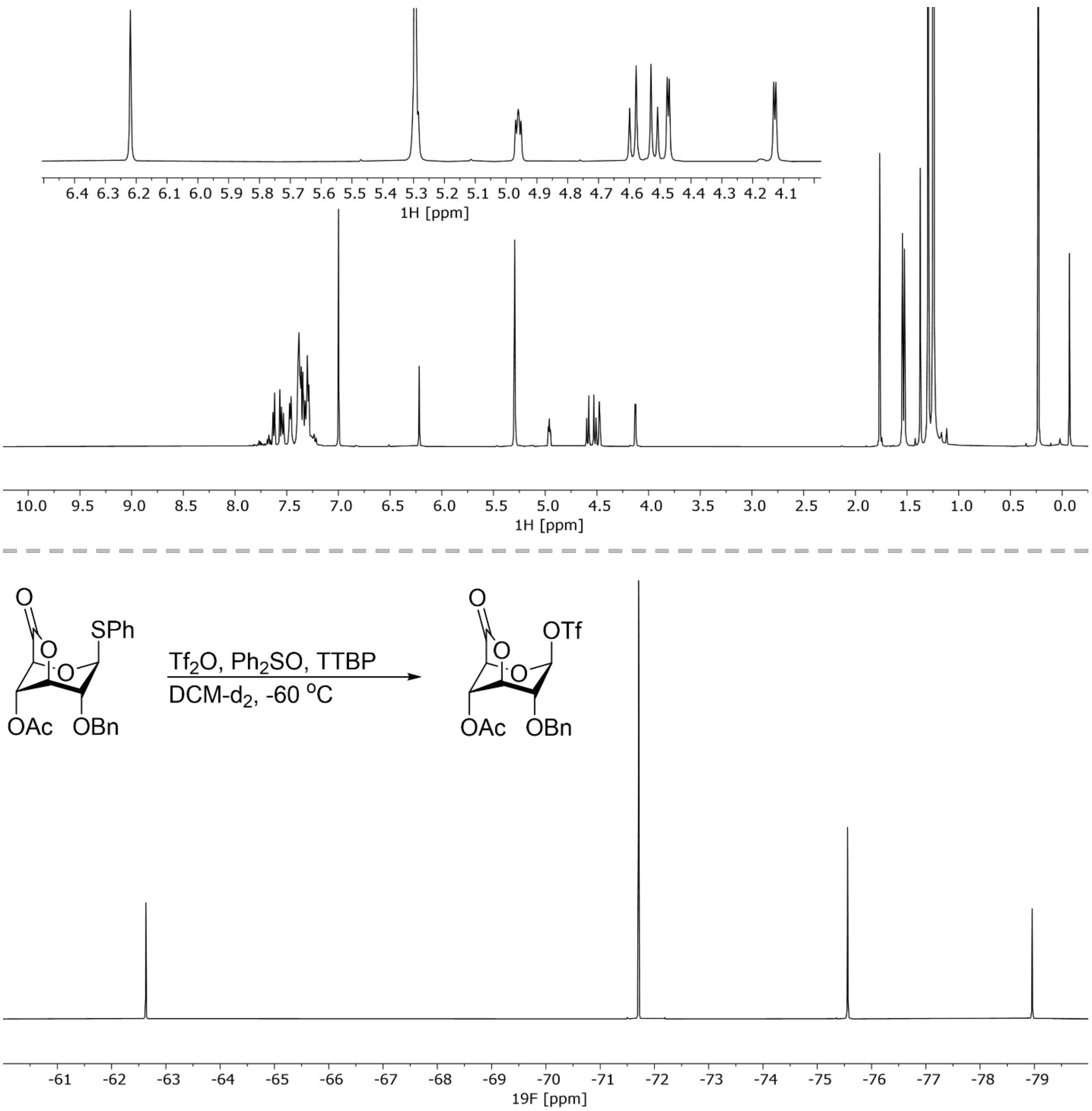

Figure S5: 6,3-Glucuronic acid lactone **2** activation. ^{1}H NMR spectrum (top) and ^{19}F NMR spectrum (bottom). No species can be found except for the axial glycosyl triflate.

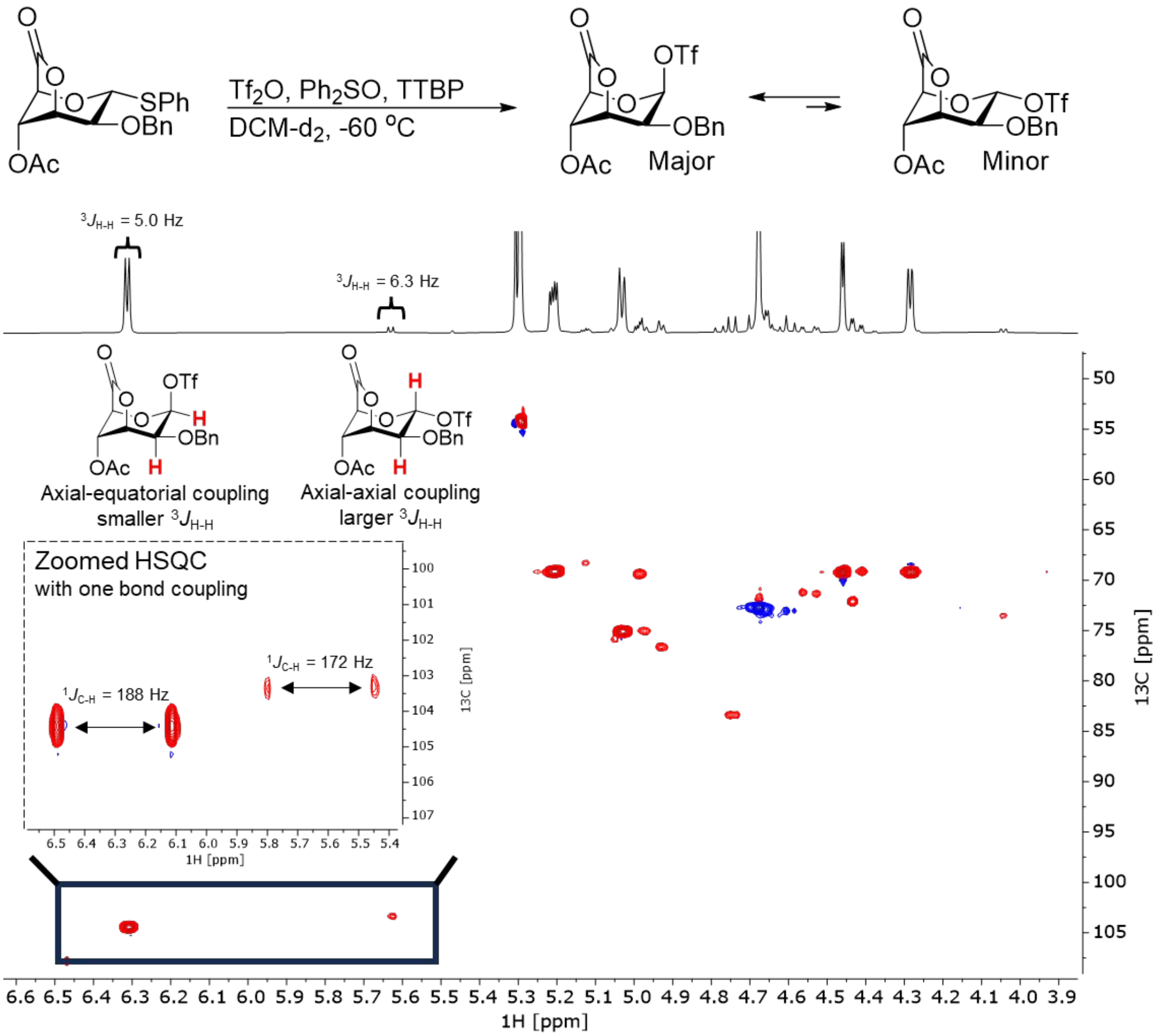

Figure S6: ^{13}C-coupled HSQC NMR spectrum of the activated 6,3-mannuronic acid lactone **1**. The major species (axial triflate $\mathbf{1}_{\mathbf{ax}}$) is in exchange with a minor species. The latter is determined to be an equatorial triflate ($\mathbf{1}_{\mathbf{eq}}$) based on its relatively large axial-axial $^{1}J_{H\text{-}H}$ and relatively small $^{1}J_{C\text{-}H}$.[6,7]

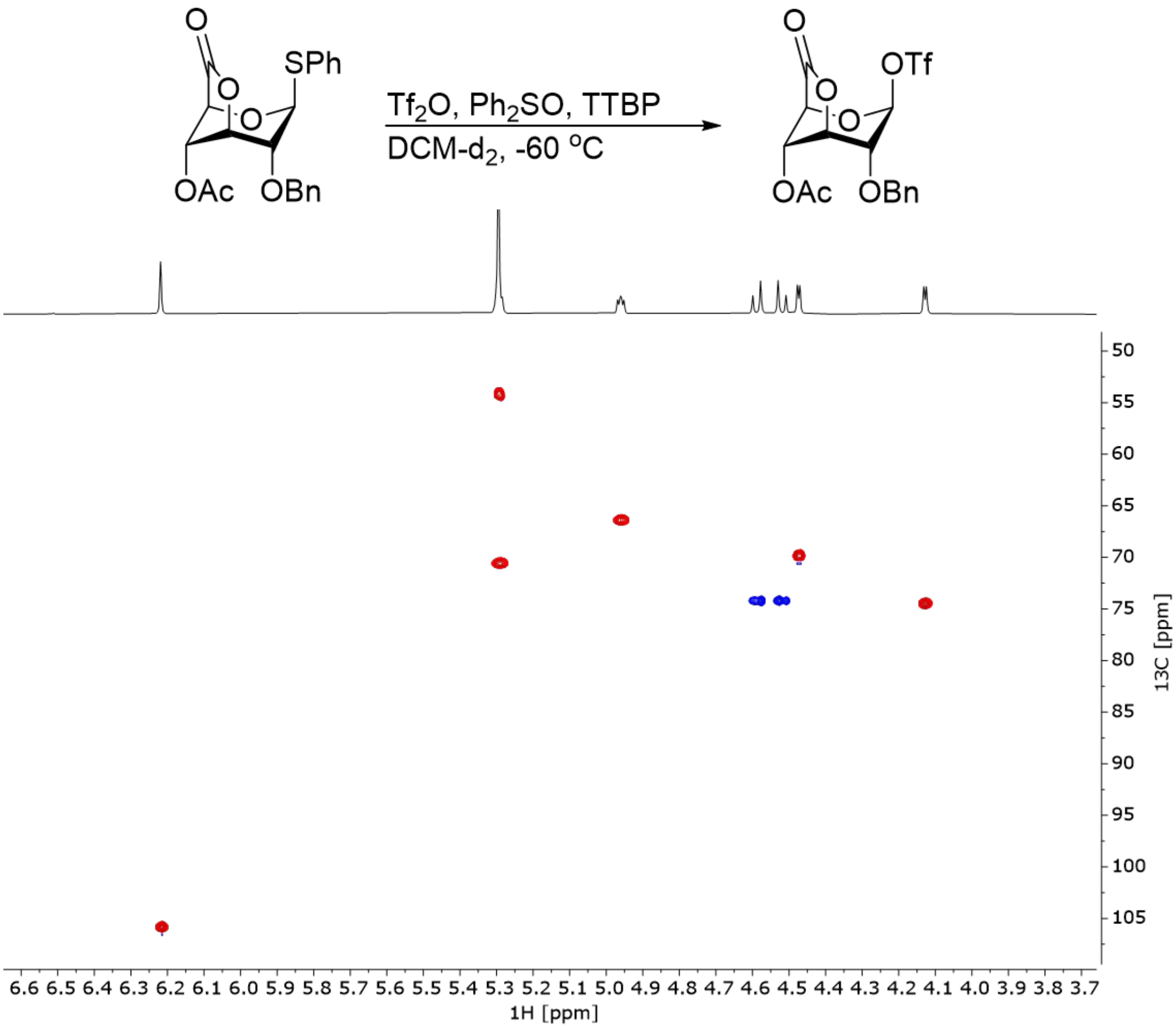

Figure S7: ^{13}C-coupled HSQC NMR spectrum of the activated 6,3-glucuronic acid lactone. No other species is found except for the axial triflate.

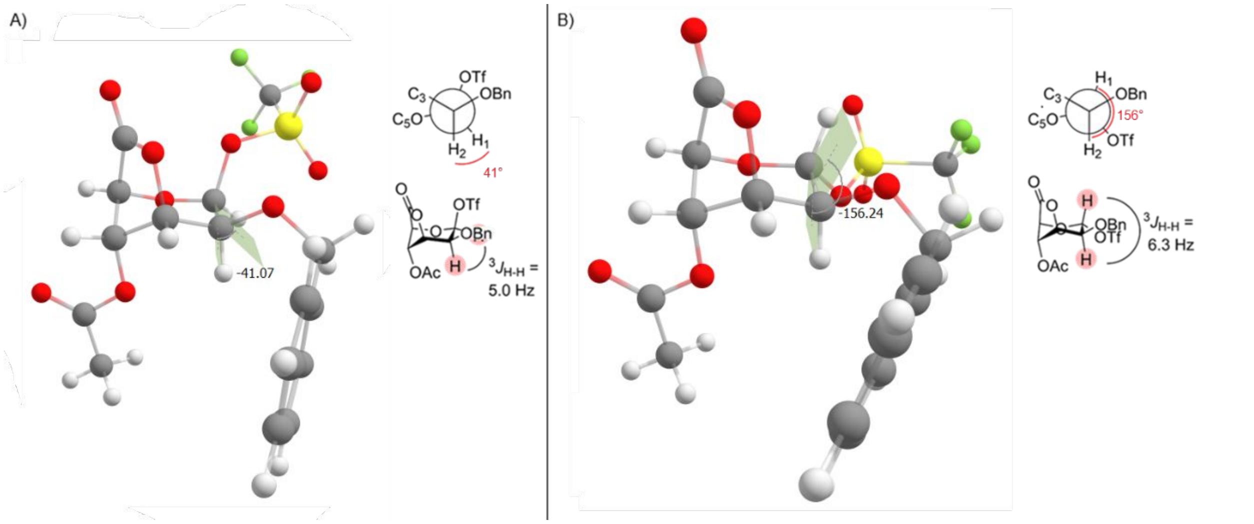

Figure S8: Lowest-energy conformations of the 6,3-mannuronic acid lactone triflate calculated with quantum chemistry. (A) Model of axial glycosyl triflate **1ax** (**1c**), which contains a *distorted* axial triflate with a dihedral angle of 41° between H-1 and H-2; (B) Model of equatorial glycosyl triflate **1eq** (**1d**), which contains a *distorted*-equatorial triflate with a dihedral angle of 156° between H-1 and H-2. When compared to the regular $^{1}C_4$-chair conformation and taking the Karplus relationship into account, these dihedral angles explain the relatively high and low $^{3}J_{H\text{-}H}$, respectively.

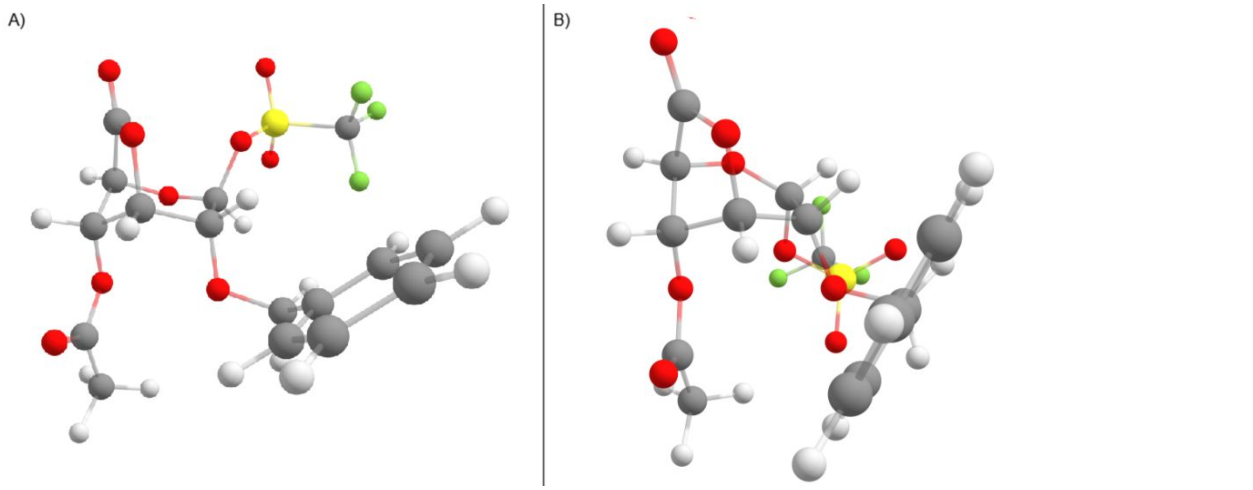

Figure S9: Lowest-energy conformations of the 6,3-glucuronic acid lactone triflate calculated with quantum chemistry. (A) Model of axial glycosyl triflate **2ax** (**2c**); (B) Model of equatorial glycosyl triflate **2eq** (**2d**), having a distorted triflate, making it *pseudo*-axial.

Glycosylation results and kinetic data

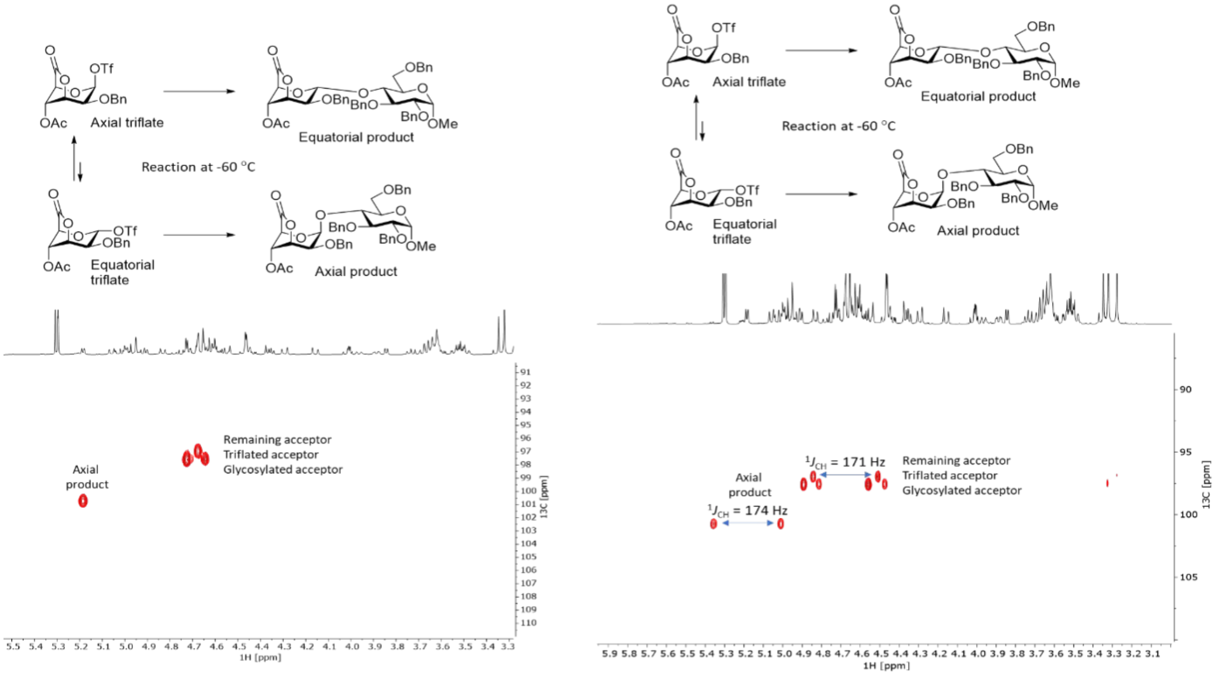

Figure S10: *In situ* glycosylation reaction outcome of the reaction with mannuronosyl lactone **1** and secondary acceptor **20**.

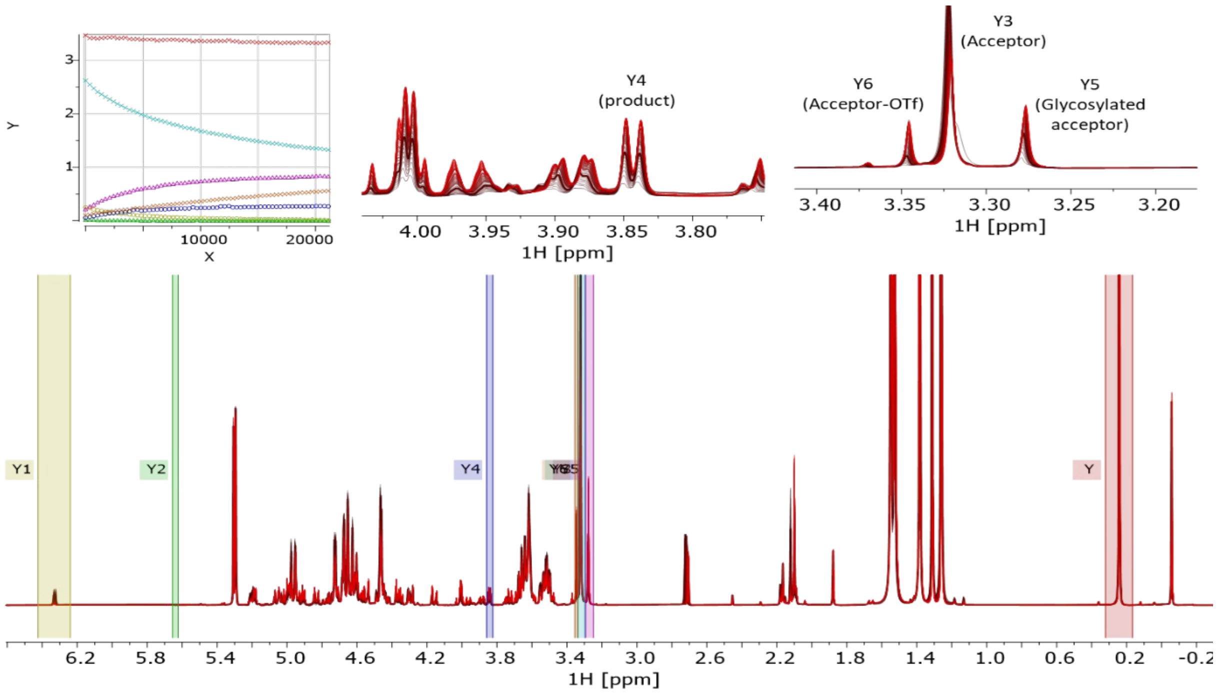

Figure S11: *In situ* glycosylation data-analysis of the reaction with mannuronosyl lactone **1** and secondary acceptor **20**.

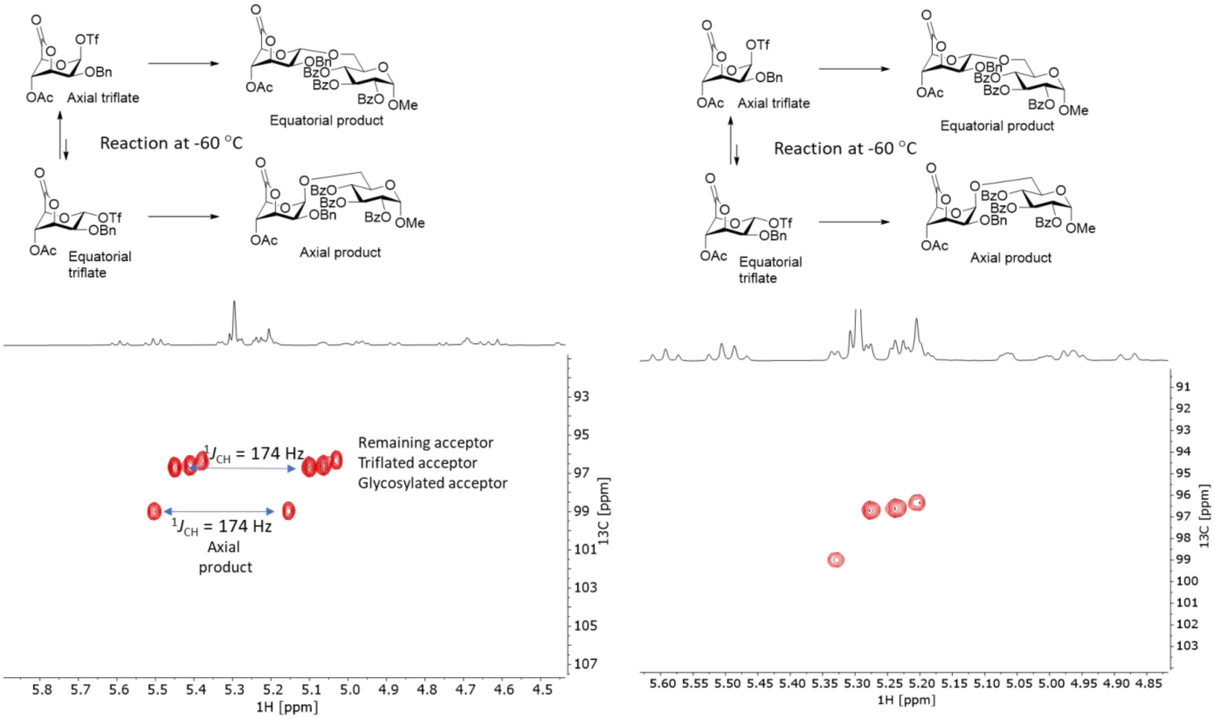

Figure S12: *In situ* glycosylation reaction outcome of the reaction with mannuronosyl lactone **1** and primary acceptor **21**.

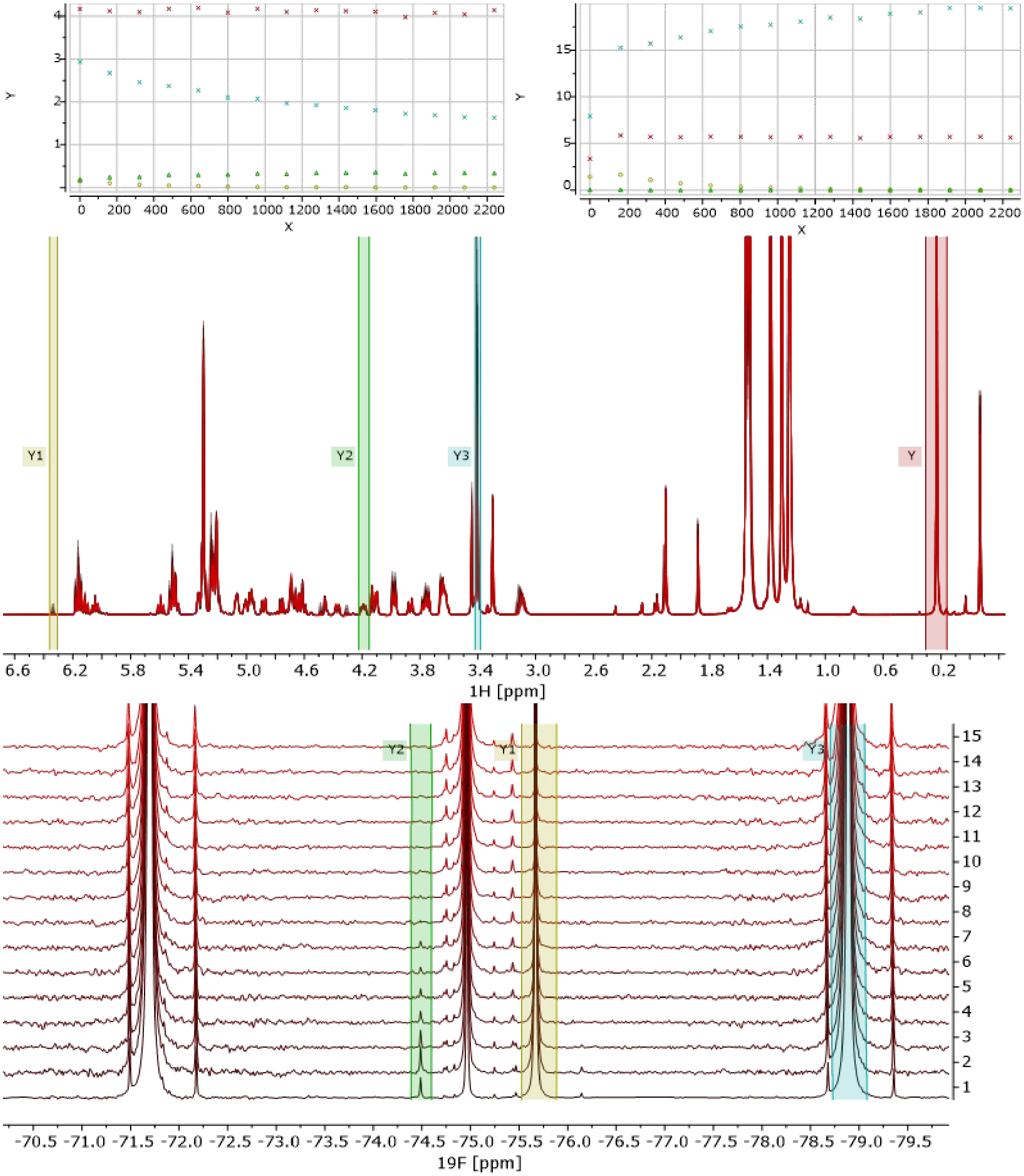

Figure S13: *In situ* glycosylation data-analysis of the reaction with mannuronosyl lactone **1** and primary acceptor **21**.

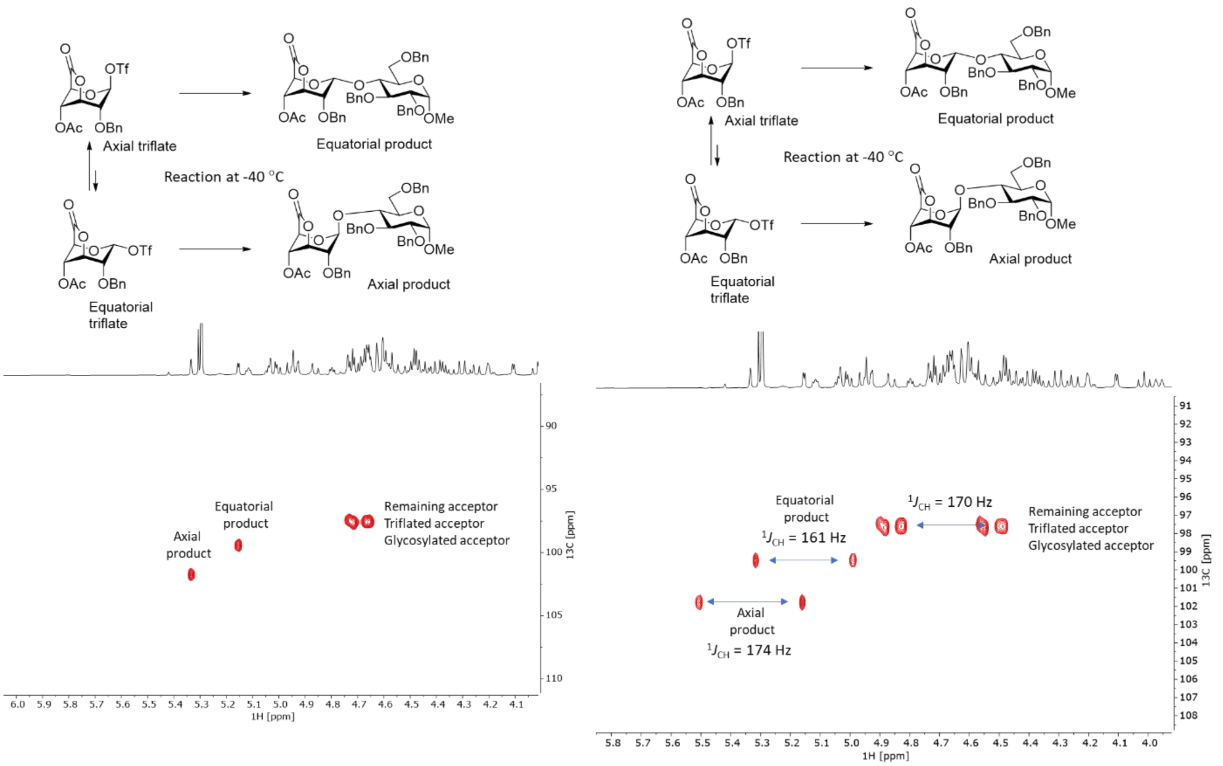

Figure S14: *In situ* glycosylation reaction outcome of the reaction with glucuronosyl lactone **2** and secondary acceptor **20**.

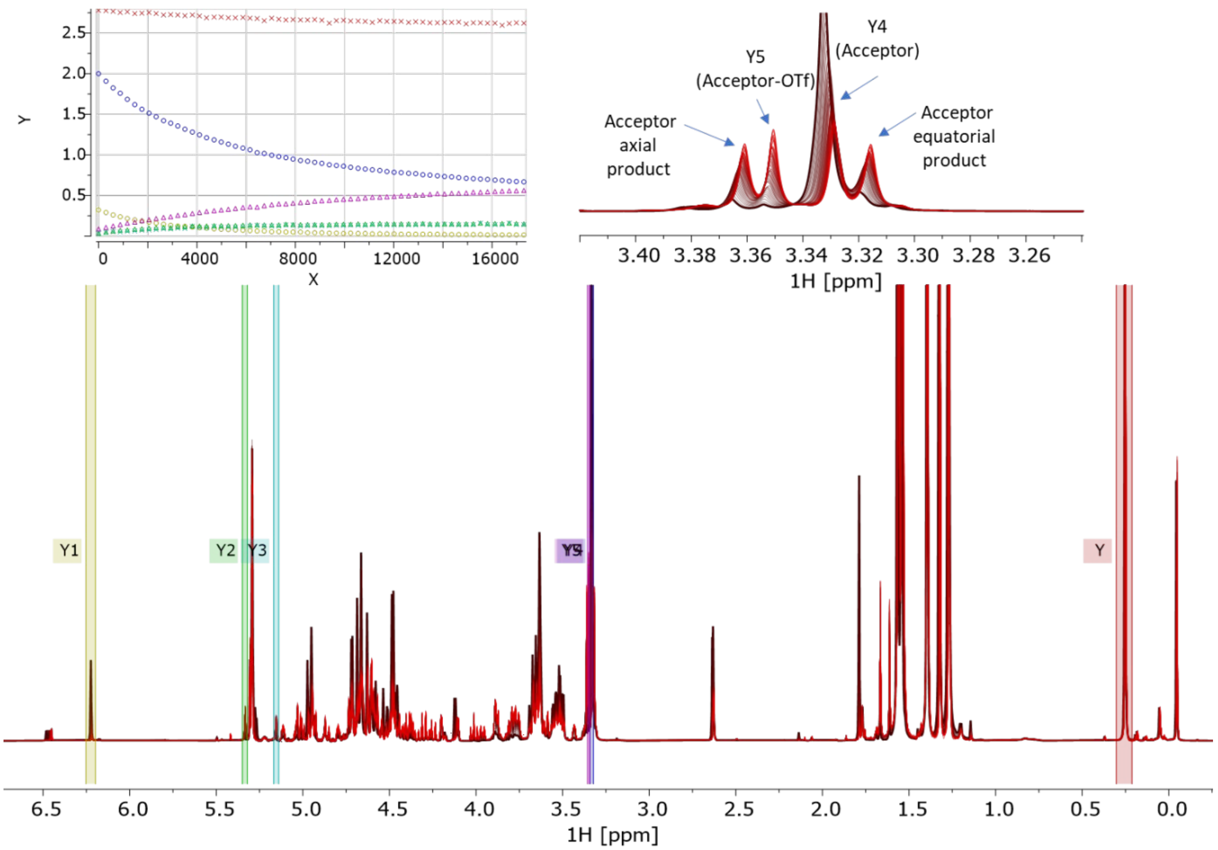

Figure S15: *In situ* glycosylation data-analysis of the reaction with glucuronosyl lactone **2** and secondary acceptor **20**.

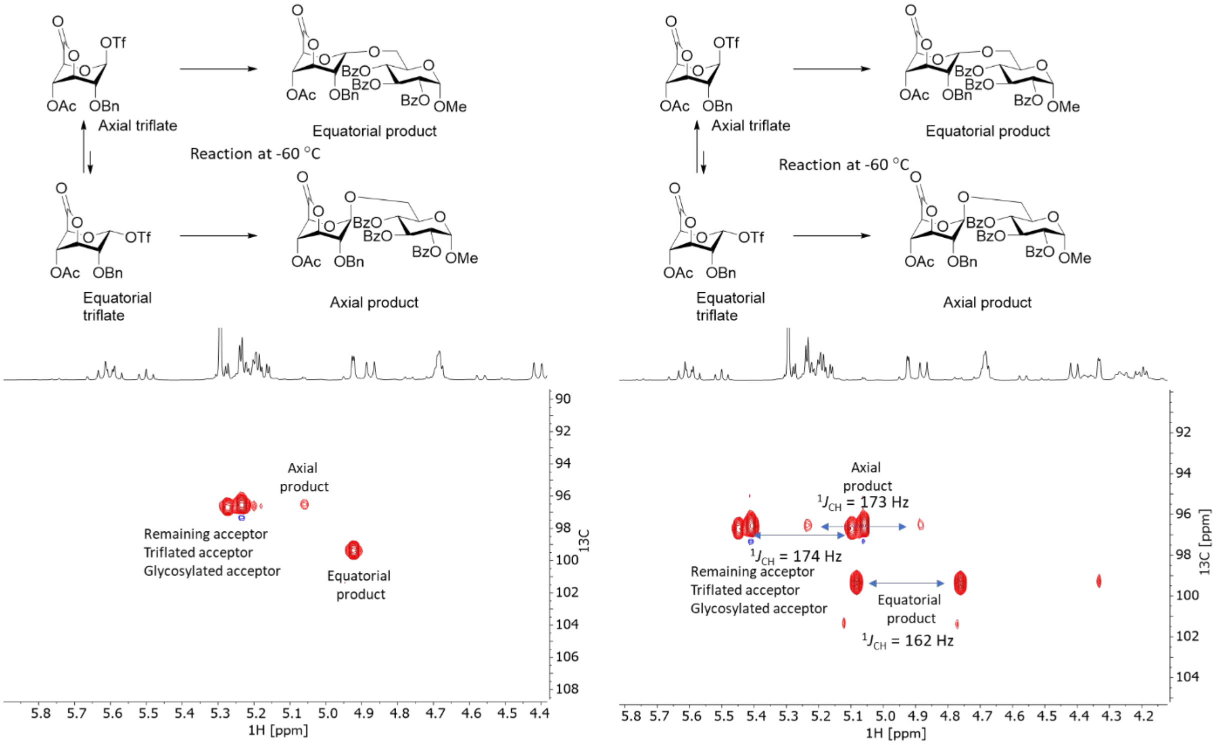

Figure S16: *In situ* glycosylation reaction outcome of the reaction with glucuronosyl lactone **2** and primary acceptor **21**.

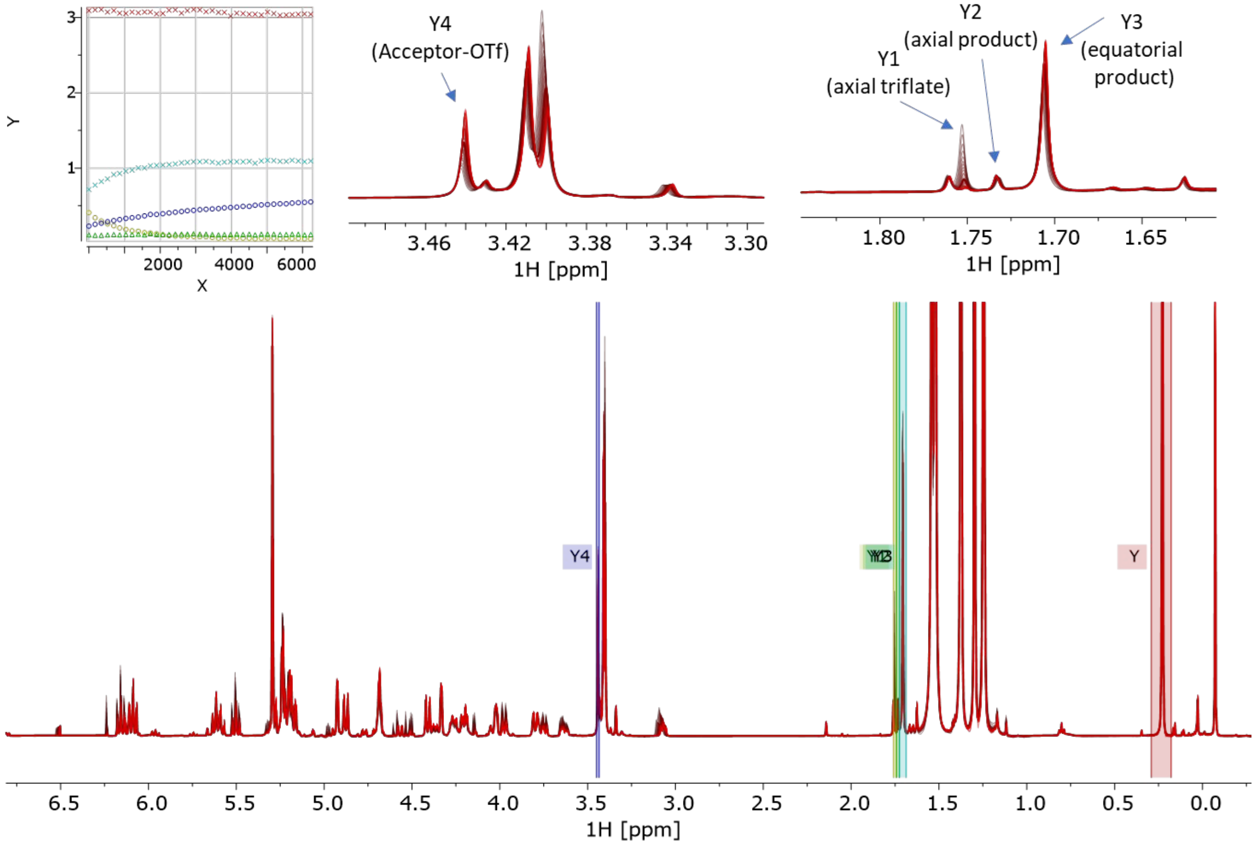

Figure S17: *In situ* glycosylation data-analysis of the reaction with glucuronosyl lactone **2** and primary acceptor **21**.

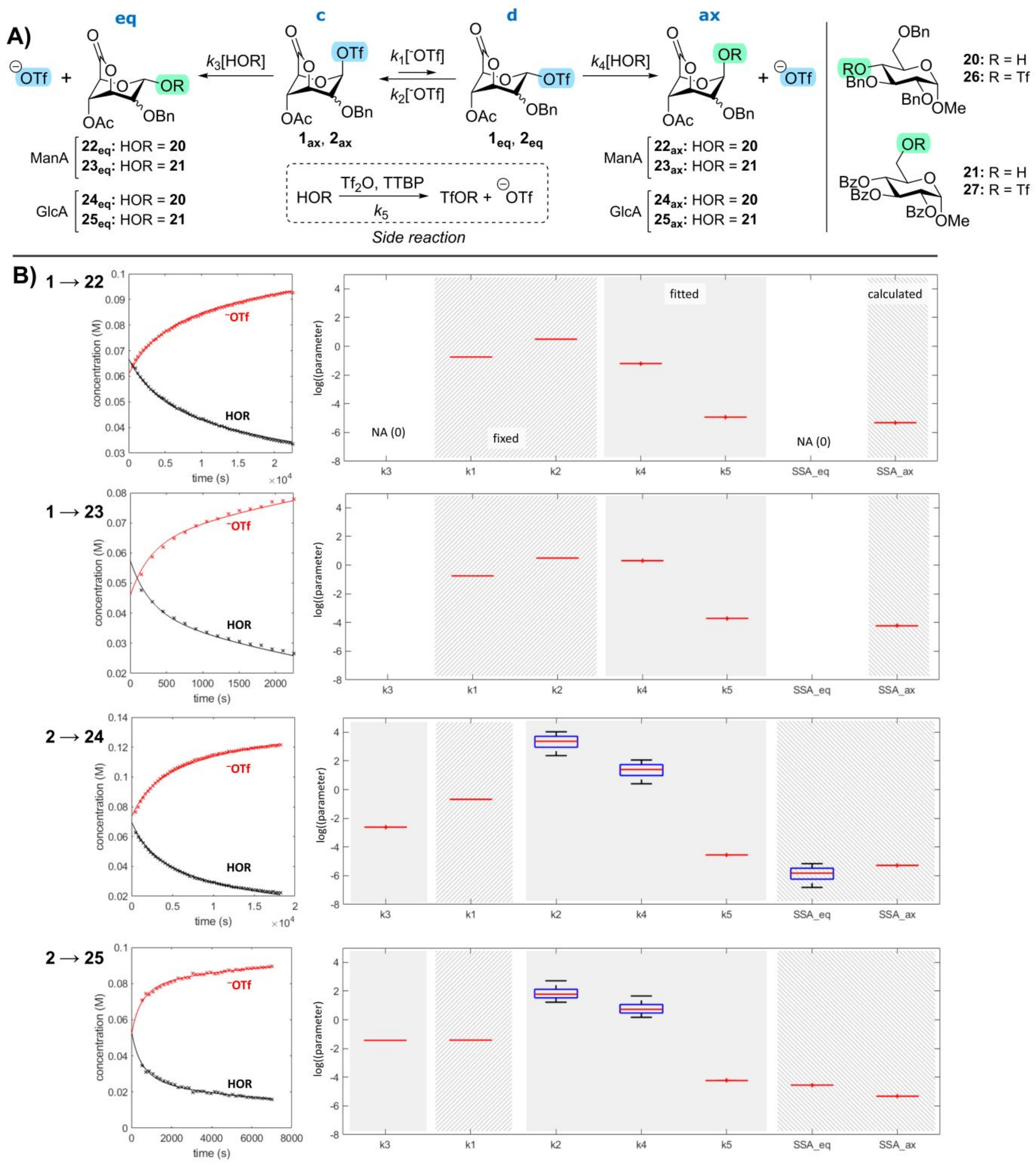

Figure S18: (A) General reaction scheme featuring nucleophilic displacement of the axial or equatorial glycosyl triflate intermediates. (B) Time-dependent concentrations of **HOR** and **⁻OTf**, corresponding the kinetic experiments shown in Figure 4B. The solid lines represent the best predictions of the kinetic model that has been fitted to the data. The right graphs show box whisker plots that feature the spread of the predicted parameters that produce fits with $\Sigma_{res} \leq 1.05 \cdot \min(\Sigma_{res})$. For the reactions (**1 → 22**) and (**1 → 23**), no equatorial product is formed and hence $k_3 = 0$ M^{-1} s^{-1}. The parameters with a grey background have been optimized in the fitting procedure; the parameters with the two different grey hatched backgrounds (///) and (\\\) were fixed in the fitting procedure and calculated based on the fitted parameters, respectively.

5) Synthesis materials & methods

General synthetic conditions

Synthetic product characterisations were recorded with a Bruker 500 MHz AVANCE III spectrometer or JEOL 500 ECZ-R spectrometer. The Bruker 500 MHz Avance III spectrometer is equipped with a Prodigy BB cryoprobe. The JEOL 500 ECZ-R spectrometers were equipped with either a SuperCOOL broadband probe, ROYAL broadband probe, or ROYAL HFX broadband probe. Chemical shifts are reported in parts per million (ppm) with tetramethylsilane (TMS) as the internal standard or solvent residual signals (SRP) if stated otherwise. ^{1}H-NMR spectroscopic data is presented as follows: chemical shift, multiplicity (s = singlet, d = doublet, t = triplet, dd = doublet of doublets, dt = doublet of triplets, m = multiplet and/or multiple resonances), coupling constant (*J*) in hertz (Hz), integration and assignments. All NMR signals were assigned based on ^{1}H-NMR, ^{13}C-NMR, COSY, HSQC, HMBC, TOCSY, NOESY and ROESY experiments. Mass spectra were recorded with a JEOL JMST100CS AccuTOF mass spectrometer. Automatic silica-flash column chromatography was done with a Biotage Isolera Spektra One, using pre-packed cartridges ultrapure irregular silica gel (Screening Devices, 40-63 µm, 60 Å). TLC analysis was conducted on Silica gel F254 (Merck KGaA) with detection by UV absorption (254 nm) where applicable and by dipping in a stain followed by heating. Stains used for TLC analysis were either 10% sulphuric acid in MeOH, cerium molybdate stain (0.03 M $(NH_4)_6Mo_7O_{24}\cdot4H_2O$; 6 mM $Ce(NH_4)_4(SO_4)_4\cdot2H_2O$; 1 M H_2SO_4 in H_2O) or potassium permanganate (0.06 M $KMnO_4$; 0.5 M, K_2CO_3; 0.02 M NaOH in H_2O). Reactions that used anhydrous solvents were performed under Schlenk conditions and were conducted under an argon atmosphere. Molecular Sieves (0.4 nm) were activated overnight by heating *in vacuo* at 150°C.

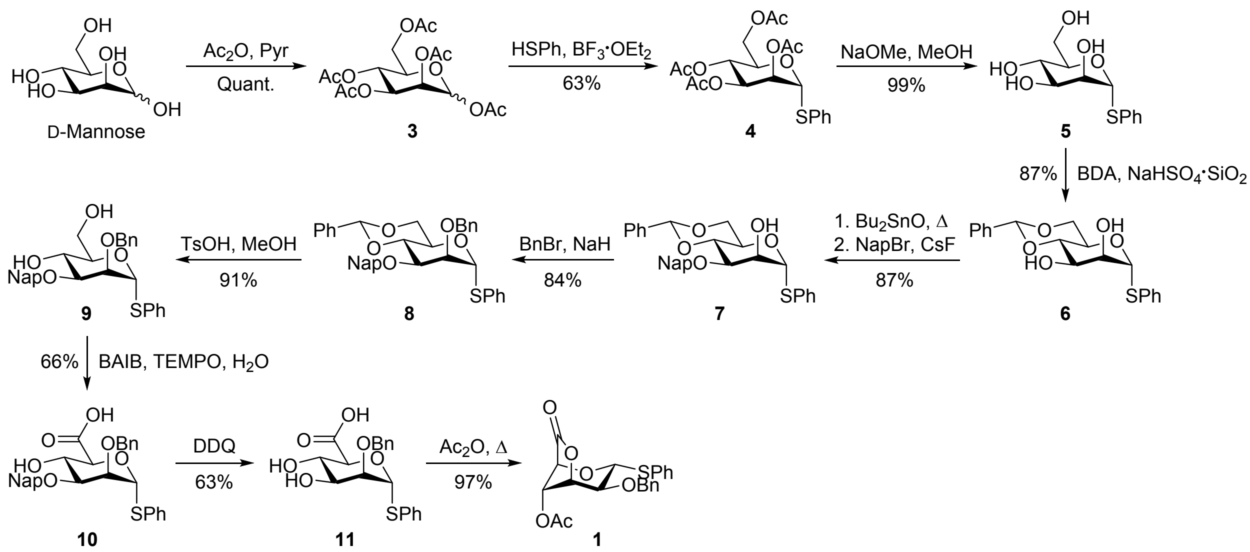

Scheme S2: Synthesis of 6,3-mannuronic acid lactone **1**.

1,2,3,4,6-Penta-*O*-acetyl-D-mannopyranoside (3)

Pyridine (300 ml) was added to a solution of D-mannose (25.0 g, 0.141 mol, 1.00 eq) in Ac_2O (150 ml, 1.59 mol, 11 eq) at 0°C. The reaction mixture was stirred at 0°C R.T. for 17 hrs. The solution was evaporated *in vacuo*. The residue was dissolved in EtOAc (150 ml). The solution was washed with 1.0 M aq. HCl (150 ml), H_2O (150 ml), aq. $NaHCO_3$ (sat.) (150 ml) and brine (150 ml), respectively. The organic layer was dried with $MgSO_4$, filtered and evaporated *in vacuo*, yielding monosaccharide **3** as a pale yellow oil (55.2 g, 0.141 mol, quantitative, α:β = 2.2:1).

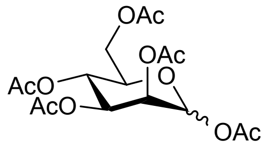

3

α-Anomer: **TLC**: (EtOAc/Hept, 60:40, v/v): R_f = 0.55; **^{1}H NMR** (500 MHz, $CDCl_3$): δ 6.08 (d, *J* = 2.0 Hz, 1H, ***H-1***), 5.37 – 5.33 (m, 2H, ***H-3***, ***H-4***), 5.26 (t, *J* = 2.3 Hz, 1H, ***H-2***), 4.34 – 4.24 (m, 1H, ***H-6$_a$***), 4.17 – 4.04 (m, 2H, ***H-5***, ***H-6$_b$***), 2.18 (s, 3H, C***H$_3$***, OAc), 2.18 (s, 3Hz, C***H$_3$***, OAc), 2.09 (s, 3H, C***H$_3$***, OAc), 2.06 (s, 3H, C***H$_3$***, OAc), 2.01 (s, 3H, C***H$_3$***, OAc); **^{13}C NMR** (126 MHz, $CDCl_3$): δ [170.56, 169.94, 169.71, 169.53, 168.06 (***C***=O**,** OAc)], 90.61(***C-1***), 70.62 (***C-5***), 68.78 (***C-3***), 68.36 (***C-2***), 65.54 (***C-4***), 62.10 (***C-6***), [20.82, 20.74, 20.68, 20.64, 20.61 (***C***H_3, OAc)];

β-Anomer: **TLC**: (EtOAc/Hept, 60:40, v/v): R_f = 0.55; **^{1}H NMR** (500 MHz, $CDCl_3$): δ 5.90 (d, *J* = 1.2 Hz, 1H, ***H-1***), 5.49 (dd, *J* = 3.4, 1.2 Hz, 1H, ***H-2***), 5.29 (t, J = 9.9 Hz, 1H, ***H-4***), 5.18 (dd, *J* = 10.0, 3.3 Hz, 1H, ***H-3***), 4.34 – 4.24 (m, 1H, ***H-6$_a$***), 4.17 – 4.04 (m, 1H, ***H-6$_b$***), 3.85 (ddd, *J* = 9.9, 5.3, 2.4 Hz, 1H, ***H-5***), 2.21 (s, 3H, C***H$_3$***, OAc), 2.10 (s, 3H, C***H$_3$***, OAc), 2.06 (s, 3H, C***H$_3$***, OAc), 2.04 (s, C***H$_3$***, OAc), 2.00 (s, C***H$_3$***, OAc); **^{13}C NMR** (126 MHz, $CDCl_3$): δ [170.59, 170.17, 169.75, 169.59, 168.35 (***C***=O, OAc)], 90.45 (***C-1***), 73.20 (***C-5***), 70.65 (***C-3***), 68.27 (***C-2***), 65.48 (***C-4***), 62.10 (***C-6***), [20.75, 20.72, 20.70, 20.65, 20.51 (***C***H$_3$, OAc)];
HR-ESI-TOF/Ms (m/z): $[M+Na]^+$ calcd for $C_{16}H_{22}O_{11}Na$, 413.10598; found, 413.10521.

Phenyl 2,3,4,6-tetra-O-acetyl-1-thio-α-D-mannopyranoside (4)

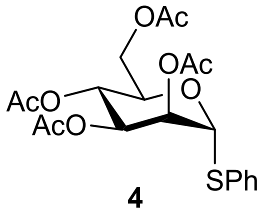

4

Monosaccharide **3** (34.6 g, 88.6 mmol, 1.00 eq) was dissolved in anh. DCM (180 ml). The solution was cooled down to 0°C. Thiophenol (11.8 ml, 115 mmol, 1.3 eq) was added. Next, $BF_3 \cdot OEt_2$ (38.9 ml, 310 mmol, 3.5 eq) was carefully added, after which the mixture was stirred at 0°C – R.T. for 48 hrs, turning purple over time. The solution was poured in ice-cold water (500 mL). The organic layer was extracted and subsequently washed with aq. NaHCO3 (sat.) (3x 100 ml) and brine (3x 100 ml), respectively. The organic layer was dried over $MgSO_4$, filtered and evaporated *in vacuo*. The residue was recrystallized in Et_2O/cyclohexane, yielding thioglycoside **4** as white crystals (39.0 g, 89.5 mmol, 63.3%).
TLC: (EtOAc/Hept, 60:40, v/v): R_f = 0.67; **^{1}H NMR** (500 MHz, $CDCl_3$): δ 7.51 – 7.47 (m, 2H, 2x Ar***H***, SPh), 7.34 – 7.29 (m, 2H, 3x Ar***H***, SPh), 5.53 – 5.47 (m, 2H, ***H-1***, ***H-2***), 5.38 – 5.26 (m, 2H, ***H-3***, ***H-4***), 4.60 – 4.51 (m, 1H, ***H-5***), 4.31 (dd, *J* = 12.2, 5.9 Hz, 1H, ***H-6$_a$***), 4.11 (dd, *J* = 12.2, 2.4 Hz, 1H, ***H-6$_b$***), 2.15 (s, 3H, C***H$_3$***, OAc), 2.08 (s, 3H, C***H$_3$***, OAc), 2.05 (s, 3H, C***H$_3$***, OAc), 2.02 (s, 3H, C***H$_3$***, OAc); **^{13}C NMR** (126 MHz, $CDCl_3$): δ [170.56, 169.93, 169.84, 169.76 (***C***=O, OAc)], 132.63 (Ar***C***S, SPh), [132.07, 129.19, 128.13 (SPh)], 85.70 (***C-1***), 70.91 (***C-2***), 69.52 (***C-5***), 69.38 (***C-3***), 66.38 (***C-4***), 62.45 (***C-6***), [20.89, 20.72, 20.70, 20.65 (***C***H$_3$, OAc)]; **HR-ESI-TOF/Ms (m/z):** $[M+Na]^+$ calcd for $C_{20}H_{24}O_9SNa$, 463.10387; found, 463.10232.

Phenyl 1-thio-α-D-mannopyranoside (5)

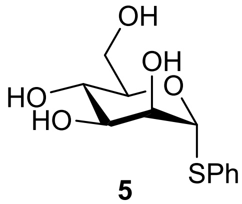

5

Thioglycoside **4** (39.0 g, 88.5 mmol, 1.00 eq) was dissolved in anh. MeOH (443 ml). 5.4 M NaOMe in MeOH (6.56 mL, 35.4 mmol, 0.40 eq) was added. The mixture was stirred at R.T. for 21 hrs. Dowex® ion exchange(H+) resin (2.0 g) was added, after which the mixture was stirred for an additional 20 min. The suspension was filtered and evaporated *in vacuo*, yielding monosaccharide **5** as a white foam (23.9 g, 87.8 mmol, 99.1%).
TLC: (MeOH/DCM, 10:90, v/v): R_f = 0.10; **^{1}H NMR** (500 MHz, DMSO-d$_6$): δ 7.53 –7.26 (m, 5H, Ar***H***, SPh), 5.34 (d, *J* = 1.5 Hz, 1H, ***H-1***), 5.13 (d, *J* = 4.2 Hz, 1H, 2-O***H***), 4.88 (d, *J* = 5.2 Hz, 1H, 4-O***H***), 4.79 (d, *J* = 5.2 Hz, 1H, 3-O***H***), 4.50 (t, *J* = 6.0 Hz, 1H, 6-O***H***), 3.91 – 3.85 (ddd, *J* = 4.4, 2.9, 1.6 Hz, 1H, ***H-2***), 3.77 (ddd, *J* = 8.6, 6.1, 2.1 Hz, 1H, ***H-5***), 3.66 (ddd, *J* = 11.8, 5.7, 2.2 Hz, 1H, ***H-6$_a$***), 3.55 – 3.42 (m, 3H, ***H-3***, ***H-4***, ***H-6$_b$***); **^{13}C NMR** (126 MHz, DMSO-d$_6$; solvent peak ref'd to 39.52): δ 134.91 (Ar***C***S, SPh), [131.04, 129.02, 127.05 (SPh)], 88.87 (***C-1***), 75.39 (***C-5***), 71.92 (***C-2***), 71.53 (***C-3***), 67.00 (***C-4***), 60.89 (***C-6***); **HR-ESI-TOF/Ms (m/z):** $[M+Na]^+$ calcd for $C_{12}H_{16}O_5SNa$, 295.06161; found, 295.06176.

Phenyl 4,6-*O*-benzylidene-1-thio-α-D-mannopyranoside (6)

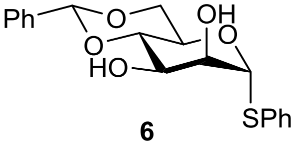

6

Thioglycoside **5** (23.7 g, 87.0 mmol, 1.0 eq) was suspended in anh. ACN (396 ml). Benzaldehyde dimethyl acetal (14.4 ml, 95.7 mmol, 1.1 eq) and $NaHSO_4 \cdot SiO_2$ (7.47 g, 42% Wt, 17.4 mmol, 0.20 eq) were added. The mixture was stirred at R.T. for 45 min, . The reaction mixture was neutralized with TEA. The suspension was filtered and washed with ACN. The residue was partially dissolved in DCM (2.0 L) and filtered. The remaining residue was dissolved in hot ethanol and filtered. The combined filtrates were evaporated *in vacuo*, yielding monosaccharide **6** as a white solid (27.3 g, 75.7 mmol, 87.0%).
TLC: (EtOAc/Hept, 50:50 v/v): R_f = 0.35; **^{1}H NMR** (500 MHz, DMSO-d$_6$): δ 7.51 – 7.44 (m, 4H, 2x Ar***H***, SPh; 2x Ar***H***, $PhCHO_2$), 7.41 – 7.29 (m, 6H, 3x Ar***H***, SPh; 3x Ar***H***, $PhCHO_2$), 5.63 (s, 1H, PhC***H***O$_2$), 5.54 (d, *J* = 4.1 Hz, 1H, 2-O***H***), 5.46 (d, *J* = 1.4 Hz, 1H, ***H-1***), 5.21 (d, *J* = 6.0 Hz, 1H, 3-O***H***), 4.06 (m, 2H, ***H-5***, ***H-6$_a$***), 4.02 – 3.99 (m, 1H, ***H***-2), 3.95 (t, *J* = 9.3 Hz, 1H, ***H-4***), 3.82 – 3.71 (m, 2H, ***H***-3, ***H-6$_b$***); **^{13}C NMR** (126 MHz, DMSO-d$_6$; solvent peak ref'd to 39.52): δ 137.83 (Ar***C***CHO$_2$), 133.65 (Ar***C***S, SPh), 131.30 (SPh), [129.26, 128.85, 128.01 (SPh; $PhCHO_2$)], 127.45 (SPh), 126.38 ($PhCHO_2$),

101.17 (Ph***C***HO_2), 89.26 (***C-1***), 78.45 (***C-4***), 72.40 (***C-2***), 68.05 (***C-3***), 67.58 (***C-6***), 65.28 (***C-5***); **HR-ESI-TOF/Ms (m/z):** [M+Na]+ calcd for $C_{19}H_{20}O_5SNa$, 383.09291; found, 383.09402.

Phenyl 4,6-*O*-benzylidene-3-*O*-(naphthalene-2-ylmethyl)-1-thio-α-D-mannopyranoside (7)

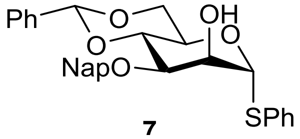

7

Thioglycoside **6** (25.4 g, 70.5 mmol, 1.00 eq) was dissolved in toluene (2.0 L). Bu_2SnO (26.3 g, 106 mmol, 1.5 eq) was added, after which the mixture was refluxed for 4 hours using a dean stark apparatus. The clear solution was concentrated *in vacuo* and resuspended in anh. DMF (250 ml). CsF (21.4 g, 141 mmol, 2.0 eq) and 2-bromomethylnaphthalene (23.4 g, 106 mmol, 1.5 eq) were added. The mixture was heated to 95°C and stirred for 18 hrs, after which the mixture was evaporated *in vacuo*. The residue was purified using silica-flash column chromatography (20 - 50% EtOAc in Hept), yielding monosaccharide **7** (30.5 g, 60.9 mmol, 86.5%).

TLC: (EtOAc/Hept, 25:75, v/v): R_f = 0.66; **^{1}H NMR** (500 MHz, $CDCl_3$): δ 7.83 – 7.80 (m, 3H, 3x Ar***H***, ONap), 7.77 – 7.72 (m, 1H, Ar***H***, ONap), 7.55 – 7.36 (m, 10H, 3x Ar***H***, ONap; 2x ArH, SPh; 5x Ar***H***, PhCHO_2), 7.33 – 7.24 (m, 3H, 3x Ar***H***, SPh); 5.64 (s, 1H, PhC***H***O_2), 5.60 (d, *J* = 1.2 Hz, 1H, ***H-1***), 5.03 (d, *J* = 12.1 Hz, 1H, ArC$\boldsymbol{H_a}$$H_b$, ONap), 4.92 (d, *J* = 12.1 Hz, 1H, ArC$H_a$$\boldsymbol{H_b}$, ONap), 4.37 – 4.30 (m, 2H, ***H-2***, ***H-5***), 4.25 – 4.18 (m, 2H, ***H-6$_a$***, ***H-4***), 4.02 (dd, *J* = 9.5, 3.4 Hz, 1H, ***H-3***), 3.86 (t, *J* = 10.3 Hz, 1H, ***H-6$_b$***); **^{13}C NMR** (126 MHz, $CDCl_3$): δ 137.48 (Ar***C***CHO_2), 135.14 (Ar***C***CH_2, ONap) , 133.27 (Ar***C***S, SPh), 133.25 (Ar***C***C_2, ONap), 133.12 (Ar***C***C_2, ONap), 131.78 (SPh), 129.16 (SPh), [129.04, 128.38, 128.30, 127.99, 127.73 (SPh; PhCHO_2; ONap)] , 126.71 (ONap), [126.24, 126.17, 126.11 (PhCHO_2; ONap)], 125.67 (ONap), 101.76 (Ph***C***HO_2), 87.81 (***C-1***), 78.99 (***C-4***), 75.71 (***C-3***), 73.13 (Ar***C***H_2, ONap), 71.39 (***C-2***), 68.56 (***C-6***), 64.63 (***C-5***); **HR-ESI-TOF/Ms (m/z):** $[M+Na]^+$ calcd for $C_{30}H_{28}O_5SNa$, 523.15551; found, 523.15561.

Phenyl 2-*O*-benzyl-4,6-*O*-benzylidene-3-*O*-(naphthalene-2-ylmethyl)-1-thio-α-D-mannopyranoside (8)

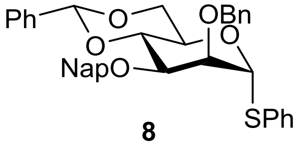

8

Thioglycoside **7** (27.9 g, 57.3, 1.0 eq) was dissolved in anh. DMF (441 ml). The solution was cooled down to 0°C. NaH (4.59 g, 60% Wt, 115 mmol, 2.0 eq) was added. The mixture was stirred for 45 min, after which BnBr (8.18 ml, 68.8 mmol, 1.2 eq) was carefully added. The mixture was stirred at 0°C – R.T. for 48 hrs, turning orange over time. The mixture was quenched with aq. NH_4Cl and concentrated *in vacuo*. The residue was resuspended in DCM (400 mL) and washed with H_2O (400 ml) and brine (400 ml), respectively. The organic layer was dried over $MgSO_4$, filtered and evaporated *in vacuo*. The residue was purified using silica-flash column chromatography (0 – 30% EtOAc in Hept), yielding monosaccharide **8** (27.9 g, 48.4 mmol, 84.4%) as a white foam.

TLC: (EtOAc:Hept, 25:75 v/v): R_f = 0.76; **^{1}H NMR** (500 MHz, DMSO-d_6) δ 7.94 – 7.84 (m, 3H, 3x Ar***H***, ONap), 7.77 – 7.71 (m, 1H, Ar***H***, ONap), 7.53 – 7.28 (m, 18H, 3x Ar***H***, ONap; 5x ArH, SPh; 5x Ar***H***, OBn; 5x ArH, PhCHO_2), 5.80 – 5.76 (m, 2H, ***H-1***, PhC***H***O_2), 4.84 – 4.77 (m, 3H, ArC$\boldsymbol{H_2}$, ONap; PhC$\boldsymbol{H_a}$$H_b$, OBn), 4.69 (d, *J* = 11.8 Hz, 1H, PhC$H_a$$\boldsymbol{H_b}$, OBn), 4.28 – 4.19 (m, 2H, ***H-2***, ***H-4***), 4.16 – 4.05 (m, 2H, ***H-5***, ***H-6$_a$***), 3.91 (dd, *J* = 10.0, 3.2 Hz, 1H, ***H-3***), 3.85 (t, *J* = 10.0 Hz, 1H, ***H-6$_b$***); **^{13}C NMR** (126 MHz, DMSO-d_6) δ 138.17 (Ar***C***CH_2, OBn), 137.76 (Ar***C***CHO_2), 135.06 (Ar***C***CH_2, ONap), 133.03 (Ar***C***S, SPh), 132.77 (Ar***C***C_2, ONap), 132.43 (Ar***C***C_2, ONap), 131.47 (SPh), [129.24, 128.84, 128.23, 128.12, 127.94, 127.79, 127.64, 127.62, 127.59 (SPh; ONap; OBn; PhCHO_2)], 126.16 (PhCHO_2), [125.87, 125.75 , 125.72 (ONap)], 100.73 (Ph***C***HO_2), 85.75 (***C-1***), 77.78 (***C-4***), 77.12 (***C-2***), 75.60 (***C-3***), 72.03 (Ph***C***H_2, OBn), 70.87 (Ph***C***H_2, ONap), 67.45 (***C-6***), 65.28 (***C-5***); **HR-ESI-TOF/Ms (m/z):** $[M+Na]^+$ calcd for $C_{37}H_{34}O_5S$, 613.20246; found, 613.20264.

Phenyl 2-*O*-benzyl-3-*O*-(naphthalene-2-ylmethyl)-1-thio-α-D-mannopyranoside (9)

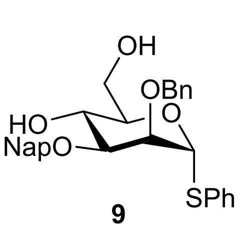

9

Thioglycoside **8** (2.3 g, 3.8 mmol, 1.0 eq) and TsOH·H_2O (76 mg, 0.39 mmol, 0.10 eq) were suspended in MeOH (38 mL). The mixture was stirred at R.T. for 20 hrs. The solution was quenched with TEA (0.50 mL) and evaporated *in vacuo*. The residue was dissolved in EtOAc (50 mL) and washed with H_2O (30 mL), aq. $NaHCO_3$ (sat.) (30 mL) and brine (50 mL), respectively. The organic layer was dried with MgSO4, filtered and evaporated *in vacuo*. The residue was purified using silica-flash column chromatography (10 – 60% EtOAc in Hept), yielding monosaccharide **9** as a white foam (1.8 g, 3.5 mmol, 91%).

<u>TLC</u>: (EtOAc:Hept, 60:40 v/v): R_f = 0.17; **<u>^{1}H NMR</u>** (500 MHz, $CDCl_3$): δ 7.86 – 7.79 (m, 3H, 3x Ar***H***, ONap), 7.77 (s, 1H, Ar***H***, ONap), 7.51 – 7.46 (m, 2H, 2x Ar***H***, ONap), 7.43 (dd, *J* = 8.4, 1.7 Hz, 1H, Ar***H***, ONap), 7.41 – 7.38 (m, 2H, 2x Ar***H***, SPh), 7.33 – 7.24 (m, 8H, 3x Ar***H***, SPh; 5x Ar***H***, OBn), 5.55 (d, *J* = 1.6 Hz, 1H, ***H-1***), 4.72 (d, *J* = 11.9 Hz, 1H, ArC$\boldsymbol{H_a}$$H_b$, 3-ONap), 4.69 – 4.62 (m, 2H, PhC$\boldsymbol{H_a}$$H_b$, 2-OBn; ArC$H_a$$\boldsymbol{H_b}$, ONap), 4.56 (d, *J* = 12.2 Hz, 1H, PhC$H_a$$\boldsymbol{H_b}$, OBn), 4.18 – 4.09 (m, 2H, ***H-4***, ***H-5***), 4.03 (dd, *J* = 3.1, 1.6 Hz, 1H, ***H-2***), 3.89 – 3.80 (m, 2H, $\boldsymbol{H\text{-}6_a}$, $\boldsymbol{H\text{-}6_b}$), 3.76 (dd, *J* = 9.0, 3.0 Hz, 1H, ***H-3***), 2.68 (d, *J* = 2.4 Hz, 1H, 4-O***H***), 2.08 (t, *J* = 6.4 Hz, 1H, 6-O***H***); **<u>^{13}C NMR</u>** (126 MHz, $CDCl_3$): δ 137.64 (Ar***C***CH_2, OBn), 135.13 (Ar***C***CH_2, ONap), 133.84 (Ar***C***S, SPh), 133.27 (Ar***C***C_2, ONap), 133.08 (Ar***C***C_2, ONap), 131.89 (SPh), [129.15, 128.48, 128.44, 127.95, 127.90, 127.74 (SPh; OBn; ONap)], [126.77, 126.28, 126.11, 125.73 (ONap)], 86.00 (***C-1***), 79.67 (***C-3***), 75.58 (***C-2***), 73.32 (***C-5***), 72.19 (Ph***C***H_2, OBn), 71.86 (Ar***C***H_2, ONap), 67.29 (***C-4***), 62.66 (***C-6***); **<u>HR-ESI-TOF/MS (m/z)</u>:** $[M+Na]^+$ calcd for $C_{30}H_{30}O_5SNa$, 525.17116; found, 525.16993.

Phenyl 2-*O*-benzyl-3-*O*-(naphthalene-2-ylmethyl)-1-thio-α-D-mannopyranosiduronic acid (10)

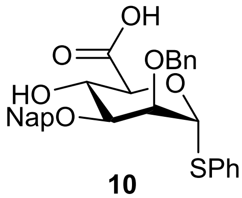

10

Thioglycoside **9** (1.71 g, 3.40 mmol, 1.0 eq) was dissolved in DCM:H_2O (35 mL, 9:1 v/v). BAIB (2.25 g, 6.89 mmol, 2.0 eq) and TEMPO (122 mg, 781 µmol, 0.23 eq) were added, after which the solution was stirred at R.T. for 150 min. The reaction was quenched with 10% aq. $Na_2S_2O_3$ (40 mL) and stirred for an additional 10 min. The mixture was acidified to pH <3 with citric acid and extracted with EtOAc (3x 40 mL). The combined organic layers were dried with $MgSO_4$, filtered and evaporated *in vacuo*. The residue was purified using silica-flash column chromatography (0 – 60% EtOAc in Hept + 2% AcOH), yielding mannuronic acid **10** as a pale yellow foam (1.16 g, 2.25 mmol, 66%).

<u>TLC</u>: (EtOAc:Hept:AcOH, 68:30:2 v/v): R_f = 0.27; **<u>^{1}H NMR</u>** (500 MHz, $CDCl_3$): δ 7.84 – 7.77 (m, 4H, 4x Ar***H***, ONap), 7.50 – 7.43 (m, 3H, 3x Ar***H***, ONap), 7.40 – 7.36 (m, 2H, 2x Ar***H***, SPh), 7.32 – 7.24 (m, 8H, 3x Ar***H***, SPh; 5x Ar***H***, OBn), 5.58 (d, *J* = 2.1 Hz, 1H, ***H-1***), 4.84 (d, *J* = 12.0 Hz, 1H, ArC$\boldsymbol{H_a}$$H_b$, ONap), 4.76 (d, *J* = 12.1 Hz, 1H, ArC$H_a$$\boldsymbol{H_b}$, ONap), 4.66 (d, *J* = 12.1 Hz, 1H, PhC$\boldsymbol{H_a}$$H_b$, OBn), 4.62 (d, *J* = 9.4 Hz, 1H, ***H-5***), 4.59 (d, *J* = 12.1 Hz, 1H, PhC$H_a$$\boldsymbol{H_b}$, OBn), 4.37 (t, *J* = 9.2 Hz, 1H, ***H-4***), 3.97 (dd, *J* = 3.0, 2.2 Hz, 1H, ***H-2***), 3.79 (dd, *J* = 9.1, 3.0 Hz, 1H, ***H-3***); **<u>^{13}C NMR</u>** (126 MHz, $CDCl_3$): δ 172.10 (***C-6***), 137.44 (Ar***C***CH_2, OBn), 135.17 (Ar***C***CH_2-, ONap), 133.26 (Ar***C***C_2, ONap), 133.07 (Ar***C***C_2, ONap), 132.98 (Ar***C***S, SPh), 131.85 (SPh), [129.30, 128.48, 128.35, 128.04, 128.02, 127.97, 127.95, 127.73 (SPh; OBn; ONap)], [126.77, 126.22, 126.06, 125.80 (ONap)], 86.20 (***C-1***), 78.21 (***C-3***), 75.79 (***C-2***), 72.78 (Ar***C***H_2, ONap), 72.53 (Ph***C***H_2, OBn), 71.33 (***C-5***), 68.48 (***C-4***); **<u>HR-ESI-TOF/MS (m/z)</u>:** $[M+Na]^+$ calcd for $C_{30}H_{28}O_6SNa$, 539.15043; found, 539.14912.

Phenyl 2-*O*-benzyl-1-thio-α-D-mannopyranosiduronic acid (11)

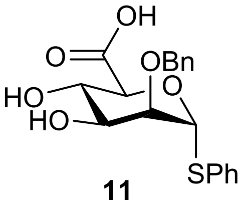

11

Thioglycoside **10** (1.10 g, 2.13 mmol, 1.0 eq) was dissolved in DCM:H_2O (20 mL, 9:1 v/v). DDQ (758 mg, 3.34 mmol, 1.6 eq) was added. The solution was vigorously stirred under exclusion of light for 120 min. The reaction was quenched with 10% aq. $Na_2S_2O_3$ (40 mL) and stirred for an additional 10 min. The mixture was diluted with DCM (20 mL) and acidified to pH <3 with citric acid. The mixture was extracted with DCM (3x 40 mL). The combined organic layers were dried with $MgSO_4$, filtered and evaporated *in vacuo*. The residue was purified using silica-flash column chromatography (0 – 10% MeOH in DCM + 2% AcOH) and subsequently recrystallized from toluene, yielding uronic acid **11** as an off-white solid (502 mg, 1.33 mmol, 62.6%).

<u>TLC</u>: (MeOH:DCM:AcOH, 10:88:2 v/v): R_f = 0.30; **<u>^{1}H NMR</u>** (500 MHz, MeOD-d_4): δ 7.47 – 7.43 (m, 2H, 2x Ar***H***, SPh), 7.42 – 7.38 (m, 2H, 2x Ar***H***, OBn), 7.34 – 7.24 (m, 6H, 3x Ar***H***, SPh; 3x Ar***H***, OBn), 5.53 (d, *J* = 2.8 Hz, 1H, ***H-1***), 4.70 (d, *J* = 11.9 Hz, 1H, PhC$\boldsymbol{H_a}$$H_b$, OBn), 4.66 (d, *J* = 11.9 Hz, 1H, PhC$H_a$$\boldsymbol{H_b}$, OBn), 4.44 (d, *J* = 8.5 Hz, 1H, ***H-5***), 4.06 (t, *J* = 8.6 Hz, 1H, ***H-4***), 3.93 (t, *J* = 3.1 Hz, 1H, ***H-2***), 3.81 (dd, *J* = 8.7, 3.3 Hz, 1H, ***H-3***); **<u>^{13}C NMR</u>** (126 MHz, MeOD-d_4; solvent peak ref'd to 49.00): δ 172.78 (***C-6***), 139.37 (Ar***C***CH_2, OBn), 135.19 (Ar***C***S, SPh), 132.82 (SPh), 130.12 (SPh), 129.38 (OBn), 129.30 (OBn), 128.84 (OBn), 128.64 (SPh), 86.97 (***C-1***), 79.85 (***C-2***), 74.81 (***C-5***), 73.72 (Ph***C***H_2, OBn), 72.19 (***C-3***), 70.53 (***C-4***); **<u>HR-ESI-TOF/MS (m/z)</u>:** $[M+Na]^+$ calcd for $C_{19}H_{20}O_6SNa$, 399.08783; found, 399.08757.

4-*O*-Acetyl-2-*O*-benzyl-1-thio-α-D-mannopyranosidurono-6,3-lactone (1)

Thioglycoside **11** (150 mg, 0.399 mmol, 1.0 eq) was dissolved in Ac_2O (5.0 mL). The solution was heated to 70°C and stirred for 18 hrs. The solution was concentrated *in vacuo* and co-evaporated *in vacuo* with toluene thrice. The residue was dissolved in DCM (20 mL) and washed with aq. $NaHCO_3$ (sat.). The organic layer was dried with $MgSO_4$ and filtered. Activated charcoal was added, after which the mixture was stirred for 15 min. The mixture was filtered over celite and evaporated in vacuo, yielding monosaccharide **1** as a colorless oil (155 mg, 0.387 mmol, 97%).

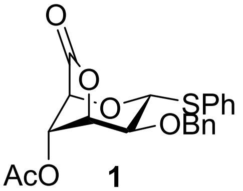

1

TLC: (80:20, EtOAc:Hept v/v): R_f = 0.68; **^{1}H NMR** (500 MHz, $CDCl_3$): δ 7.54 – 7.50 (m, 2H, 2x Ar***H***, SPh), 7.38 – 7.29 (m, 8H, 3x Ar***H***, SPh; 5x Ar***H***, OBn), 4.95 (dd, *J* = 5.9, 3.1 Hz, 1H, ***H-4***), 4.90 (d, *J* = 8.9 Hz, 1H, ***H-1***), 4.86 (dt, *J* = 6.0, 1.3 Hz, 1H, ***H-3***), 4.79 (d, *J* = 11.6 Hz, 1H, PhCH_aH_b, OBn), 4.67 (d, *J* = 11.6 Hz, 1H, PhCH_aH_b, OBn), 4.43 (dd, *J* = 3.1, 1.1 Hz, 1H, ***H-5***), 3.69 (dd, *J* = 9.0, 1.5 Hz, 1H, ***H-2***), 1.94 (s, 3H, C***H*₃**, OAc).**^{13}C NMR** (126 MHz, $CDCl_3$; solvent peak ref'd to 77.16): δ 169.41 (***C***=O, OAc), 168.64 (***C-6***), 137.00 (Ar***C***CH₂, OBn), 133.71 (SPh), 131.46 (Ar***C***S, SPh), [129.11, 128.68, 128.59, 128.38, 128.26 (SPh; OBn)], 84.06 (***C-1***), 76.67 (***C-3***), 73.14 (Ph***C***H₂, OBn), 73.04 (***C-2***), 72.11 (***C-5***), 69.91 (***C-4***), 20.48 (***C***H₃, OAc); **HR-ESI-TOF/MS (m/z):** $[M+Na]^+$ calcd for $C_{21}H_{20}O_6SNa$, 423.0878; found, 423.0878.

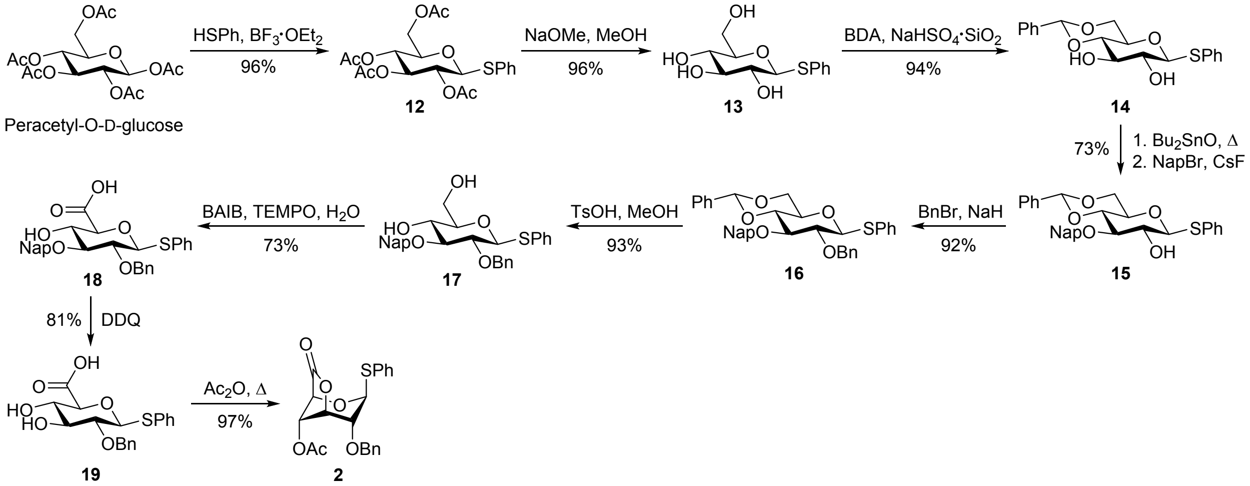

Scheme S3: Synthesis of 6,3-glucuronic acid lactone **2**.

Phenyl 2,3,4,6-tetra-*O*-acetate-1-thio-β-D-glucopyranoside (12)

β-D-Glucose pentaacetate (50.0 g, 128 mmol, 1.0 eq) was dissolved in anhydrous DCM (250 mL). The solution was cooled down to 0°C. Thiophenol (33.0 mL, 324 mmol, 2.5 eq) and $BF_3 \cdot OEt_2$ (48.0 mL, 384 mmol, 3.0 eq) were added. The solution was stirred at R.T. for 41 hrs, turning pink over time. The reaction mixture was poured on ice (250 mL). The organic layer was washed with 1.0 M aq. NaOH (100 mL), aq. $NaHCO_3$ (sat.) (100 mL). The organic layer was dried with $MgSO_4$, filtered and evaporated *in vacuo*. The residue was recrystallized from EtOH, yielding thioglycoside **12** as white crystals (54.0 g, 128 mmol, 96%).

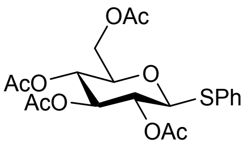

12

TLC: (EtOAc:Hept, 50:50 v/v): R_f = 0.40; **^{1}H NMR** (500 MHz, $CDCl_3$): δ 7.52 – 7.47 (m, 2H, 2x Ar***H***, SPh), 7.35 – 7.29 (m, 3H, 3x Ar***H***, SPh), 5.23 (t, *J* = 9.4 Hz, 1H, ***H-3***), 5.04 (t, *J* = 9.8 Hz, 1H, ***H-4***), 4.98 (t, *J* = 9.7 Hz, 1H, ***H-2***), 4.71 (d, *J* = 10.1 Hz, 1H, ***H-1***), 4.24 – 4.17 (m, 2H, $H\text{-}6_a$, $H\text{-}6_b$), 3.73 (ddd, *J* = 10.1, 5.1, 2.6 Hz, 1H, ***H-5***), 2.09 (s, 3H, C***H*₃**, OAc), 2.08 (s, 3H, C***H*₃**, OAc), 2.02 (s, 3H, C***H*₃**, OAc), 1.99 (s, 3H, C***H*₃**, OAc); **^{13}C NMR** (126 MHz, $CDCl_3$): δ [170.59, 170.20, 169.41, 169.27 (***C***=O)], 133.14 (SPh), 131.65 (Ar***C***S), 128.95 (SPh), 128.44 (SPh), 85.76 (***C-1***), 75.81 (***C-5***), 73.97 (***C-4***), 69.95 (***C-2***), 68.22 (***C-3***), 62.16 (***C-6***), [20.76, 20.74, 20.60, 20.59 (***C***H₃, OAc)]; **HR-ESI-TOF/Ms (m/z):** $[M+Na]^+$ calcd for $C_{20}H_{24}O_9SNa$, 463.1033; found 463.10387.

Phenyl 1-thio-β-D-glucopyranoside (13)

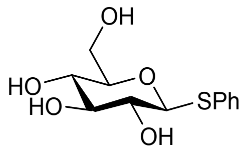

13

Thioglycoside **12** (51.0 g, 116 mmol, 1.0 eq) was dissolved in MeOH (1.10 L). K_2CO_3 (3.30 g, 23.9 mmol, 0.20 eq) was added. The mixture was stirred at R.T. for 66 hrs. DOWEX® 50WX8(H^+) resin was added, after which the mixture was stirred for an additional 30 min. The mixture was filtered and evaporated *in vacuo*, yielding monosaccharide **13** as a white solid (30.0 g, 116 mmol, 96.4%).

TLC: (MeOH:DCM, 20:80 v/v): R_f = 0.60; **^{1}H NMR** (500 MHz, MeOD-d_4; solvent peak ref'd to 3.31)**;** δ 7.59 – 7.52 (m, 2H, 2x Ar***H***, SPh), 7.34 – 7.21 (m, 3H, 3x Ar***H***, SPh), 4.60 (dd, *J* = 9.7, 2.4 Hz, 1H, ***H-1***), 3.87 (dt, *J* = 12.2, 2.2 Hz, 1H, ***H-6$_a$***), 3.67 (ddd, *J* = 12.1, 5.4, 2.2 Hz 1H, ***H-6$_b$***), 3.39 (td, *J* = 8.7, 2.2 Hz, 1H, ***H-3***), 3.34 – 3.28 (m, 2H, ***H-4***, ***H-5***), 3.22 (ddd, *J* = 9.5, 8.5, 2.3 Hz, 1H, ***H-2***); **^{13}C NMR** (126 MHz, MeOD-d_4; solvent peak ref'd to 49.00): δ 135.28 (ArCS), [132.70, 129.87, 128.31 (SPh)], 89.56 (***C-1***), 82.19(***C-5***), 79.84 (***C-4***), 73.92 (***C-2***), 71.50 (***C-3***), 63.02 (***C-6***); **HR-ESI-TOF/Ms(m/z):** $[M+Na]^+$ calcd for $C_{12}H_{16}O_5SNa$, 295.06065; found, 295.06161.

Phenyl 4,6-*O*-benzylidene-1-thio-β-D-glucopyranoside (14)

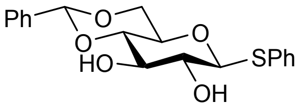

14

Thioglycoside **13** (20 g, 73 mmol, 1.0 eq) added to anh. ACN (0.75 L). Benzaldehyde dimethyl acetal (13 mL, 90 mmol, 1.2 eq) and $NaHSO_4{\cdot}SiO_2$ (42% wt, 4.0 g, 9.3 mmol 0.08 eq) were added, The mixture was stirred at R.T. for 90 min, after which it was quenched with TEA (35 mL). The catalyst was removed by filtration over celite. The filtrate was evaporated *in vacuo*. The residue was dissolved in EtOAc (300 mL). The solution washed with aq. $NaHCO_3$ (sat.) (100 mL) and brine (100 mL), respectively. The organic layer was dried with $MgSO_4$, filtered and evaporated *in vacuo*, yielding monosaccharide **14** as a white solid (25g, 69 mmol, 94%).

TLC: (EtOAc:Tol, 40:60 v/v): R_f = 0.50; **^{1}H NMR** (500 MHz, $CDCl_3$): δ 7.58 – 7.51 (m, 2H, 2x Ar***H***, SPh), 7.50 – 7.44 (m, 2H, 2x Ar***H***, $PhCHO_2$), 7.37 – 7.33 (m, 6H, 3x Ar***H***, SPh; 3x Ar***H***, $PhCHO_2$), 5.52 (s, 1H, PhC***H***O_2), 4.62 (d, J = 9.8 Hz, 1H, ***H-1***), 4.41 – 4.34 (m, 1H, 1H, ***H-6$_a$***), 3.86 – 3.73 (m, 2H, ***H-3***, ***H-6$_b$***), 3.54 – 3.48 (m, 2H, ***H-4***, ***H-5***), 3.45 (t, *J* = 9.1 Hz, 1H, ***H-2***), 2.93 (bs, 1H, O***H***), 2.76 (bs, 1H, O***H***); **^{13}C NMR** (126 MHz, $CDCl_3$): δ 136.86 (ArCCHO_2), 133.07 (SPh), 131.31 (Ar***C***S, SPh), 129.36 ($PhCHO_2$), [129.15, 128.49, 128.39 (SPh; $PhCHO_2$)], 126.30 ($PhCHO_2$), 101.95 (Ph***C***HO_2), 88.60 (***C-1***), 80.20 (***C-4***), 74.58 (***C-3***), 72.60 (***C-2***), 70.54 (***C-5***), 68.58 (***C-6***); **HR-ESI-TOF/Ms (m/z):** $[M+Na]^+$ calcd for $C_{19}H_{20}O_5Sna$, 383.09291; found 383.09425.

Phenyl 3-*O*-(naphthalene-2-ylmethyl)-4,6-*O*-benzylidene-1-thio-β-D-glucopyranoside (15)

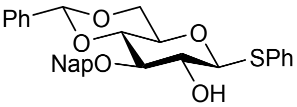

15

Thioglycoside **14** (19.7 g, 54.6 mmol, 1.0 eq) and Bu_2SnO (14.9 g, 60.1 mmol, 1.1 eq) were suspended in anh. toluene (150 mL). The suspension was refluxed for 41 hrs, after which the resulting clear solution was concentrated *via* distillation. The solution was cooled down to R.T. and diluted with anh. DMF (200 mL). CsF (14.4 g, 95.3 mmol, 1.7eq) and NapBr (18.1g, 81.9mmo, 1.5 eq) were added. The mixture was stirred for 3 hrs. The mixture was concentrated *in vacuo* and then diluted with DCM (1.00 L). Next, it was washed with water (400 mL) and $NaHCO_3$ (sat.) (400 mL). The organic layer was dried with Na_2SO_4, filtered and evaporated *in vacuo*. The residue was recrystallized from EtOH, yielding monosaccharide **15** as white crystals (20.0 g, 40.0 mmol, 73%).

TLC: (EtOAc:Heptane, 50:50 v/v): R_f = 0.53; **^{1}H NMR** (500 MHz, $CDCl_3$)**:** δ 7.83 – 7.77 (m, 3H, 3x Ar***H***, ONap), 7.75 – 7.71 (m, 1H, Ar***H***, ONap), 7.56 – 7.51 (m, 2H, 2x Ar***H***, SPh), 7.51 – 7.43 (5H, 5x Ar***H***), 7.40 – 7.36 (m, 3H, 3x Ar***H***), 7.32 – 7.31 (m, 3H, Ar***H***), 5.59 (s, 1H, PhC***H***O_2) 5.10 (d, *J* = 11.8 Hz, 1H, PhC***H$_a$***H_b, ONap), 4.96 (d, *J* = 11.8 Hz, 1H, PhCH_a***H$_b$***, ONap), 4.63 (d, *J* = 9.8 Hz, 1H, ***H-1***), 4.39 (dd, *J* = 10.5, 5.0 Hz, 1H, ***H-6$_a$***), 3.80 (t, *J* = 10.3 Hz, 1H, ***H-6$_b$***), 3.77 – 3.66 (m, 2H, ***H-3***, ***H-4***), 3.59 – 3.49 (m, 2H, ***H-2***, ***H-5***), (d, *J* = 2.1 Hz, 1H, 2-O***H***); **^{13}C NMR** (126 MHz, $CDCl_3$): δ 137.20 (Ar***C***CHO_2), 135.59 (Ar***C***CH_2, ONap), 133.27 (Ar***C***C_2, ONap), 133.20 (SPh), 133.07 (Ar***C***C_2, ONap), 131.28 (Ar***C***S, SPh), [129.06, 128.40, 128.31, 128.28, 127.95, 127.68, 126.96, 126.07 (SPh, $PhCHO_2$, ONap)], [126.01, 125.94 (ONap)], 101.38 (Ph***C***HO_2), 88.56 (***C-1***), 81.46 (***C-3***), 81.10 (***C-4***), 74.82 (Ar***C***H_2, ONap), 72.38 (***C-2***), 70.79 (***C-5***), 68.66 (***C-6***); **HR-ESI-TOF/Ms (m/z):** $[M+Na]^+$ calcd for $C_{30}H_{28}O_5SNa$, 523.1551; found, 523.15373.

Phenyl 2-*O*-benzyl-3-*O*-(naphthalene-2-ylmethyl)-4,6-*O*-benzylidene-1-thio-β-D-glucopyranoside (16)

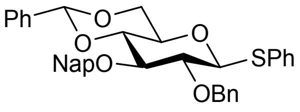

16

Thioglycoside **15** (5.0 g, 9.9 mmol, 1,0 eq) was dissolved in anh. DMF (150 mL). NaH (1.0 g, 60% wt, 25 mmol, 2.5 eq) added at 0°C. BnBr (1.5 mL, 12 mmol, 1.2 eq) was added. The mixture was stirred at 0°C R.T. for 63 hrs. The reaction was quenched with aq. NH_4Cl (90 mL) and concentrated *in vacuo*. The residue was resuspended in DCM (200 mL). The solution washed with water (100 mL) and brine (100 mL), respectively. The organic layer dried with Na_2SO_4, filtered and evaporated *in vacuo*. The residue was recrystallized from EtOH, yielding monosaccharide **16** as white crystals (5.4 g, 9.1 mmol, 92%).
TLC: (EtOAc: Hep, 25:75, v/v): R_f = 0.55; **^{1}H NMR** (500 MHz, $CDCl_3$): δ 7.82 – 7.78 (m, 1H, Ar***H***, ONap), 7.77 – 7.72 (m, 2H, 2x Ar***H***, ONap), 7.70 – 7.65 (m, 1H, Ar***H***, ONap), 7.56 – 7.51 (m, 2H, 2x Ar***H***, SPh), 7.51 – 7.41 (m, 5H, 5x Ar***H***, 3x Ar***H***, ONap; 2x Ar***H***, $PhCHO_2$), 7.41 – 7.28 (m, 11H, 11x Ar***H***, 3x Ar***H***, SPh; 3x Ar***H***, $PhCHO_2$, 5x Ar***H***, OBn), 5.61 (s, 1H, PhC***H***O_2), 5.08 (d, *J* = 11.4 Hz, 1H, ArC***H****a*H_b, ONap), 4.94 (d, *J* = 11.4 Hz, 1H, ArCH_a***H****b*, ONap), 4.89 (d, *J* = 10.3 Hz, 1H, PhC***H****a*H_b, OBn), 4.89 (d, *J* = 10.3 Hz, 1H, PhCH_a***H****b*, OBn), 4.77 (d, *J* = 9.7 Hz, 1H, ***H-1***), 4.39 (dd, *J* = 10.5, 5.0 Hz, 1H, ***H-6****a*), 3.89 (dd, *J* = 9.4, 8.3 Hz, 1H, ***H-3***), 3.82 (t, *J* = 10.3 Hz, 1H, ***H-6****b*), 3.74 (t, *J* = 9.4 Hz, 1H, ***H-4***), 3.54 (dd, *J* = 9.8, 8.3 Hz, 1H, ***H-2***), 3.48 (td, *J* = 9.7, 5.0 Hz, 1H, ***H-5***); **^{13}C NMR** (126 MHz, $CDCl_3$): δ 138.03 (Ar***C***CH_2, OBn), 137.27 (Ar***C***CHO_2), 135.75 (Ar***C***CH_2, ONap), 133.29 (Ar***C***S, SPh), [133.14, 133.04 (Ar***C***C_2, ONap)], 132.34 (SPh), [129.04, 128.42, 128.30, 128.20, 128.14, 127.96, 127.88, 127.67 (SPh; ONap; OBn; $PhCHO_2$)], [126.87, 126.22 (ONap)], 126.06 ($PhCHO_2$), [126.01, 125.88 (ONap)], 101.25 (Ph***C***HO_2), 88.35 (***C-1***), 82.94 (***C-3***), 81.47 (***C-4***), 80.56 (***C-2***), 75.92 (Ph***C***H_2, OBn), 75.34 (Ar***C***H_2, ONap), 70.28 (***C-5***), 68.73 (***C-6***); **HR-ESI-TOF/Ms (m/z):** $[M+Na]^+$ calcd for $C_{37}H_{34}O_5SNa$, 613.18871; found, 613.18863.

Phenyl 2-*O*-benzyl-3-*O*-(naphthalene-2-ylmethyl)-1-thio-β-D-glucopyranoside (17)

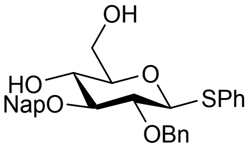

17

Thioglycoside **16** (2.8 g, 4.8 mmol, 1.0 eq) dissolved in DCM/MeOH (1:3 v/v, 70 mL). *p*-TsOH·H_2O (0.10 g, 0.53 mmol, 0.11 eq). The solution was stirred at R.T. for 42 hrs. The reaction was quenched with TEA (1.0 mL) and evaporated *in vacuo*. The residue was dissolved in DCM (50 mL) and washed with aq. $NaHCO_3$ (sat.) (50 mL) and brine (50 mL), respectively. The organic layer was dried with $MgSO_4$, filtered and evaporated *in vacuo*. The residue was recrystallized from EtOAc/Hep, yielding monosaccharide **17** as a white solid (2.3 g, 4.5 mmol, 93%).
TLC: (EtOAc: Hep, 60:40, v/v): R_f = 0.50; **^{1}H NMR** (500 MHz, $CDCl_3$) δ 7.85 – 7.76 (m, 3H, 3x Ar***H***, ONap), 7.75 – 7.72 (m, 1H, Ar***H***, ONap), 7.54 – 7.27 (m, 13H, 3x Ar***H***, ONap; 5x Ar***H***, SPh; 5x Ar***H***, OBn), 5.11 (d, *J* = 11.7 Hz, 1H, ArC***H****a*H_b, ONap), 4.98 (d, *J* = 10.3 Hz, 1H, PhC***H****a*H_b, OBn), 4.88 (d, *J* = 11.7 Hz, 1H, ArCH_a***H****b*, ONap), 4.77 (d, *J* = 10.3 Hz, 1H, PhCH_a***H****b*, OBn), 4.74 (d, *J* = 9.6 Hz, 1H, ***H-1***), 3.87 (ddd, *J* = 11.9, 6.5, 3.5 Hz, 1H, ***H-6****a*), 3.75 (ddd, *J* = 12.0, 6.8, 5.3 Hz, 1H, ***H-6****b*), 3.62 (td, *J* = 8.9, 2.5 Hz, 1H, ***H-4***), 3.58 (t, *J* = 8.6 Hz, 1H, ***H-3***), 3.52 (dd, *J* = 9.6, 8.4 Hz, 1H, ***H-2***), 3.35 (ddd, *J* = 9.0, 5.3, 3.4 Hz, 1H, ***H-5***), 2.33 (d, *J* = 2.7 Hz, 1H, 4-O***H***), 2.03 (t, *J* = 6.6 Hz, 1H, 6-O***H***); **^{13}C NMR** (126 MHz, $CDCl_3$; solvent peak ref'd to 77.16): δ 137.94 (Ar***C***CH_2, OBn), 135.77 (Ar***C***CH_2, ONap), 133.66 (Ar***C***S, SPh), [133.44, 133.20 (Ar***C***C_2, ONap)], 131.90 (SPh), [129.22, 128.76, 128.64, 128.42, 128.15, 128.08, 127.90, 127.85 (SPh; ONap; OBn)], [126.92, 126.46, 126.27, 125.75 (ONap)], 87.92 (***C-1***), 86.09 (***C-3***), 81.09 (***C-2***), 79.27 (***C-5***), [75.63, 75.59 (Ar***C***H_2, ONap; Ph***C***H_2, OBn)], 70.64 (***C-4***), 62.92 (***C-6***); **HR-ESI-TOF/Ms (m/z):** $[M+Na]^+$ calcd for $C_{30}H_{30}O_5SNa$, 525.17116; found, 525.16820.

Phenyl 2-*O*-benzyl-3-*O*-(naphthalene-2-ylmethyl)-1-thio-β-D-glucopyranosiduronic acid (18)

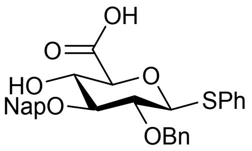

18

Thioglycoside **17** (2.0 g, 3.9 mmol, 1.0 eq) was dissolved in (30 mL, 9:1 v/v, DCM:H_2O). BAIB (2.5g, 7.9 mmol, 2.0 eq) and TEMPO (0.15 g, 0.99 mmol, 0.25 eq) were added. The solution was stirred at R.T. for 3 hrs. The reaction was quenched with 10% aq. $Na_2S_2O_3$ (20 mL) and stirred for an additional 10 min. The mixture was acidified to pH <3 with citric acid and extracted with EtOAc. The combined organic layers were dried with $MgSO_4$, filtered and evaporated *in vacuo*. The residue was purified using silica-flash column chromatography (20 – 60% EtOAc in Hept + 2% AcOH), yielding glucuronic acid **18** as a white solid (1.5 g, 2.9 mmol, 73%).
TLC: (EtOAc:Hep:AcOH, 60:38:2 v/v) R_f = 0.50; **^{1}H NMR** (500 MHz, $CDCl_3$) δ 7.83 – 7.74 (m, 4H, 4x Ar***H***, ONap), 7.56 – 7.53 (m, 2H, 2x Ar***H***, SPh), 7.48 – 7.44 (m, 3H, 3x Ar***H***, ONap), 7.39 – 7.28 (m, 8H, 3x Ar***H***, SPh; 5x Ar***H***, OBn), 5.07 (d,

J = 11.3 Hz, 1H, ArC***H**ₐ*H_b, ONap), 4.99 (d, *J* = 11.4 Hz, 1H, ArCH$_a$***H**$_b$*, ONap), 4.88 (d, *J* = 10.4 Hz, 1H, PhC***H**$_a$*H_b, OBn), 4.79 (d, *J* = 10.4 Hz, 1H, PhCH$_a$***H**$_b$*, OBn), 4.74 (d, *J* = 9.7 Hz, 1H, ***H-1***), 3.93 – 3.83 (m, 2H, ***H-4***, ***H-5***), 3.67 (t, *J* = 8.6 Hz, 1H, ***H-3***), 3.50 (dd, *J* = 9.7, 8.6 Hz, 1H, ***H-2***); **^{13}C NMR** (126 MHz, $CDCl_3$; solvent peak ref'd to 77.16): δ 171.56 (***C-6***), 137.88 (Ar***C***CH_2, OBn), 135.72 (Ar***C***CH_2, ONap), [133.43, 133.20 (Ar***C***C_2, ONap)], 132.81 (SPh), 132.71 (Ar***C***S, SPh), [129.36, 128.60, 128.46, 128.43, 128.34, 128.12, 128.10, 127.84 (SPh; ONap; OBn)], [127.03, 126.27, 126.17, 126.13 (ONap)], 88.32 (***C-1***), 85.07 (***C-3***), 79.66 (***C-2***), 76.45 (***C-5***), [75.85, 75.80 (Ar***C***H_2, ONap; Ph***C***H_2, OBn)], 72.04 (***C-4***); **HR-ESI-TOF/MS (m/z):** [M+Na]$^+$ calcd for $C_{30}H_{28}O_6SNa$ 539.15043; found, 539.15143.

Phenyl 2-*O*-benzyl-1-thio-β-D-glucopyranosiduronic acid (19)

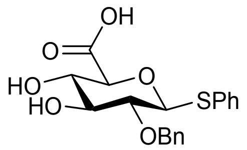

19

Thioglycoside **18** (1.5 g, 2.9 mmol, 1.0 eq) was dissolved in DCM:H_2O (20 mL, 9:1 v/v). DDQ (0.90 g, 4.0 mmol, 1.4 eq) was added. The mixture was vigorously stirred under exclusion of light for 50 min. The reaction was quenched with 10% aq. $Na_2S_2O_3$ and stirred for an additional 10 min. The mixture was diluted with DCM (20 mL) and acidified to pH <3 with citric acid. The mixture was extracted with DCM. The combined organic layers were dried with $MgSO_4$, filtered and evaporated *in vacuo*. The residue was purified using silica-flash column chromatography (0 – 20% MeOH in DCM + 2% AcOH), yielding uronic acid **19** as an off-white solid (0.83 g, 2.2 mmol, 81%).

TLC: (MeOH:DCM:AcOH, 20:78:2 v/v): R_f = 0.80; **^{1}H NMR** (500 MHz, MeOD-d_4; solvent peak ref'd to 3.31) δ 7.58 – 7.51 (m, 2H, 2x Ar***H***, SPh), 7.47 – 7.41 (m, 2H, 2x Ar***H***, OBn), 7.36 – 7.23 (m, 6H, 3x Ar***H***, SPh; 3x ArH, OBn), 4.84 (d, *J* = 10.5 Hz, 1H, PhC***H**$_a$*H_b, OBn), 4.77 (d, *J* = 10.7 Hz, 1H, PhCH$_a$***H**$_b$*, OBn), 4.73 (d, *J* = 9.7 Hz, 1H, ***H-1***), 3.78 (d, *J* = 8.6 Hz, 1H, ***H-5***), 3.63 – 3.53 (m, 2H, ***H-3***, ***H-4***), 3.33 – 3.27 (m, 1H, ***H-2***); **^{13}C NMR** (126 MHz, MeOD-d_4;solvent peak ref'd to 77.16): δ 173.5 (***C-6***), 139.80 (Ar***C***CH_2, OBn), 135.07 (Ar***C***S, SPh), 132.96 (SPh), [129.99, 129.19, 129.18, 128.67, 128.61 (SPh; OBn)], 88.84, 81.64, 80.22, 79.30, 76.13, 73.24; **HR-ESI-TOF/MS (m/z):** [M+Na]$^+$ calcd for $C_{19}H_{20}O_6SNa$, 399.08783; found, 399.08814

4-*O*-Acetyl-2-*O*-benzyl-1-thio-α-D-glucopyranosidurono-6,3-lactone (2)

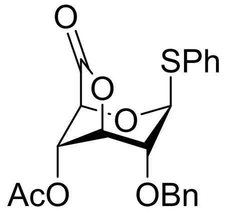

2

Thioglycoside **19** (0.10 g, 0.27 mmol, 1.0 eq) was dissolved in Ac_2O (6.0 mL). The solution was heated to 70°C and stirred for 3 hrs. The solution was concentrated *in vacuo* and co-evaporated *in vacuo* with toluene thrice. The residue was dissolved in DCM (10 mL) and washed with aq. $NaHCO_3$ (sat.) (10 mL). The organic layer was dried with Na_2SO_4 and filtered. Activated charcoal was added, after which the mixture was stirred for 10 min. The mixture was filtered over celite and evaporated in vacuo, yielding monosaccharide **2** as a colorless oil (157 mg, 0.39 mmol, 98%).

TLC: (80:20, EtOAc:Hept v/v): R_f = 0.68; **^{1}H NMR** (500 MHz, $CDCl_3$): δ 7.52 – 7.46 (m, 2H, 2x Ar***H***, SPh), 7.39 – 7.29 (m, 8H, 8x Ar***H***, SPh; 5x Ar***H***, OBn), 5.59 (d, *J* = 2.3 Hz, 1H, ***H-1***), 5.12 (ddd, *J* = 5.3, 3.1, 1.0 Hz, 1H, ***H-3***), 4.96 (ddd, *J* = 5.3, 3.3, 1.0 Hz, 1H, ***H-4***), 4.60 (d, *J* = 11.2 Hz, 1H, PhC***H**$_a$*H_b, OBn), 4.56 (d, *J* = 11.2 Hz, 1H, PhCH$_a$***H**$_b$*, OBn), 4.25 (d, *J* = 3.4 Hz, 1H, ***H-5***), 4.08 – 4.02 (m, 1H, ***H-2***), 1.91 (s, 3H, C***H**$_3$*, OAc); **^{13}C NMR** (126 MHz, $CDCl_3$): δ 170.58, 169.70, 136.59 (Ar***C***CH_2, OBn), 133.30 (SPh), 132.72 (Ar***C***S, SPh), [129.18, 128.72, 128.54, 128.33 (SPh; OBn)], 85.37 (***C-1***), 76.82 (***C-2***), 73.48 (Ph***C***H_2, OBn), 72.27 (***C-3***), 68.83 (***C-5***), 68.50 (***C-4***), 20.54 (***C***H_3, OAc); **HR-ESI-TOF/MS (m/z):** [M+Na+H_2O]$^+$ calcd for $C_{21}H_{20}O_6SNa$, 441.0984; found, 441.0990.

6) Supporting NMR data

Mannuronic acid lactone – EXSY rates at varying temperature

^{19}F EXSY results at -40 °C

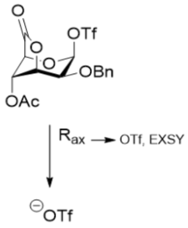

Spectrum number	Mixing Time [s]	β-triflate (-75.74 ppm) resonance intensity	$^{-}$OTf (-78.93 ppm) resonance intensity
1	0.1	20398.9	108.368
2	0.2	17166.3	204.946
3	0.3	14520	270.463
4	0.4	12157.2	326.889
5	0.5	10239.7	344.858
6	0.6	8676.58	346.004
7	0.7	7375.69	328.176

[$^{-}$OTf] = 0.074 M

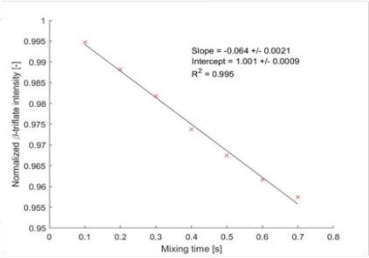

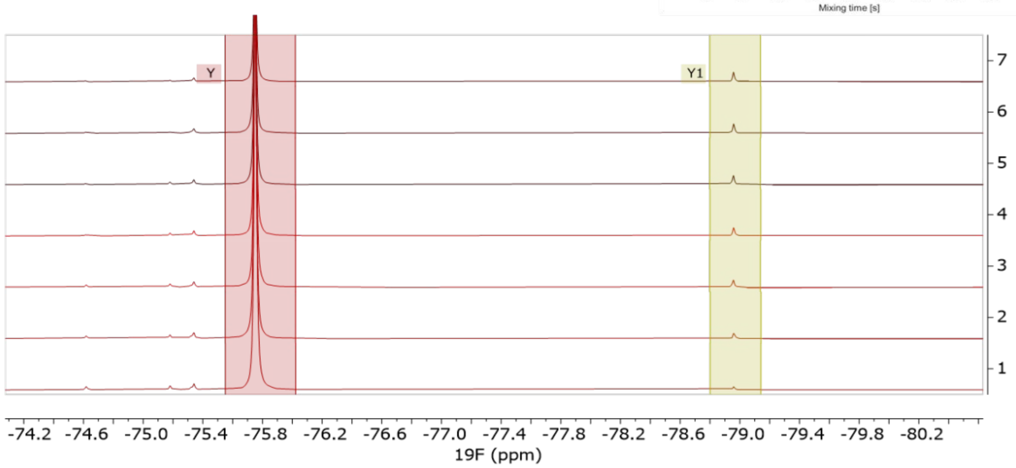

Figure S19: ^{19}F EXSY data at -40°C.

^{19}F EXSY results at -30 °C

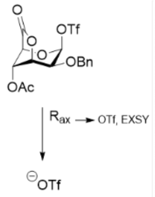

Spectrum number	Mixing Time [s]	β-triflate (-75.74 ppm) resonance intensity	$^{-}$OTf (-78.93 ppm) resonance intensity
1	0.06	21308.9	188.332
2	0.12	19565.8	364.474
3	0.18	17905.8	479.588
4	0.24	16381.7	638.007
5	0.3	15063.1	751.947
6	0.36	13768.6	856.066
7	0.42	12552.6	869.042
8	0.48	11584.5	962.033
9	0.54	10552.1	943.822
10	0.6	9659.65	981.3

[$^{-}$OTf] = 0.074 M

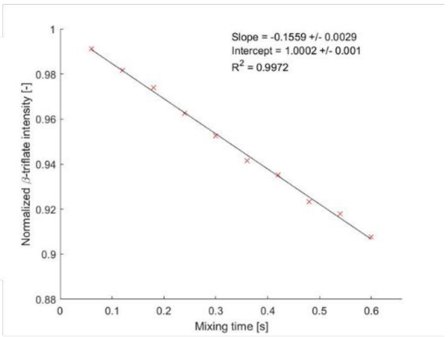

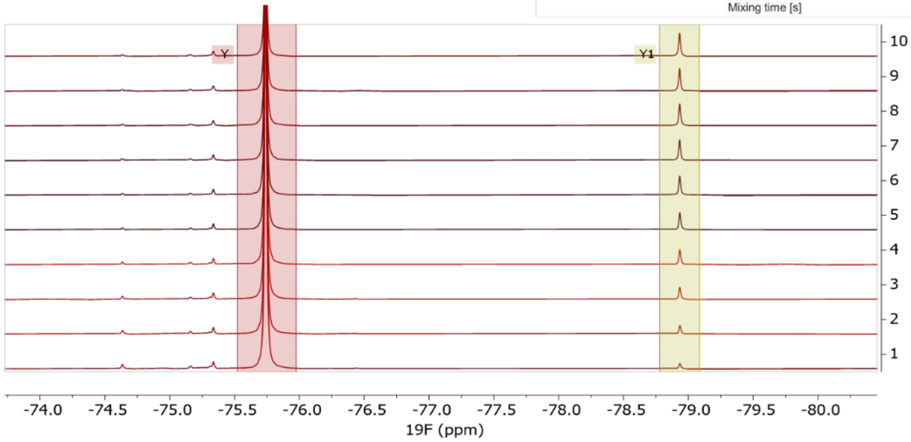

Figure S20: ^{19}F EXSY data at -30°C.

^{19}F EXSY results at -20 °C

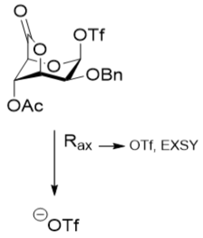

Spectrum number	Mixing Time [s]	β-triflate (-75.74 ppm) resonance intensity	⁻OTf (-78.93 ppm) resonance intensity
1	0.035	36137.5	412.715
2	0.07	34659.3	773.449
3	0.105	32755.1	1084.84
4	0.14	30818.7	1440.1
5	0.175	29033	1702.02
6	0.21	27419.8	1917.09
7	0.245	25901.6	2180.44
8	0.28	24510.5	2394.13
9	0.315	23069.2	2565.16
10	0.35	21786.3	2722.51

[⁻OTf] = 0.074 M

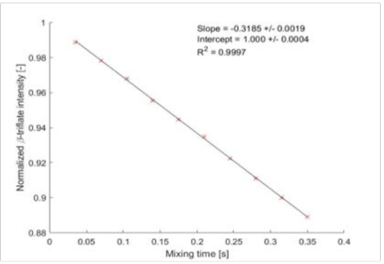

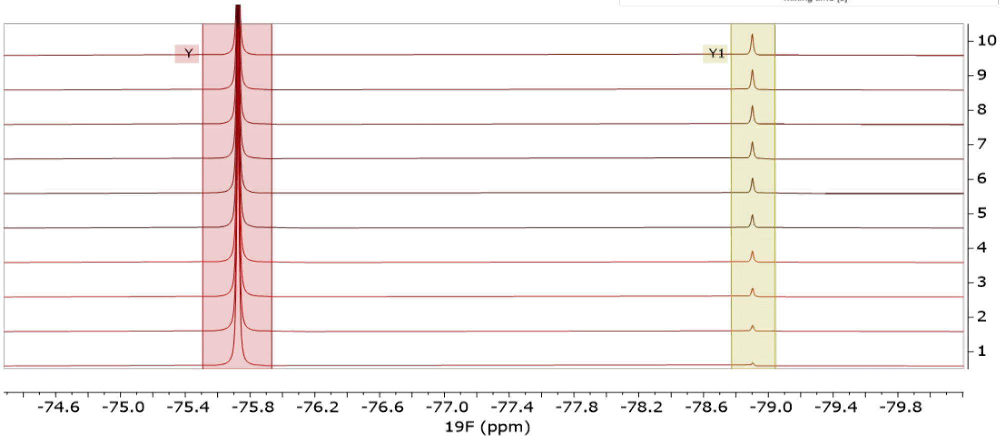

Figure S21: ^{19}F EXSY data of mannuronic acid lactone 1 at -20°C.

^{19}F EXSY at -50 °C

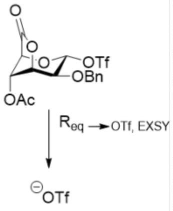

Spectrum number	Mixing Time [s]	α-triflate (-74.64 ppm) resonance intensity	⁻OTf (-78.93 ppm) resonance intensity
1	0.025	7655.25	85.7826
2	0.05	7386.24	113.47
3	0.075	6952.47	146.953
4	0.1	6710.81	186.336
5	0.125	6492.73	212.999
6	0.15	6098.67	268.059
7	0.175	5862.54	293.788
8	0.2	5534.77	302.589

[⁻OTf] = 0.074 M

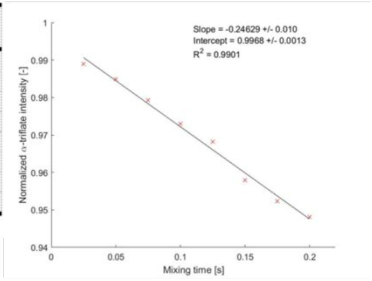

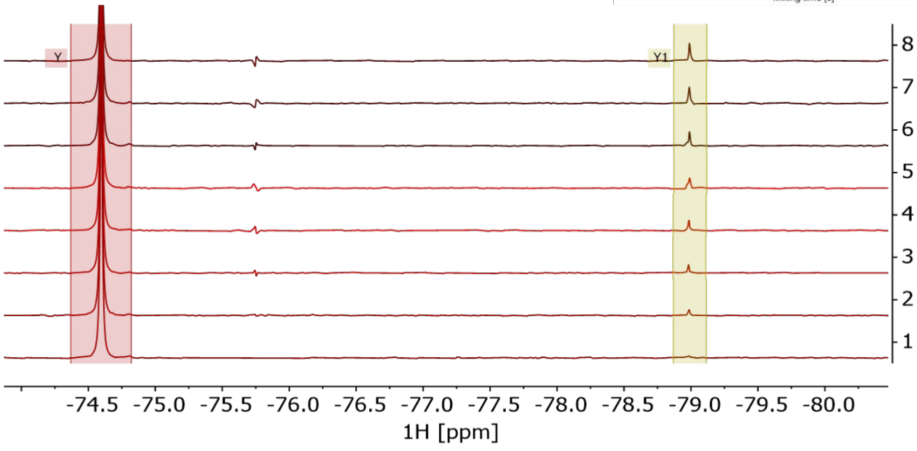

Figure S22: ^{19}F EXSY data of mannuronic acid lactone 1 at -50°C.

^{19}F EXSY at -40 °C

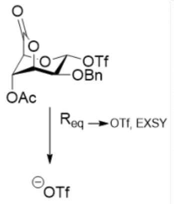

Spectrum number	Mixing Time [s]	α-triflate (-74.64 ppm) resonance intensity	⁻OTf (-78.93 ppm) resonance intensity
1	0.016	9281.71	102.121
2	0.032	9148.36	202.552
3	0.048	8655.7	251.997
4	0.064	8336.9	346.865
5	0.08	8072.39	431.383
6	0.096	7838.21	536.254
7	0.112	7701.97	611.785
8	0.128	7346.94	646.438
9	0.144	7286.38	753.65
10	0.16	7026.14	816.27

[⁻OTf] = 0.074 M

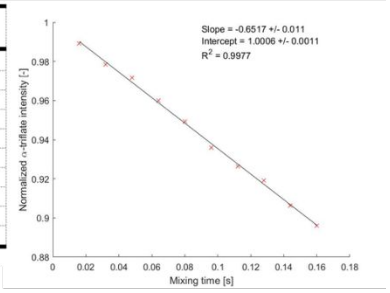

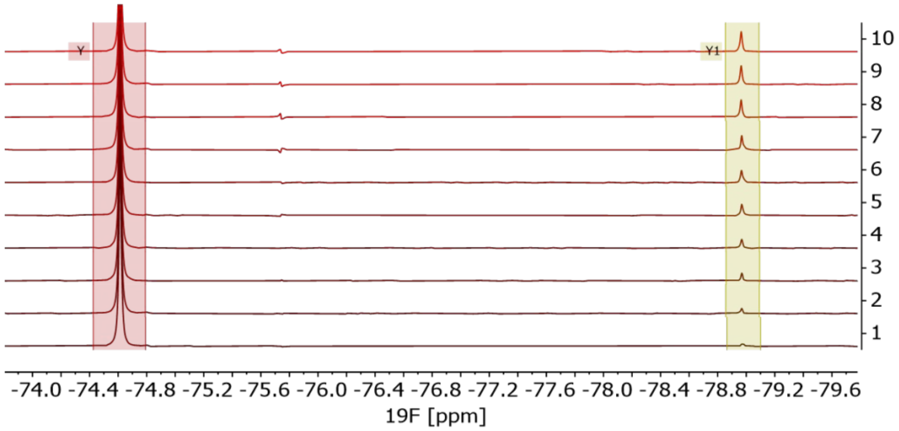

Figure S23: ^{19}F EXSY data of mannuronic acid lactone 1 at -40°C.

^{19}F EXSY at -30 °C

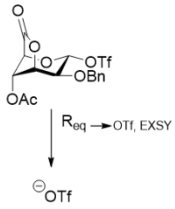

Spectrum number	Mixing Time [s]	α-triflate (-74.64 ppm) resonance intensity	⁻OTf (-78.93 ppm) resonance intensity
1	0.009	10360	162.202
2	0.018	10121.9	291.515
3	0.027	9912.25	428.82
4	0.036	9701.51	459.328
5	0.045	9522.29	632.286
6	0.054	9283.92	737.099
7	0.063	9141.38	869.099
8	0.072	8918.61	957.308
9	0.081	8680.32	1026.4
10	0.09	8468.47	1159.97

[⁻OTf] = 0.074 M

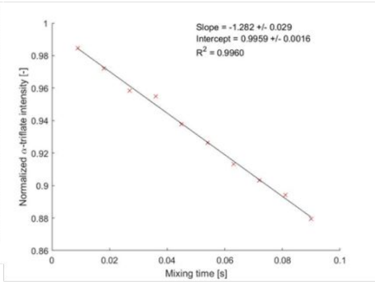

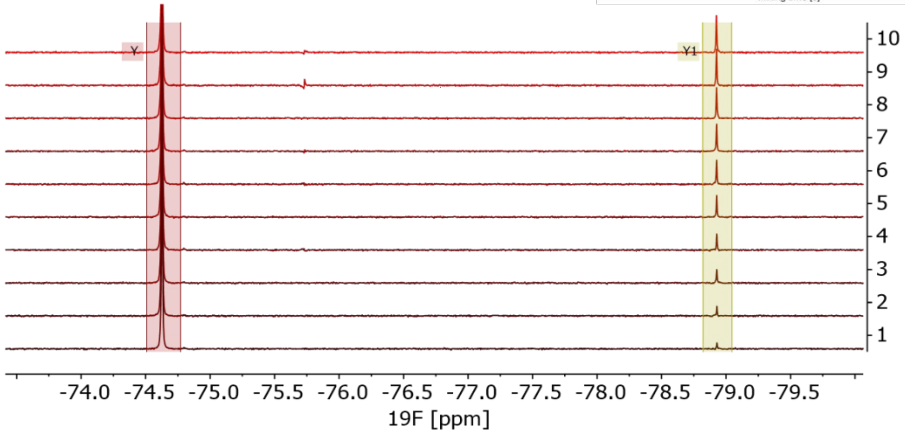

Figure S24: ^{19}F EXSY data of mannuronic acid lactone 1 at -30°C.

^{19}F EXSY at -20 °C

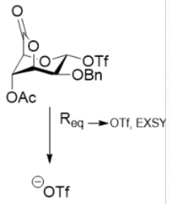

Spectrum number	Mixing Time [s]	α-triflate (-74.64 ppm) resonance intensity	$^{-}$OTf (-78.93 ppm) resonance intensity
1	0.004	10627.9	109.598
2	0.008	9889.78	243.744
3	0.012	9635.67	335.509
4	0.016	9537.37	469.716
5	0.02	8709.52	563.184
6	0.024	8994.33	626.791
7	0.028	8411.03	835.392
8	0.032	8203.81	750.218
9	0.036	7999.43	881.462
10	0.04	7458.75	986.788

[$^{-}$OTf] = 0.074 M

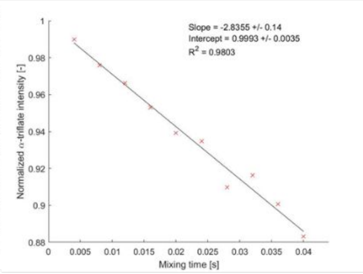

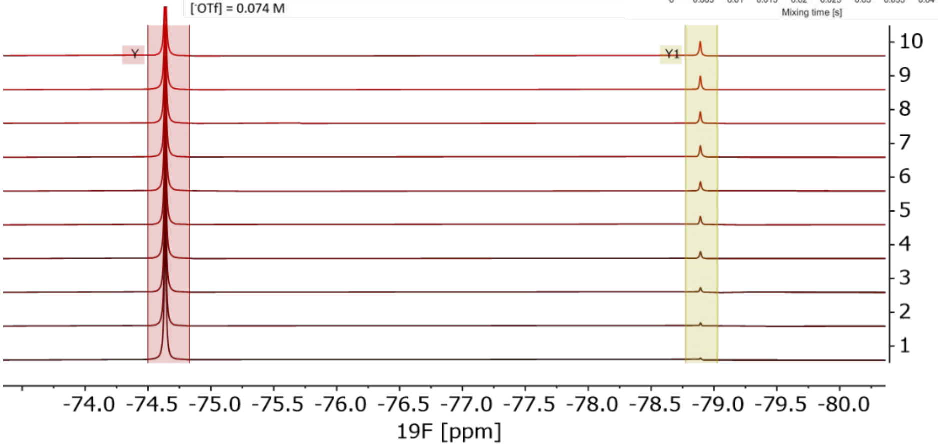

Figure S25: ^{19}F EXSY data of mannuronic acid lactone 1 at -20°C.

Glucuronic acid lactone – EXSY rates at varying temperature

^{19}F EXSY results at -30 °C

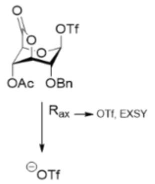

Spectrum number	Mixing Time [s]	β-triflate (-75.64 ppm) resonance intensity	$^{-}$OTf (-78.93 ppm) resonance intensity
1	0.1	33002.1	163.312
2	0.2	29598.7	241.871
3	0.3	26538.6	337.235
4	0.4	23890.5	410.686
5	0.5	21414.2	446.874
6	0.6	19173.8	499.855
7	0.7	17261.2	539.03
8	0.8	15446.1	554.402
9	0.9	13900.6	523.644

[$^{-}$OTf] = 0.081 M

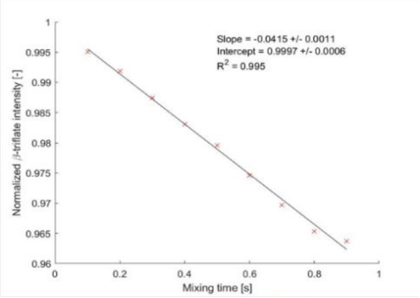

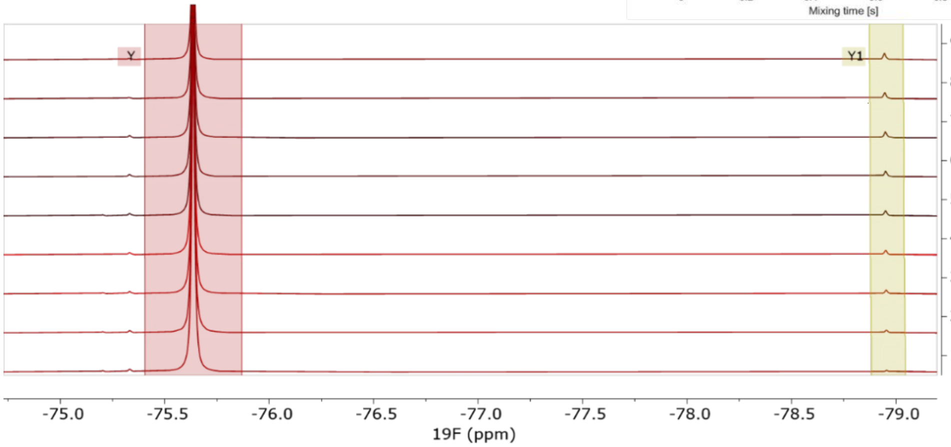

Figure S26: ^{19}F EXSY data of glucuronic acid lactone 2 at -30°C.

^{19}F EXSY results at -20 °C

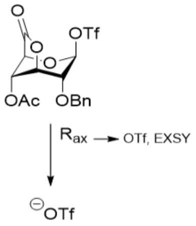

Spectrum number	Mixing Time [s]	β-triflate (-75.64 ppm) resonance intensity	⁻OTf (-78.93 ppm) resonance intensity
1	0.1	30472.5	264.62
2	0.2	27521.1	481.422
3	0.3	24923.8	705.169
4	0.4	22481.7	819.684
5	0.5	20300.8	963.579
6	0.6	18397.4	1060.38
7	0.7	16610.6	1159.9
8	0.8	15009	1197.79
9	0.9	13566.8	1277.88
10	1	12290.2	1348.95

[⁻OTf] = 0.081 M

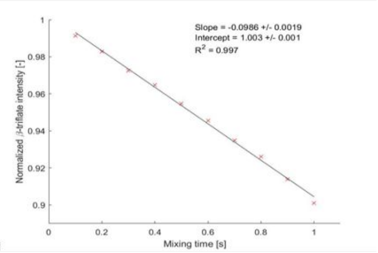

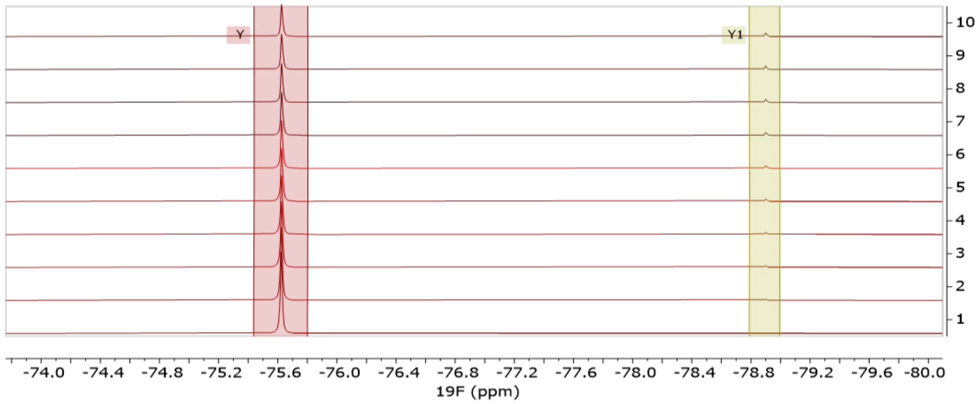

Figure S27: ^{19}F EXSY data of glucuronic acid lactone **2** at -20°C.

Mannuronic acid lactone – EXSY rates at varying concentrations triflate anion

^{19}F EXSY results at [⁻OTf] = 0.074 M

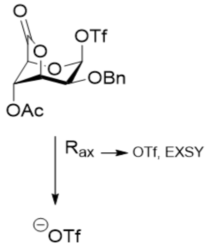

Spectrum number	Mixing Time [s]	β-triflate (-75.74 ppm) resonance intensity	⁻OTf (-78.93 ppm) resonance intensity
1	0.06	21308.9	188.332
2	0.12	19565.8	364.474
3	0.18	17905.8	479.588
4	0.24	16381.7	638.007
5	0.3	15063.1	751.947
6	0.36	13768.6	856.066
7	0.42	12552.6	869.042
8	0.48	11584.5	962.033
9	0.54	10552.1	943.822
10	0.6	9659.65	981.3

Temperature = -30 °C

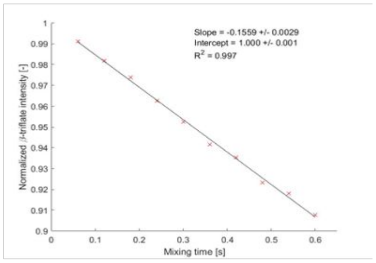

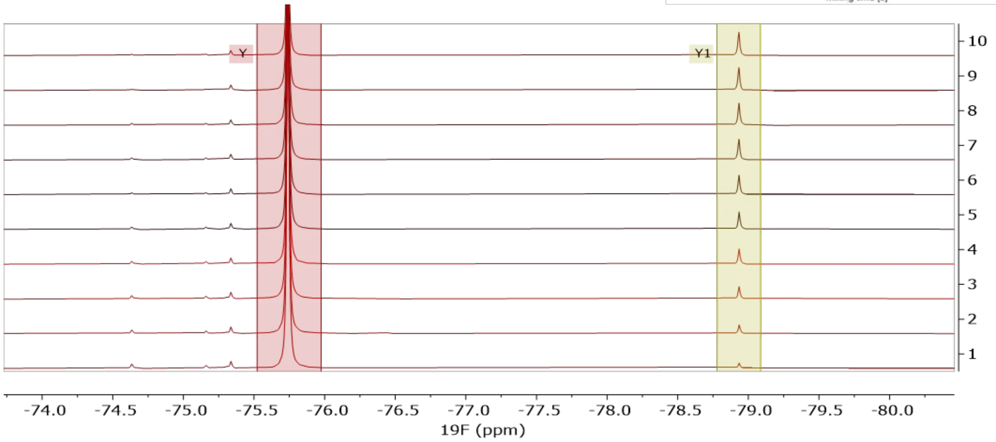

Figure S28: ^{19}F EXSY data of mannuronic acid lactone 1 at triflate concentration = 0.074 M.

^{19}F EXSY results at [$^{-}$OTf] = 0.12 M

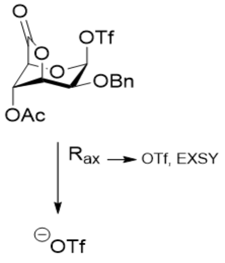

Spectrum number	Mixing Time [s]	β-triflate (-75.74 ppm) resonance intensity	$^{-}$OTf (-78.93 ppm) resonance intensity
1	0.04	15289.5	149.638
2	0.08	14362.4	309.147
3	0.12	13424.1	394.971
4	0.16	12571.6	520.405
5	0.2	11736.8	597.914
6	0.24	10979.6	652.665
7	0.28	10221.3	740.436
8	0.32	9557.53	804.016
9	0.36	8932.45	875.528
10	0.4	8350.72	918.629

Temperature = -30 °C

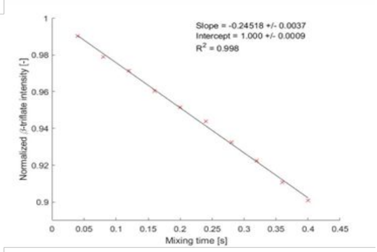

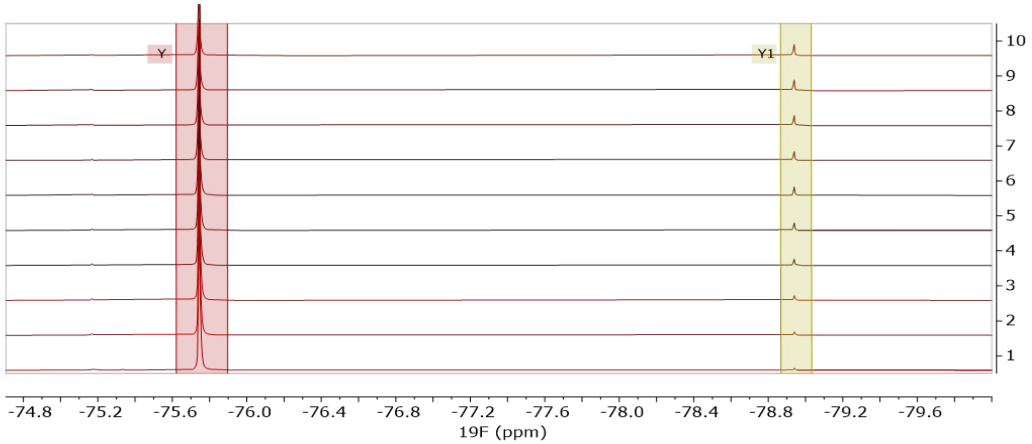

Figure S29: ^{19}F EXSY data of mannuronic acid lactone 1 at triflate concentration = 0.12 M.

^{19}F EXSY results at [$^{-}$OTf] = 0.18 M

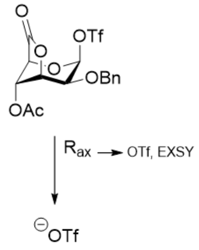

Spectrum number	Mixing Time [s]	β-triflate (-75.74 ppm) resonance intensity	$^{-}$OTf (-78.93 ppm) resonance intensity
1	0.03	24550.1	203.948
2	0.06	22493.3	429.821
3	0.09	21564.1	600.637
4	0.12	20287.4	726.385
5	0.15	19202.1	895.472
6	0.18	18157.2	1021.17
7	0.21	17134.1	1201.02
8	0.24	16328.6	1335.26
9	0.27	15497.9	1401.14
10	0.3	14617.9	1558.24

Temperature = -30 °C

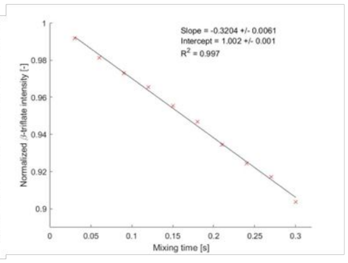

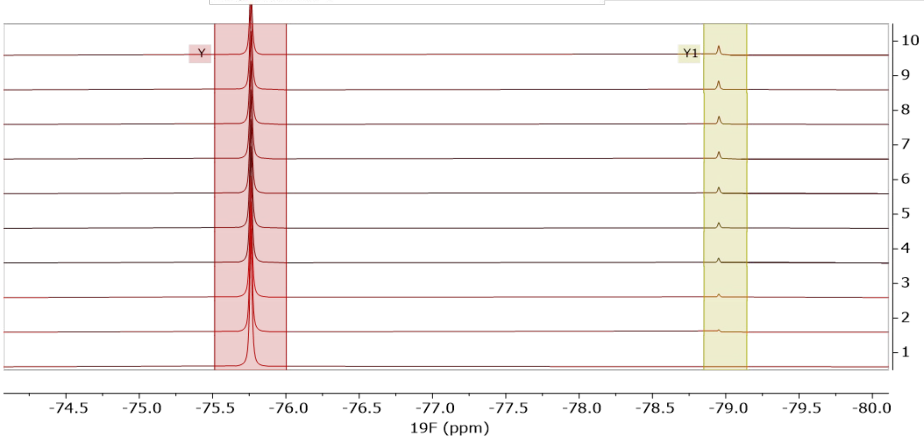

Figure S30: ^{19}F EXSY data of mannuronic acid lactone 1 at triflate concentration = 0.18 M.

[19]F EXSY results at [-OTf] = 0.26 M

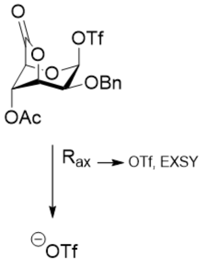

Spectrum number	Mixing Time [s]	β-triflate (-75.74 ppm) resonance intensity	-OTf (-78.93 ppm) resonance intensity
1	0.025	19075.4	251.361
2	0.05	17439.6	513.346
3	0.075	16875.2	520.32
4	0.1	15834.1	671.327
5	0.125	15085.7	853.531
6	0.15	14231.6	946.753
7	0.175	13485.6	1149.6
8	0.2	12771.4	1229.03
9	0.225	12118.3	1341.19
10	0.25	11457.7	1484.8

Temperature = -30 °C

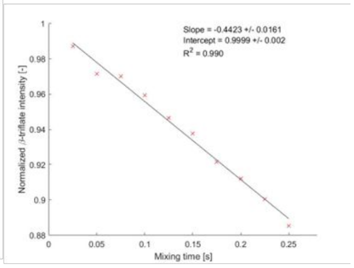

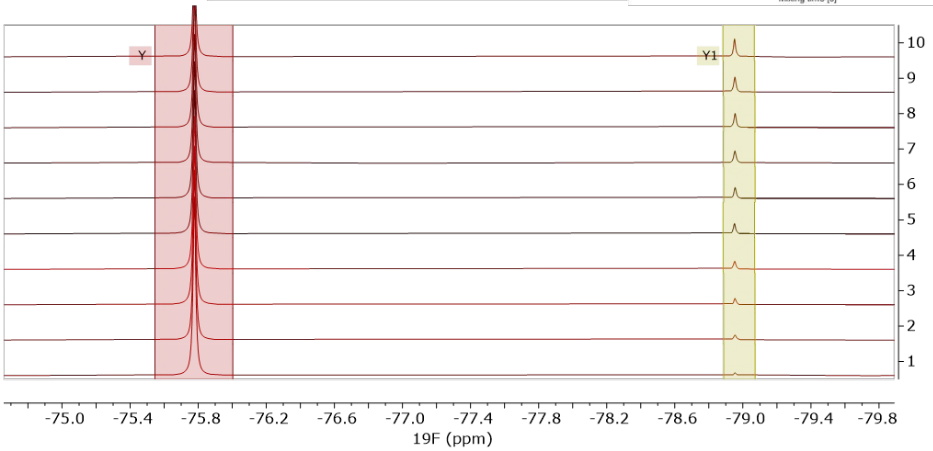

Figure S31: [19]F EXSY data of mannuronic acid lactone 1 at triflate concentration = 0.26 M.

Glucuronic acid lactone – EXSY rates at varying concentrations triflate anion

^{19}F EXSY results at [$^{-}$OTf] = 0.074 M

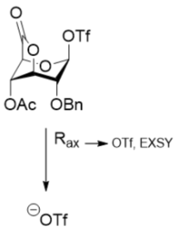

Spectrum number	Mixing Time [s]	β-triflate (-75.64 ppm) resonance intensity	$^{-}$OTf (-78.93 ppm) resonance intensity
1	0.1	70949.8	290.366
2	0.2	63194.6	506.684
3	0.3	55891.8	704.362
4	0.4	49740.4	826.437
5	0.5	44438.2	939.081
6	0.6	39856.9	1068.63
7	0.7	35280.8	1100.55
8	0.8	31491	1113.98
9	0.9	28101.4	1137.57
10	1	25089	1159.9

Temperature = -30 °C

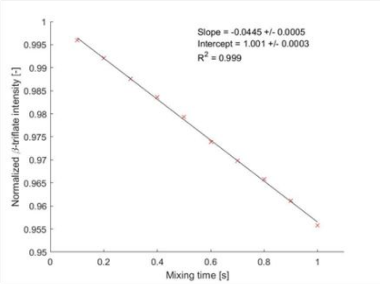

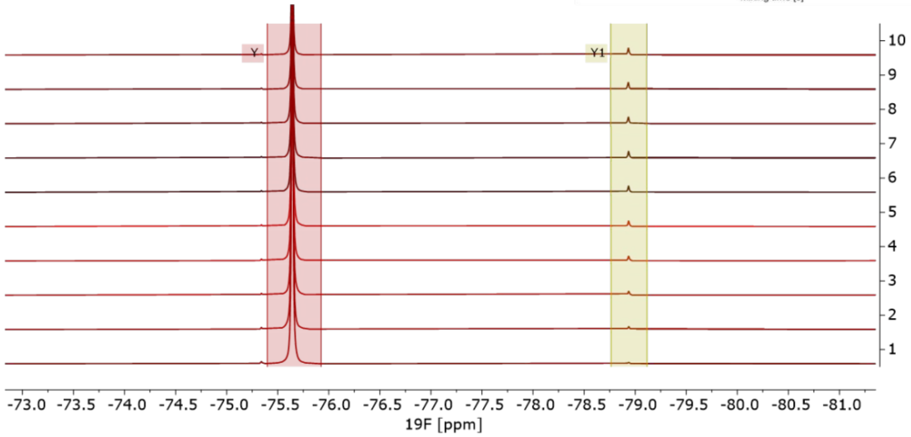

Figure S32: ^{19}F EXSY data of glucuronic acid lactone 2 at triflate concentration = 0.074 M.

^{19}F EXSY results at [$^{-}$OTf] = 0.12 M

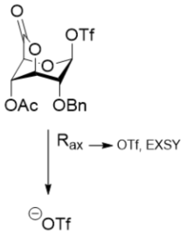

Spectrum number	Mixing Time [s]	β-triflate (-75.64 ppm) resonance intensity	$^{-}$OTf (-78.93 ppm) resonance intensity
1	0.1	60552.8	457.546
2	0.2	53275.8	776.181
3	0.3	46730.3	1110.42
4	0.4	41083.8	1348.57
5	0.5	36239.4	1511.52
6	0.6	31921.2	1686.63
7	0.7	28146.8	1712.94
8	0.8	24861.8	1826.62
9	0.9	21860.5	1889.42
10	1	19538	1878.45

Temperature = -30 °C

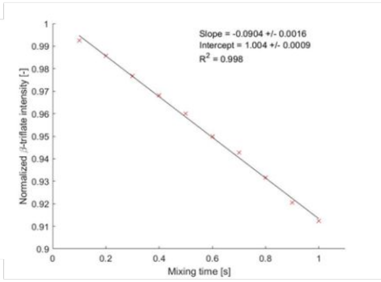

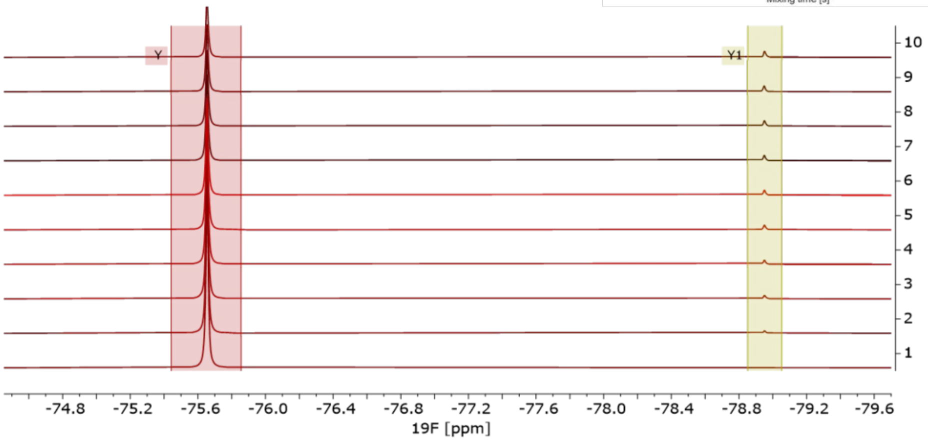

Figure S33: ^{19}F EXSY data of glucuronic acid lactone 2 at triflate concentration = 0.12 M.

^{19}F EXSY results at [$^-$OTf] = 0.18 M

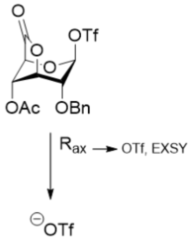

Spectrum number	Mixing Time [s]	β-triflate (-75.64 ppm) resonance intensity	$^-$OTf (-78.93 ppm) resonance intensity
1	0.07	48657.4	481.488
2	0.14	44135.6	840.204
3	0.21	39992.7	1213.5
4	0.28	36216.4	1490.07
5	0.35	32456.7	1711.56
6	0.42	29867.5	1909.86
7	0.49	27009.5	2139.41
8	0.56	24617.8	2290.35
9	0.63	22359.5	2367.59
10	0.7	20170.3	2456.32

Temperature = -30 °C

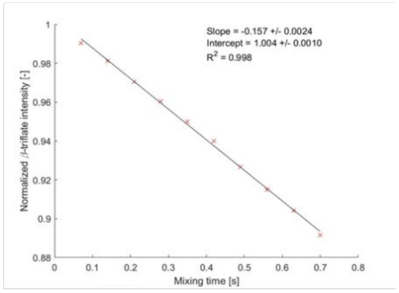

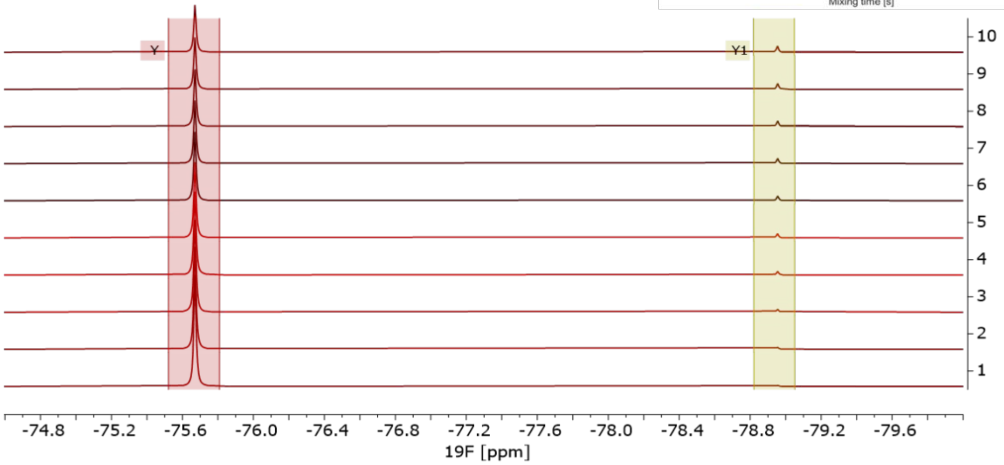

Figure S34: ^{19}F EXSY data of glucuronic acid lactone 2 at triflate concentration = 0.18 M.

^{19}F EXSY results at [$^-$OTf] = 0.26 M

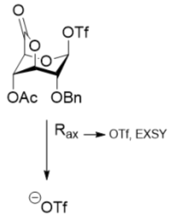

Spectrum number	Mixing Time [s]	β-triflate (-75.64 ppm) resonance intensity	$^-$OTf (-78.93 ppm) resonance intensity
1	0.04	39243.4	322.18
2	0.08	36352.9	687.603
3	0.12	34004.7	971.226
4	0.16	31904.3	1227.6
5	0.2	29743.5	1470.5
6	0.24	27854.5	1654.43
7	0.28	25958.8	1848.06
8	0.32	24358.5	2043.96
9	0.36	22835.3	2213.21
10	0.4	21250.6	2311.33

Temperature = -30 °C

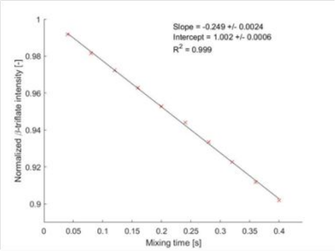

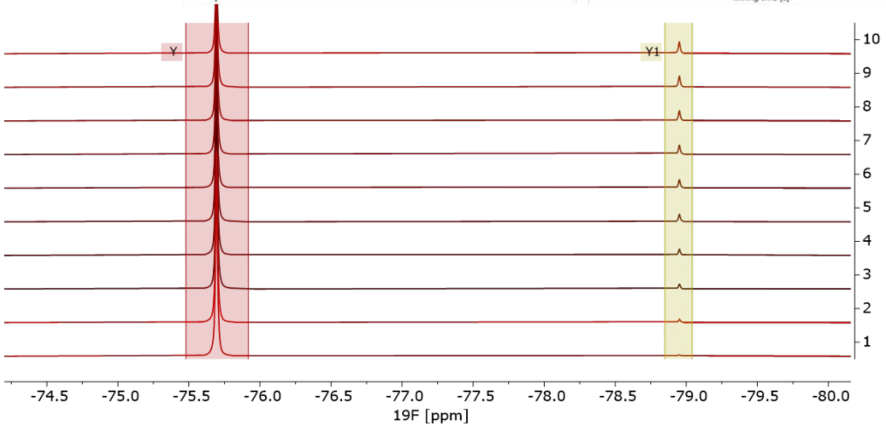

Figure S35: ^{19}F EXSY data of glucuronic acid lactone 2 at triflate concentration = 0.26 M.

7) ^{1}H and ^{13}C NMR spectra

1,2,3,4,6-Penta-*O*-acetyl-D-mannopyranoside (3)

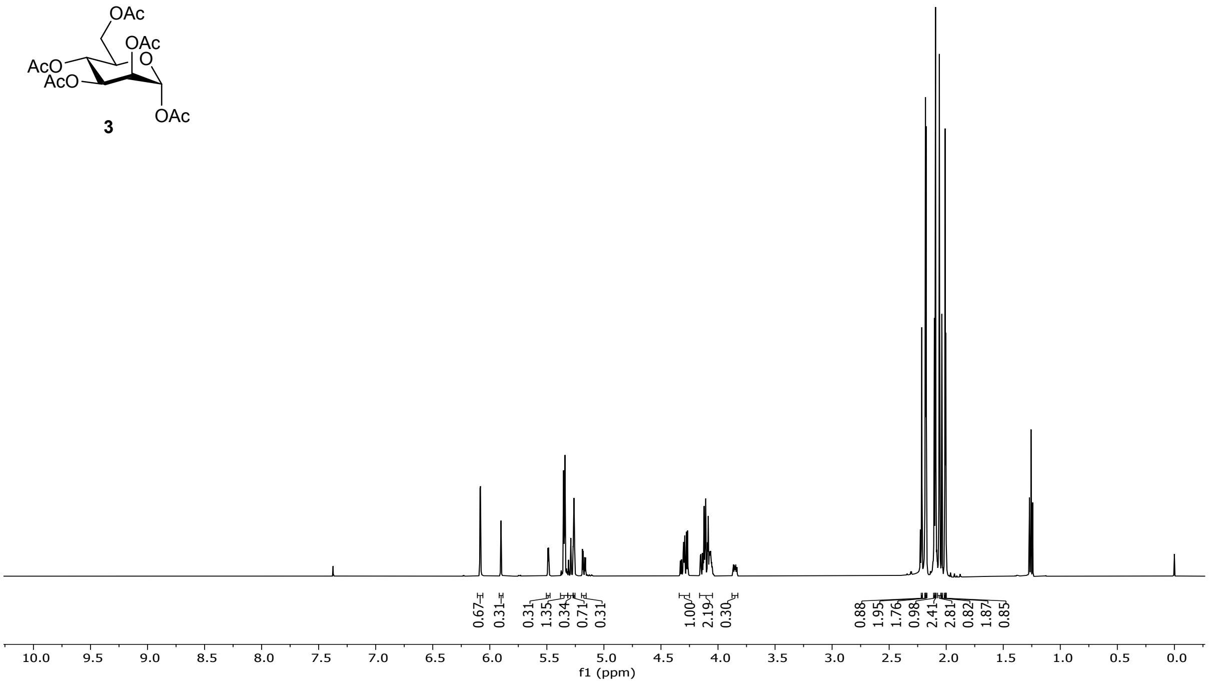

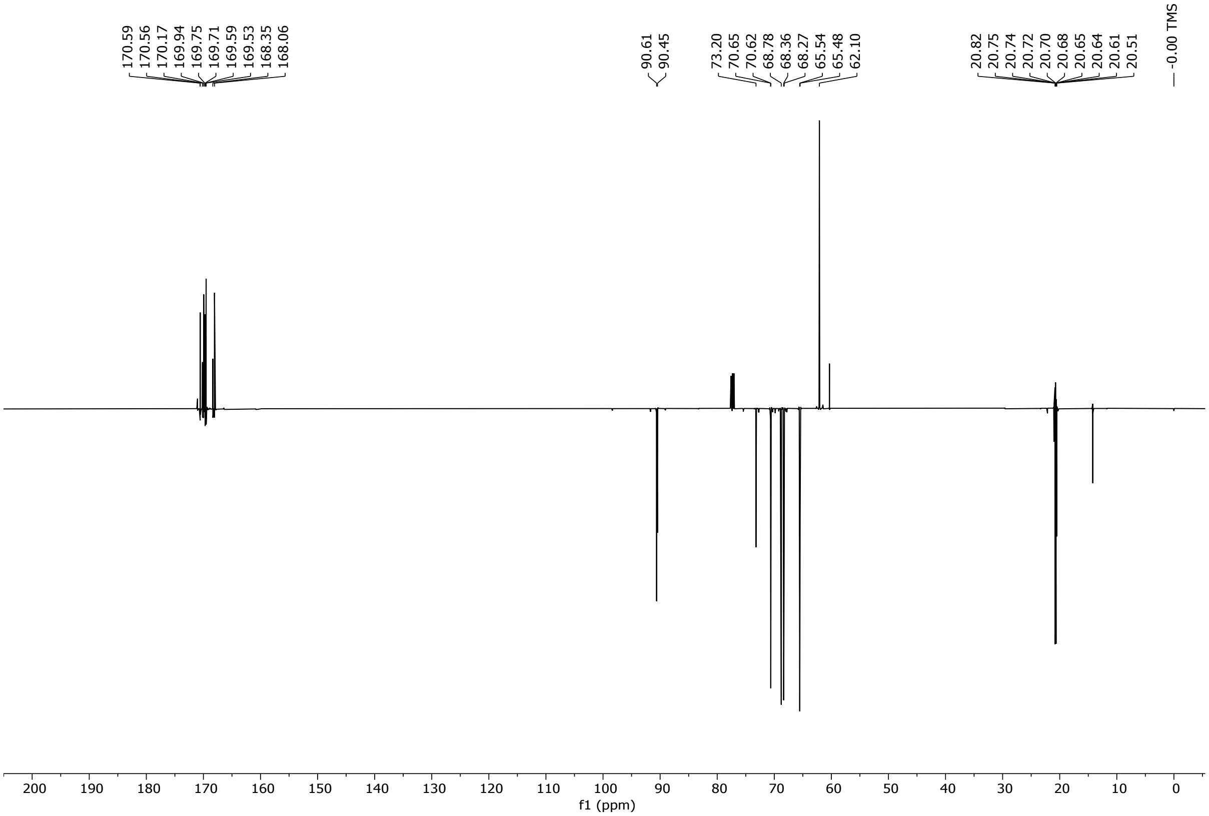

Figure S36: ^{1}H NMR spectrum (top) and ^{13}C NMR spectrum (bottom) of **3**.

Phenyl 2,3,4,6-tetra-*O*-acetyl-1-thio-α-D-mannopyranoside (4)

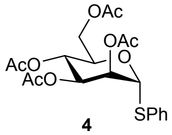

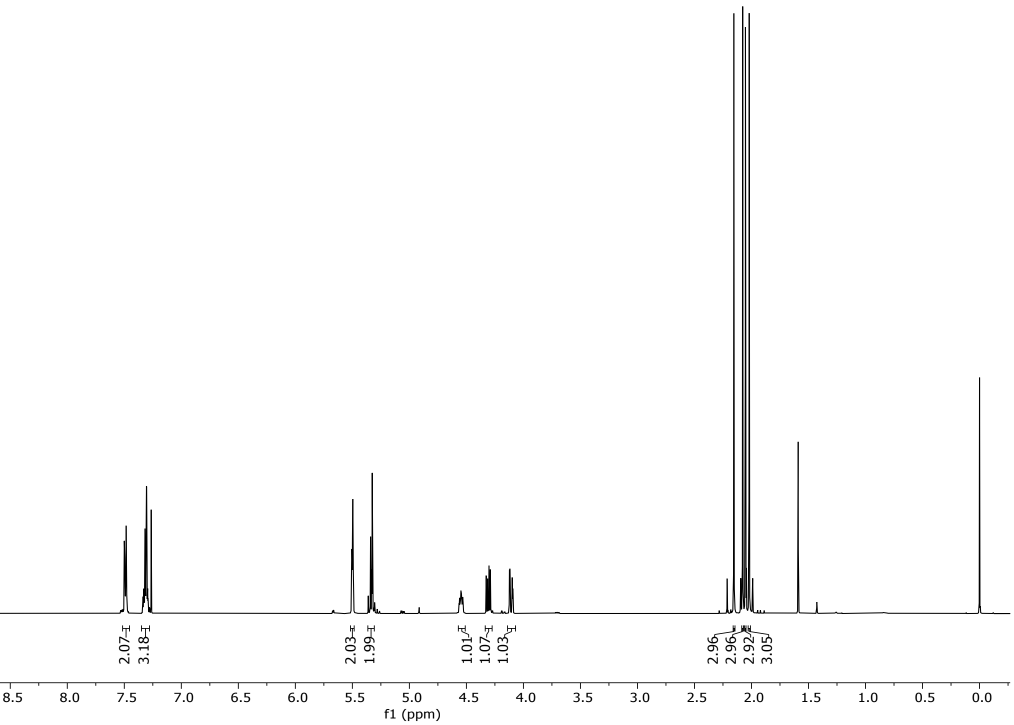

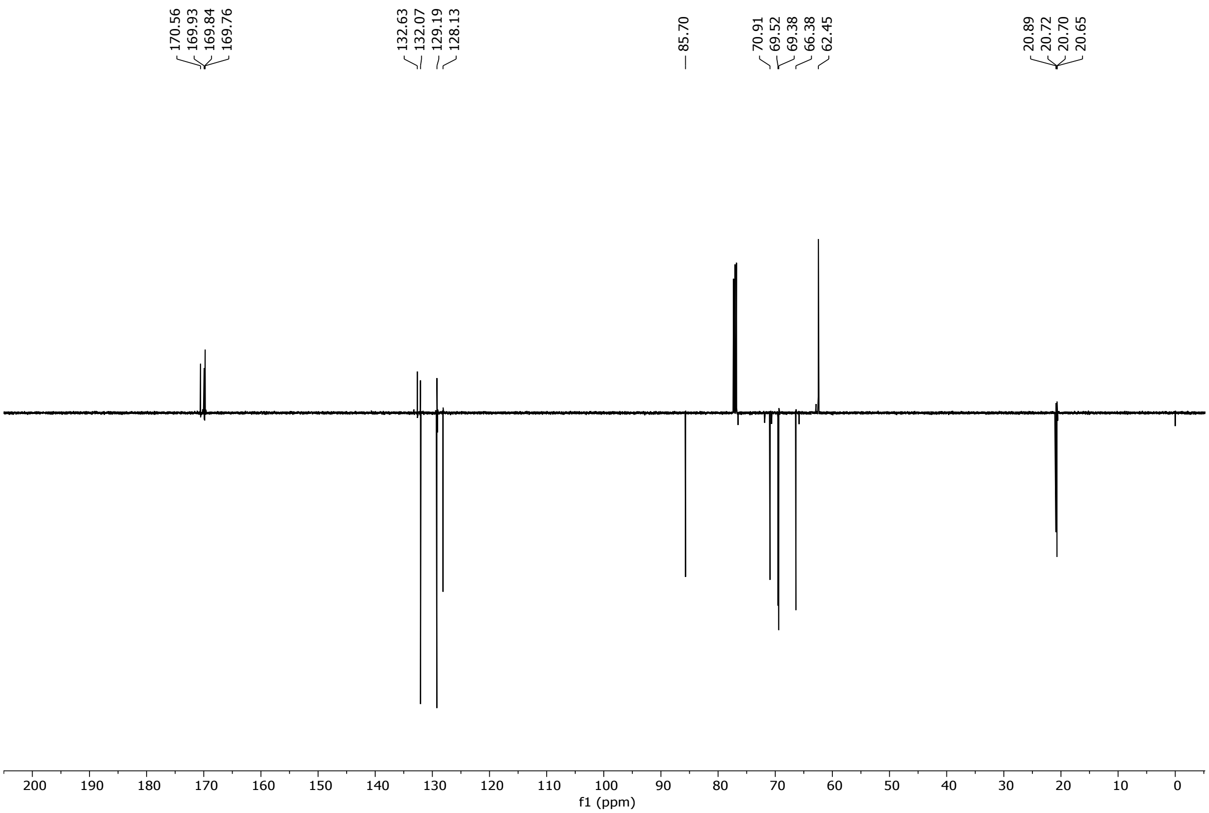

Figure S37: ^{1}H NMR spectrum (top) and ^{13}C NMR spectrum (bottom) of **4**.

Phenyl 1-thio-α-D-mannopyranoside (5)

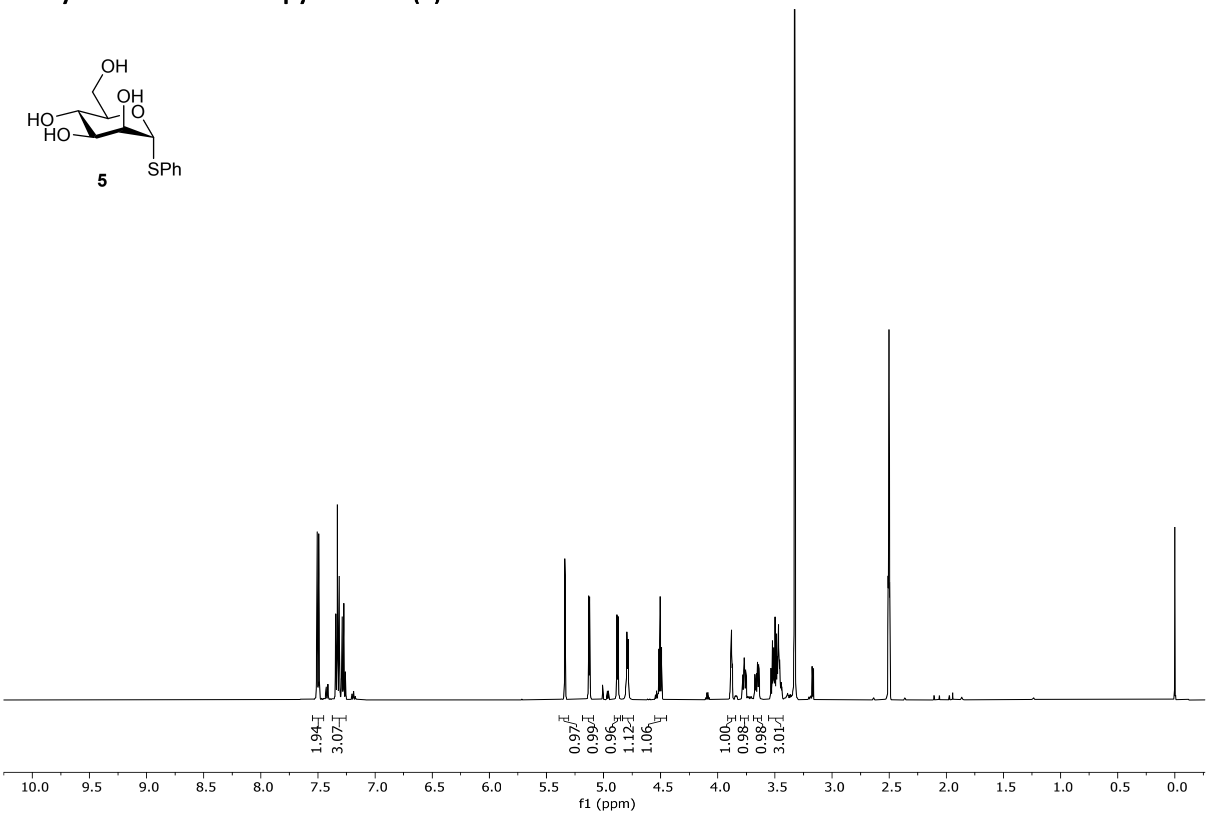

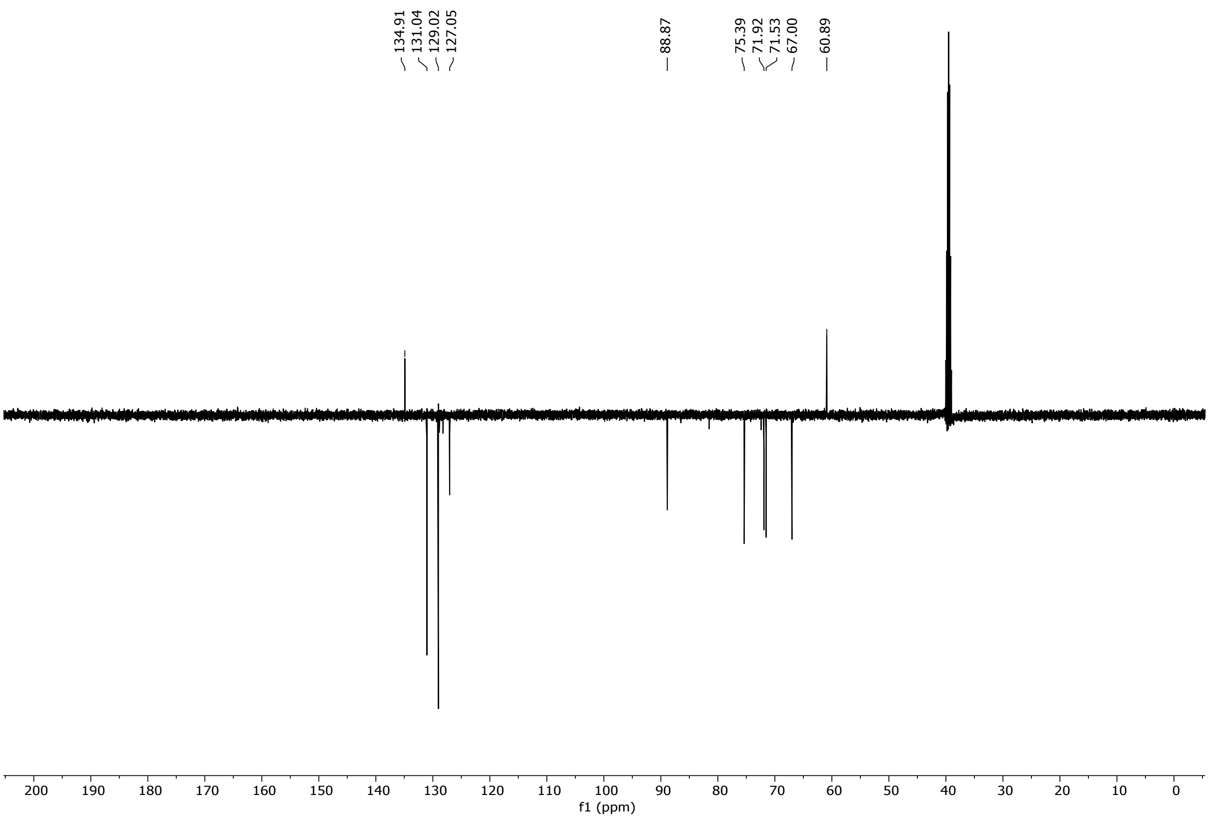

Figure S38: ^{1}H NMR spectrum (top) and ^{13}C NMR spectrum (bottom) of **5**.

Phenyl 4,6-*O*-benzylidene-1-thio-α-D-mannopyranoside (6)

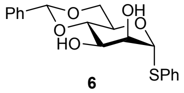

6

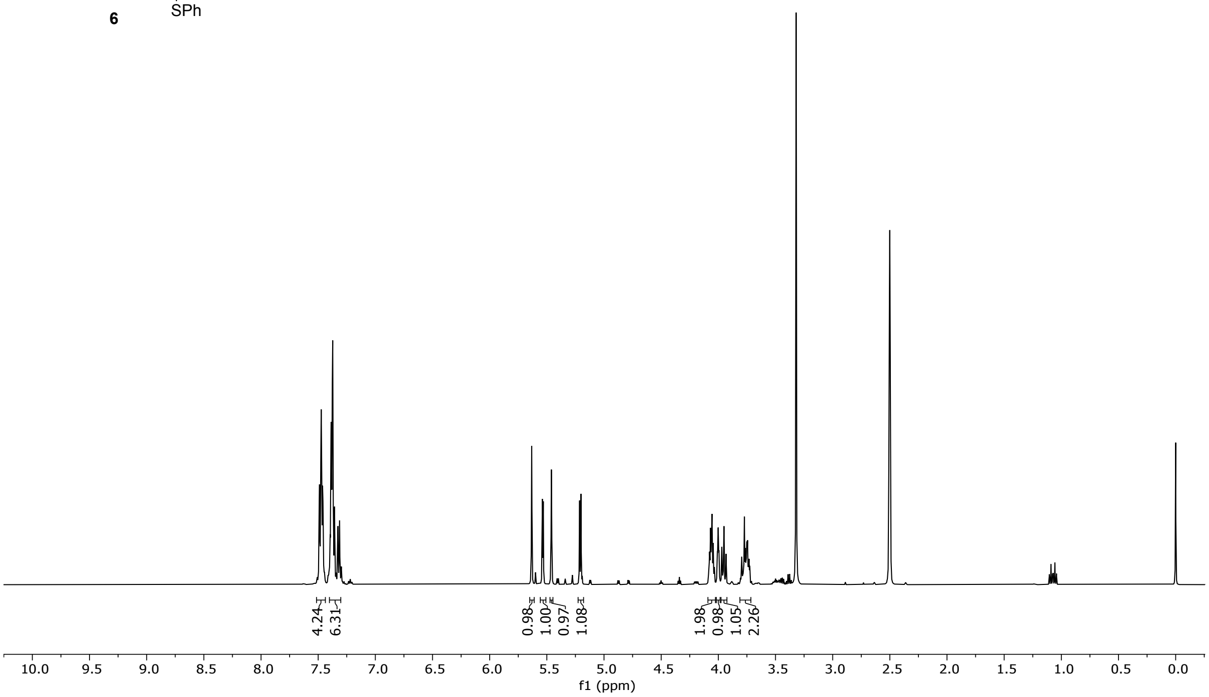

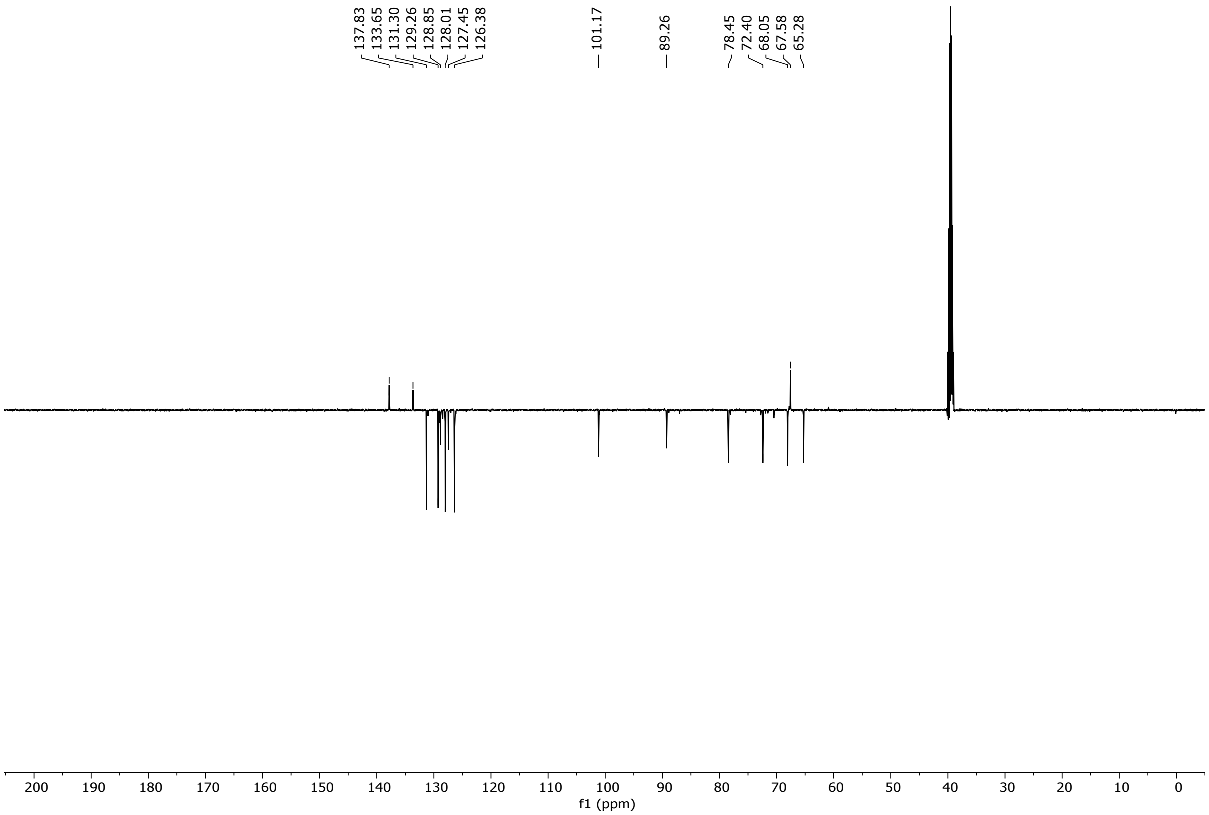

Figure S39: ^{1}H NMR spectrum (top) and ^{13}C NMR spectrum (bottom) of **6**.

Phenyl 4,6-*O*-benzylidene-3-*O*-(naphthalene-2-ylmethyl)-1-thio-α-D-mannopyranoside (7)

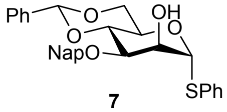

7

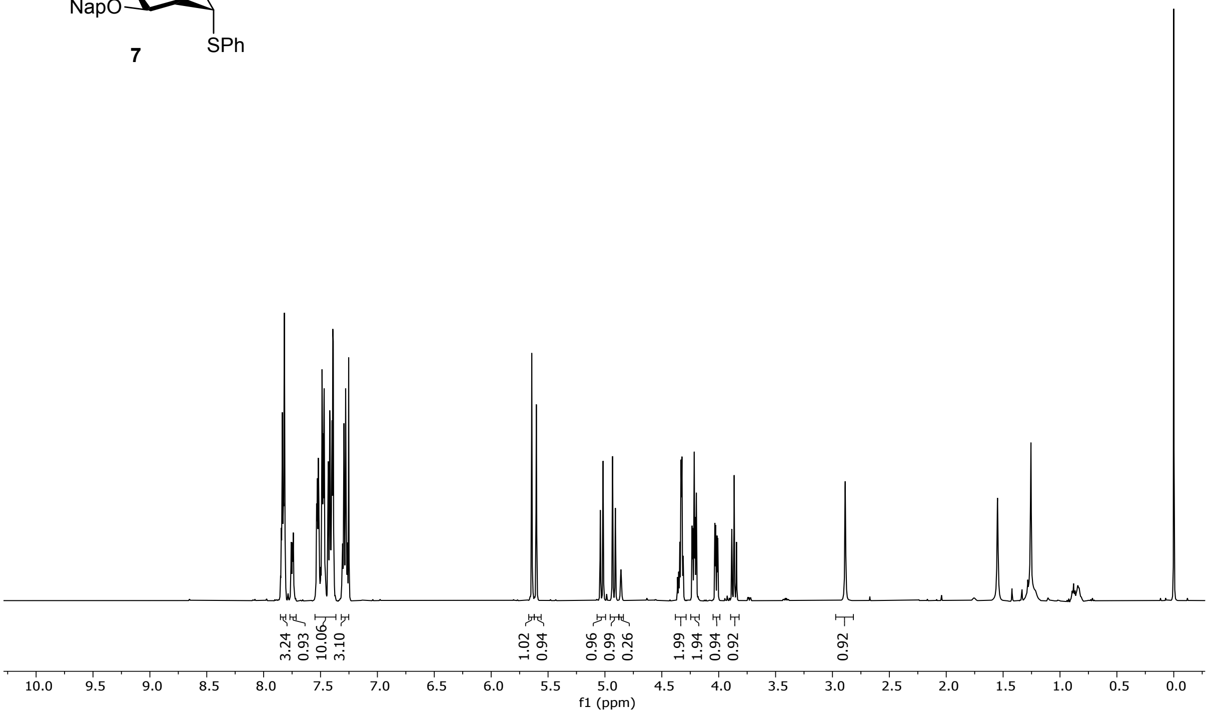

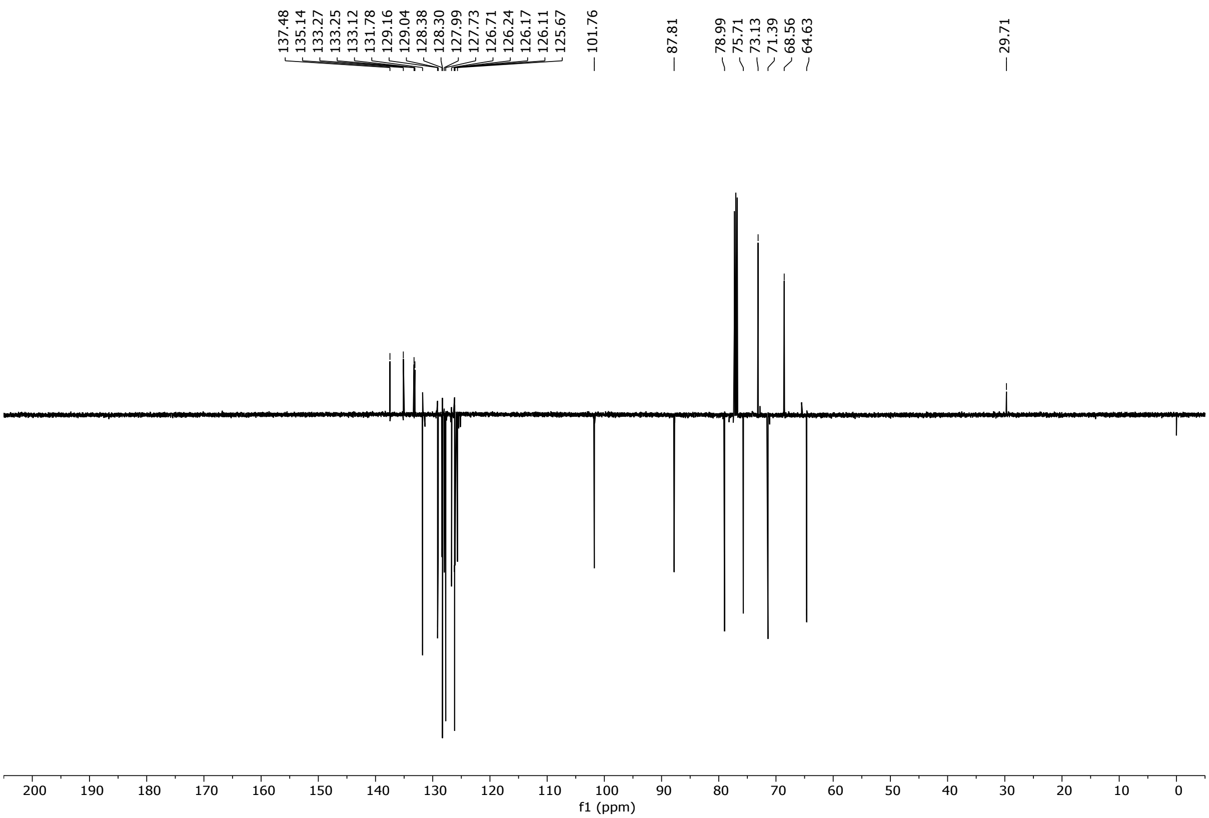

Figure S40: ^{1}H NMR spectrum (top) and ^{13}C NMR spectrum (bottom) of **7**.

Phenyl 2-*O*-Benzyl-4,6-*O*-benzylidene-3-*O*-(naphthalene-2-ylmethyl)-1-thio-α-D-mannopyranoside (8)

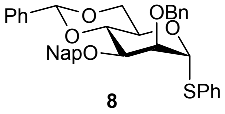

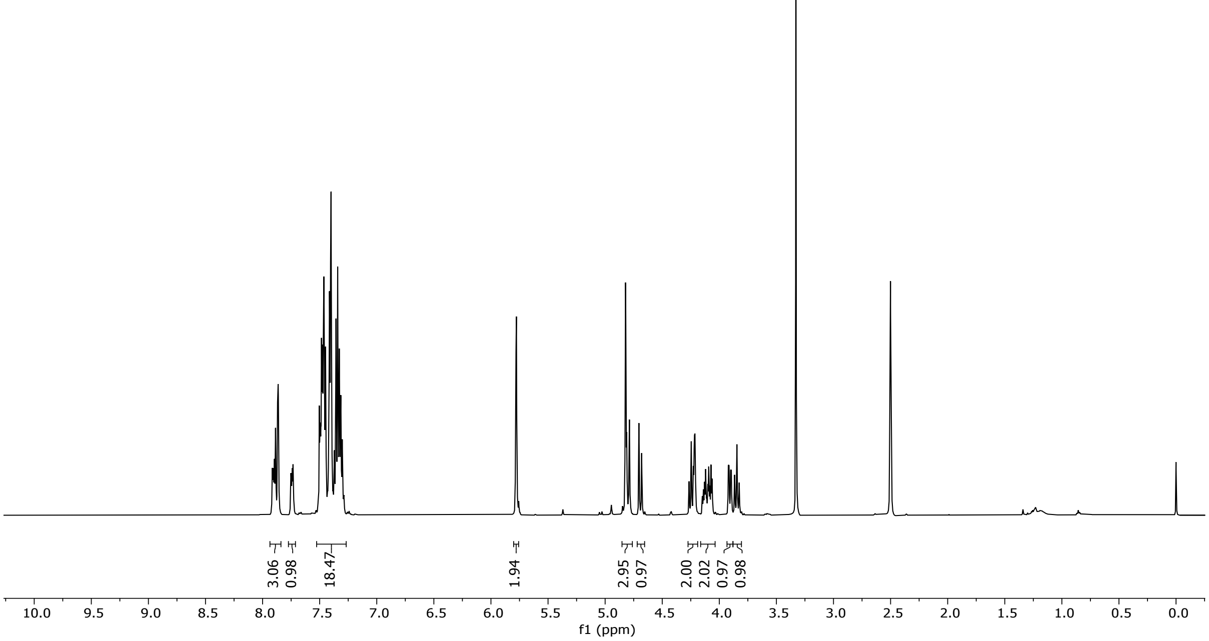

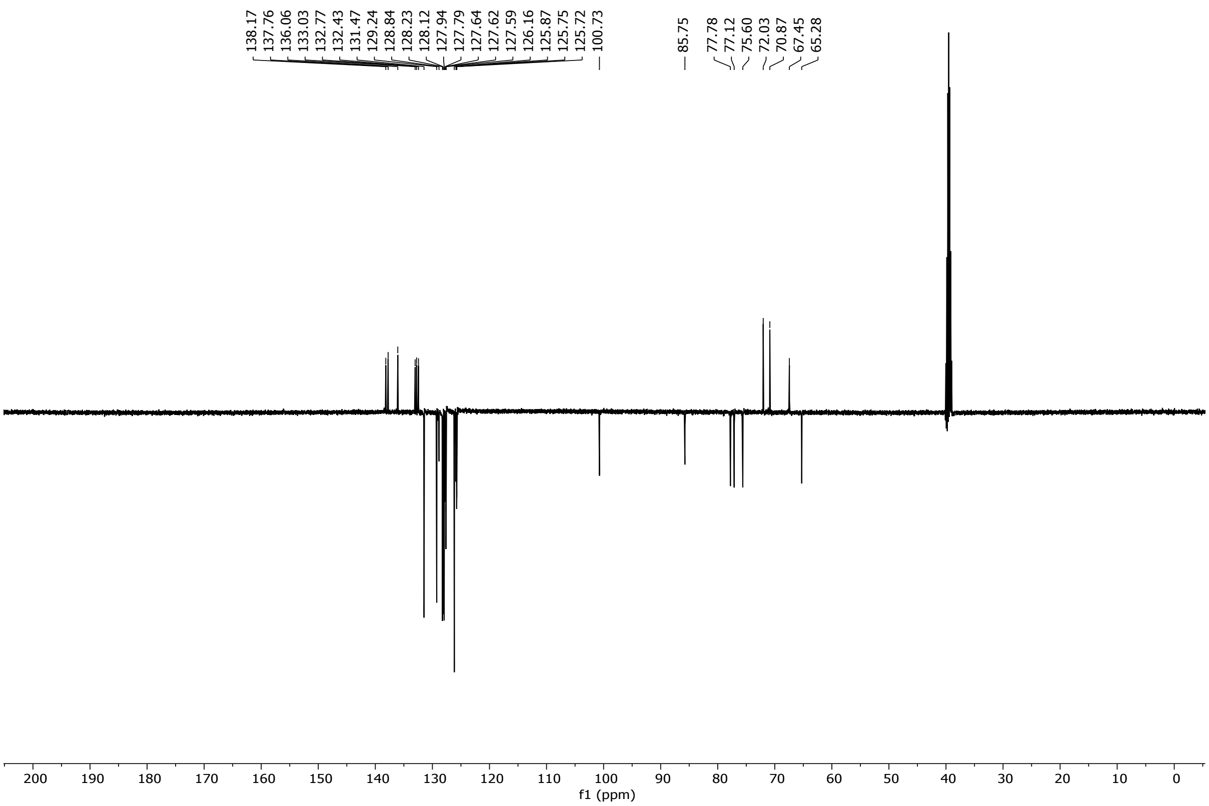

Figure S41: ^{1}H NMR spectrum (top) and ^{13}C NMR spectrum (bottom) of **8**.

Phenyl 2-*O*-benzyl-3-*O*-(naphthalene-2-ylmethyl)-1-thio-α-D-mannopyranoside (9)

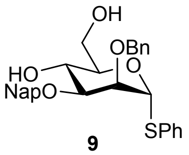

9

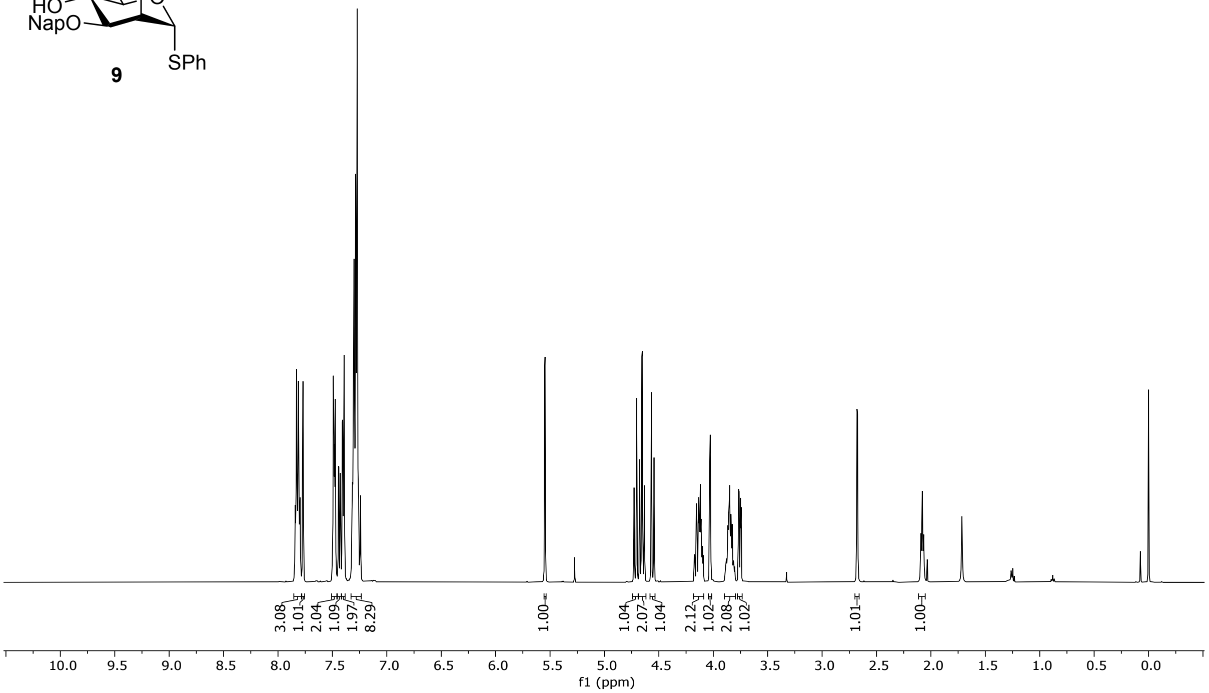

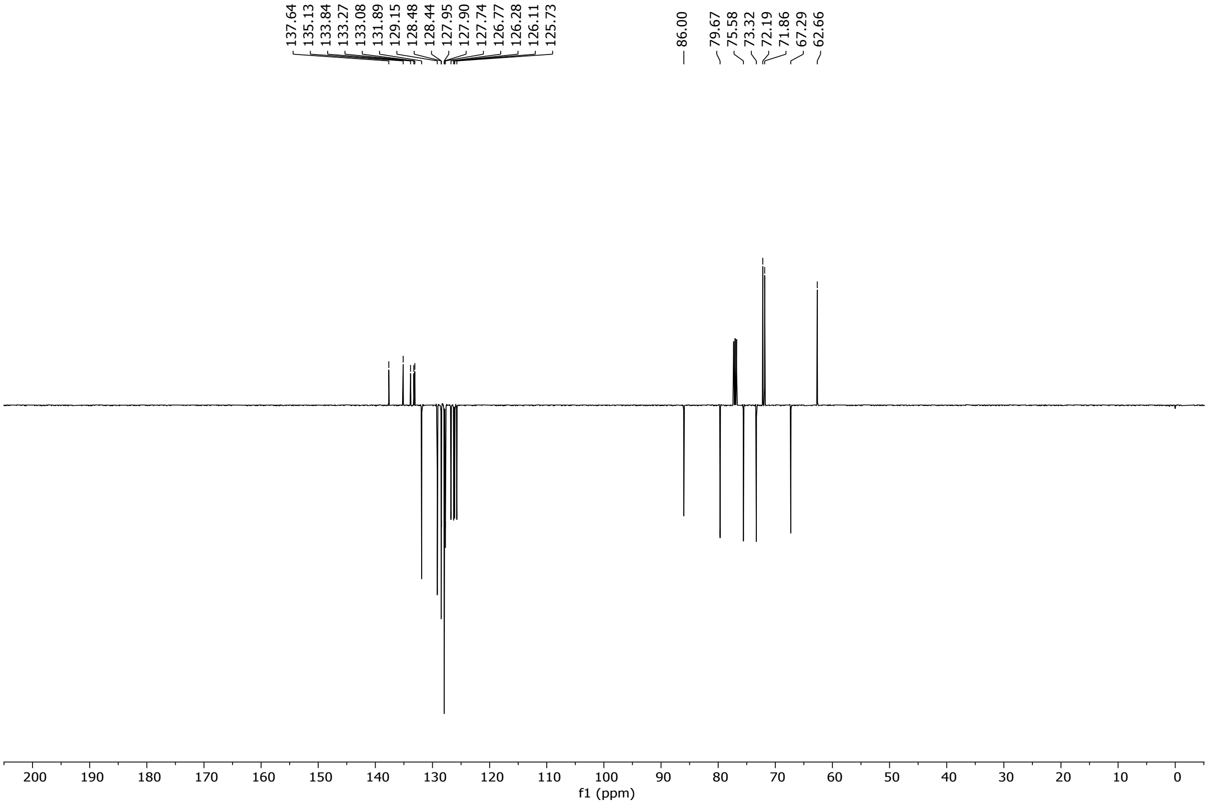

Figure S42: ^{1}H NMR spectrum (top) and ^{13}C NMR spectrum (bottom) of **9**.

Phenyl 2-*O*-benzyl-3-*O*-(naphthalene-2-ylmethyl)-1-thio-α-D-mannopyranosiduronic acid (10)

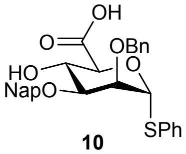

10

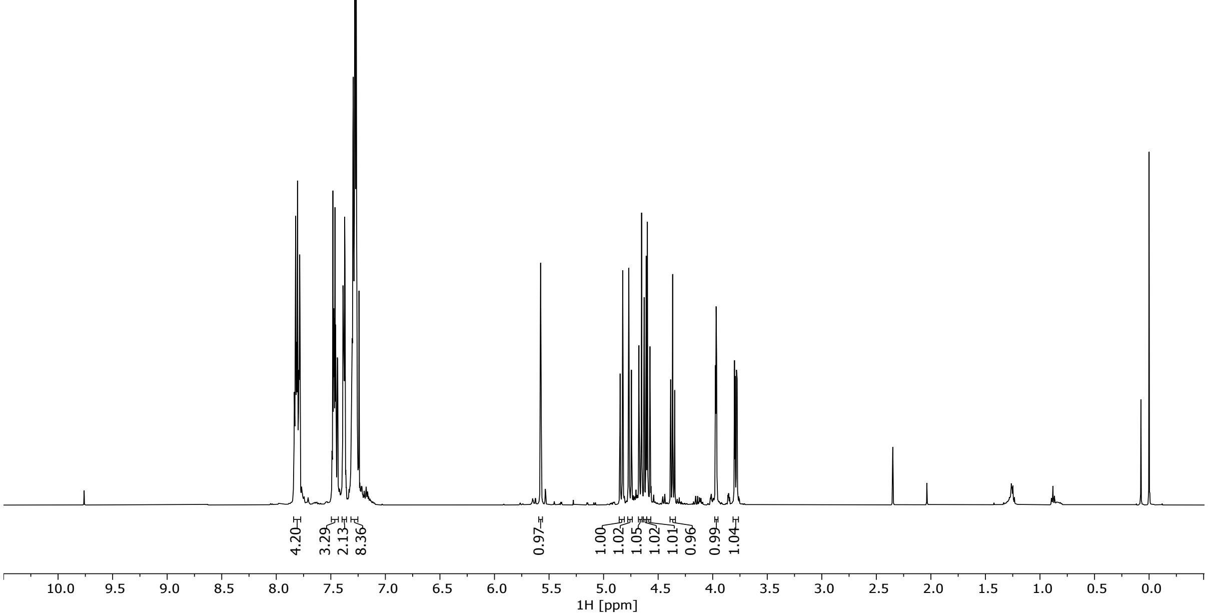

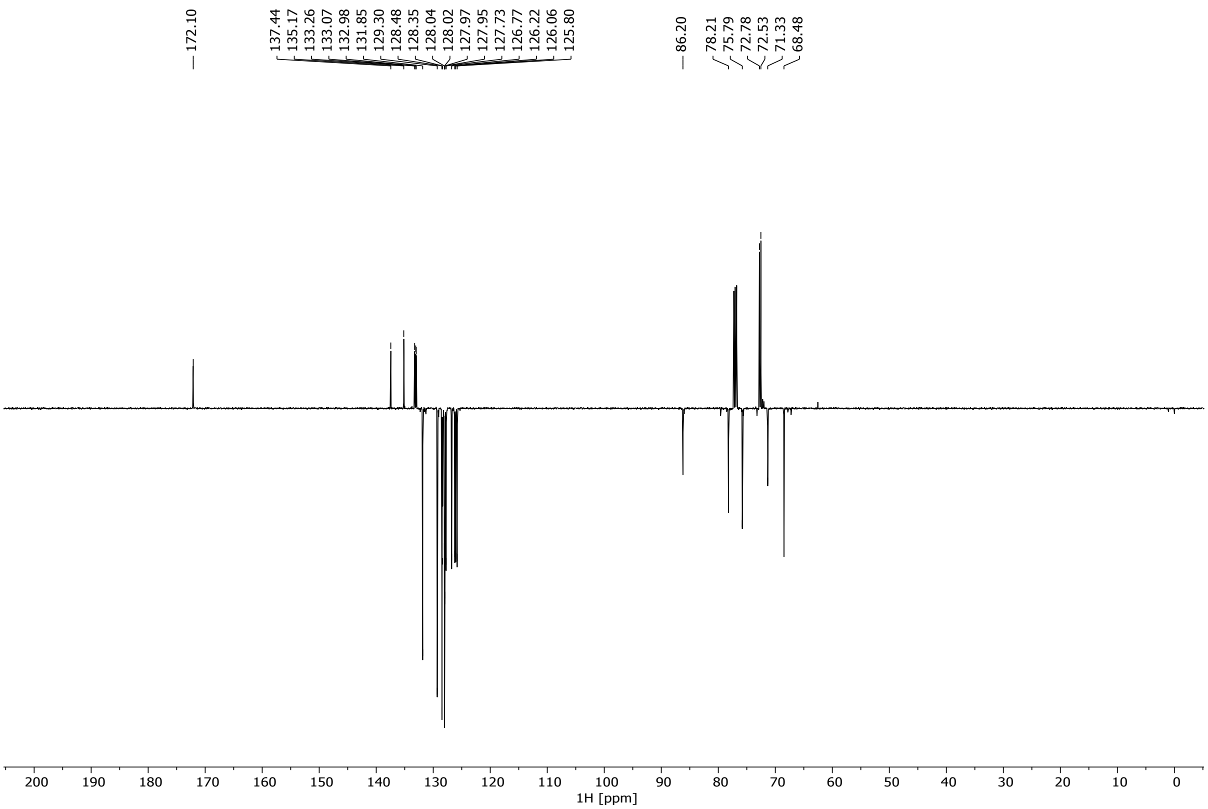

Figure S43: ^{1}H NMR spectrum (top) and ^{13}C NMR spectrum (bottom) of **10**.

Phenyl 2-*O*-benzyl-1-thio-α-D-mannopyranosiduronic acid (11)

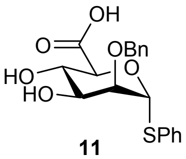

11

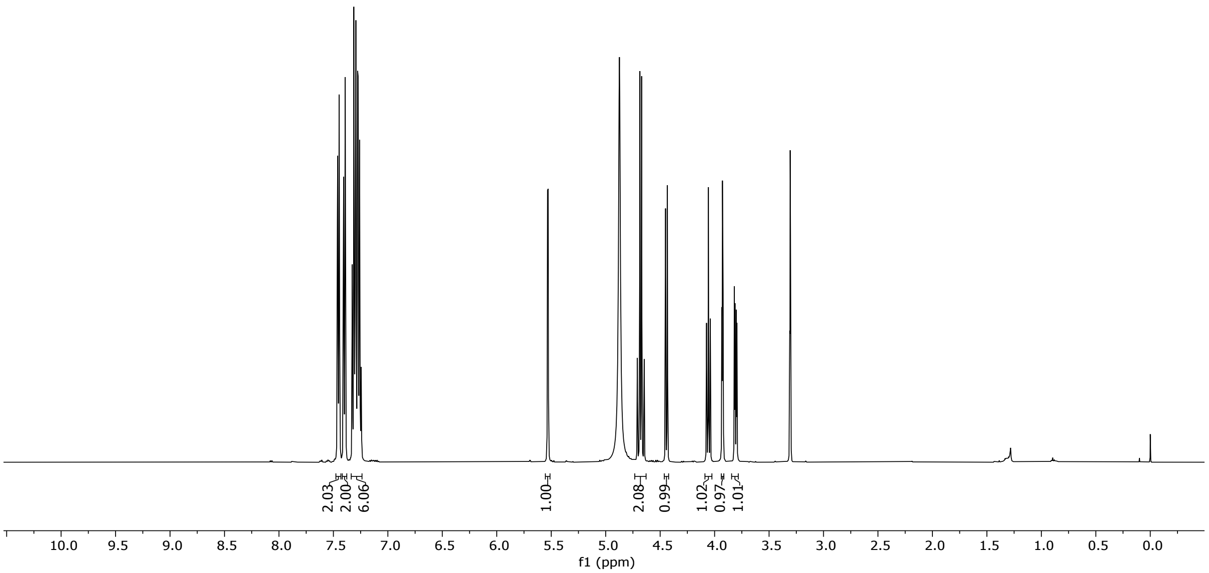

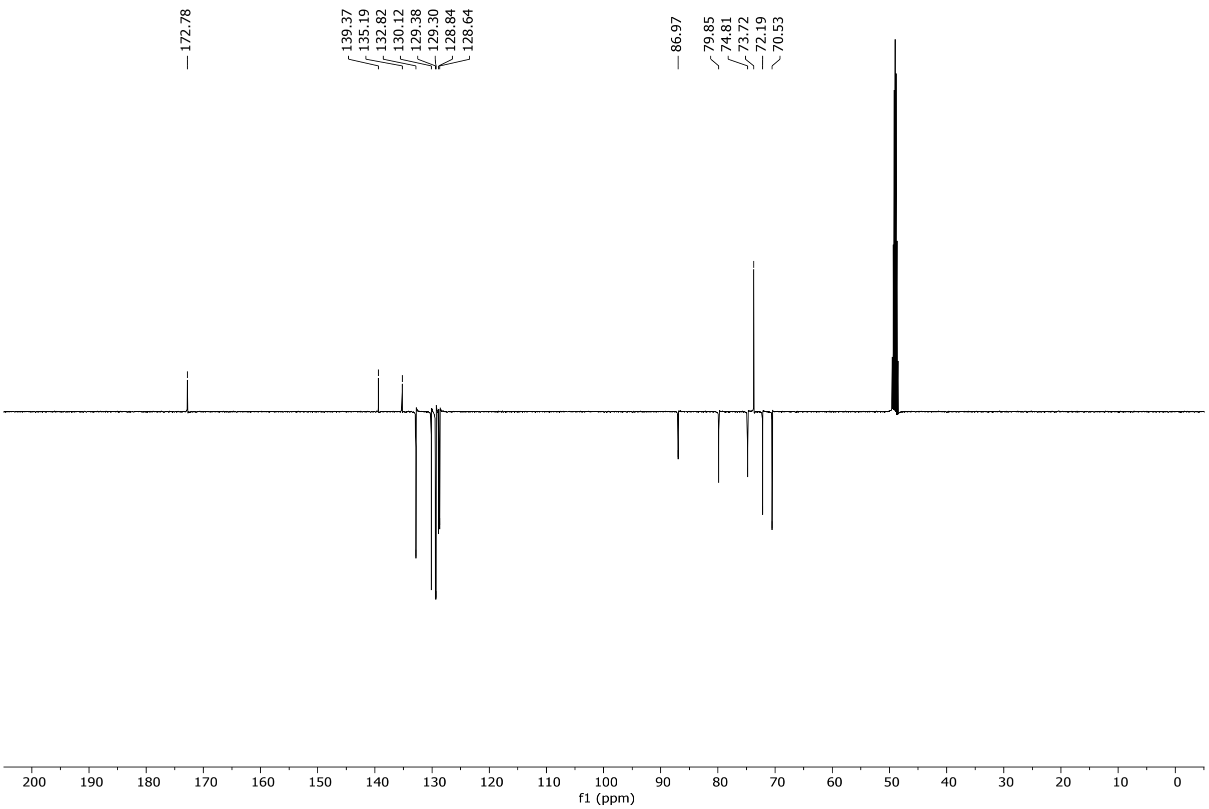

Figure S44: ^{1}H NMR spectrum (top) and ^{13}C NMR spectrum (bottom) of **11**.

4-*O*-acetyl-2-*O*-benzyl-1-thio-α-D-mannopyranosidurono-6,3-lactone (1)

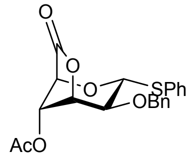

1

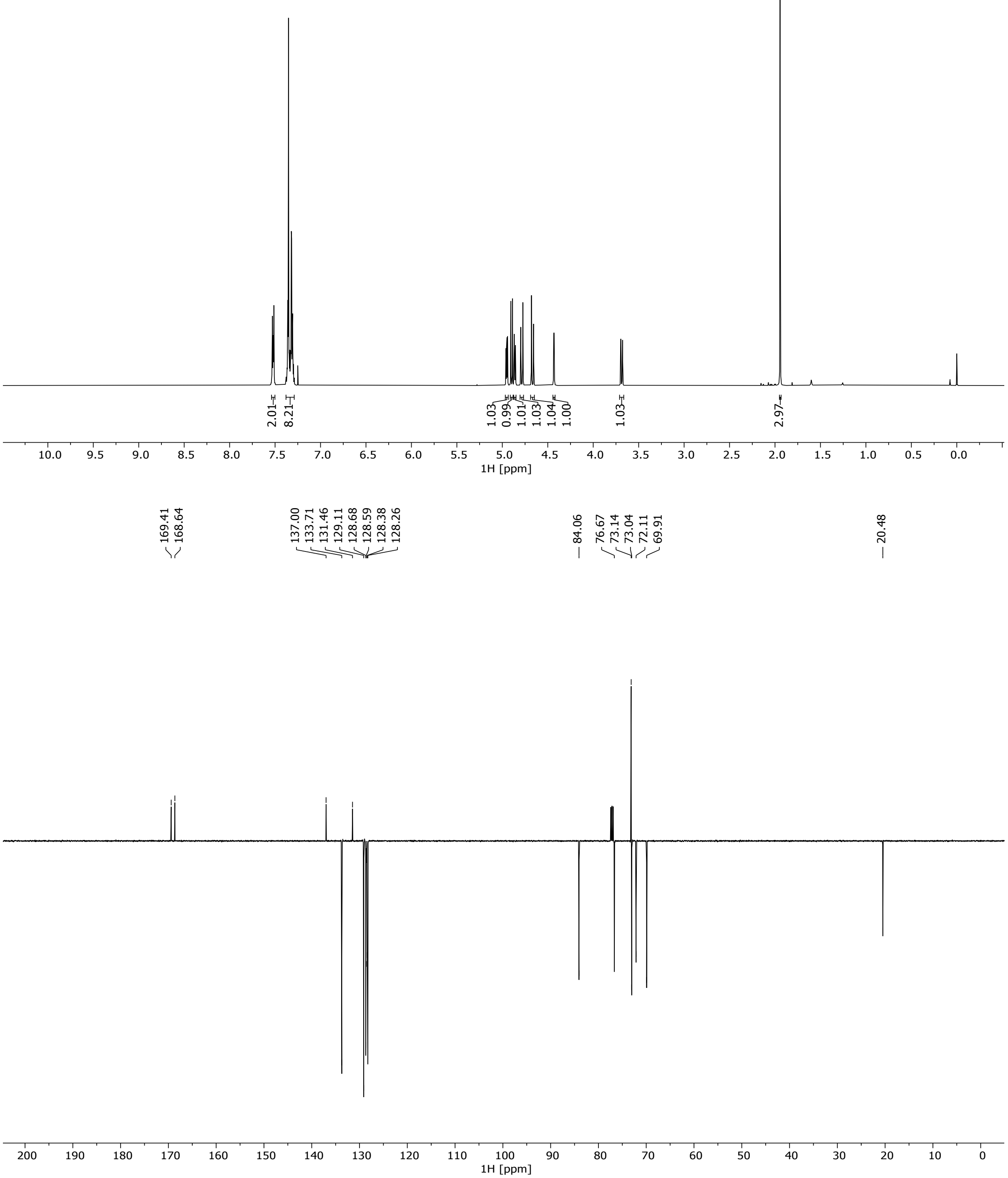

Figure S45: ^{1}H NMR spectrum (top) and ^{13}C NMR spectrum (bottom) of **1**.

Phenyl 2,3,4,6-tetra-*O*-acetate-1-thio-β-D-glucopyranoside (12)

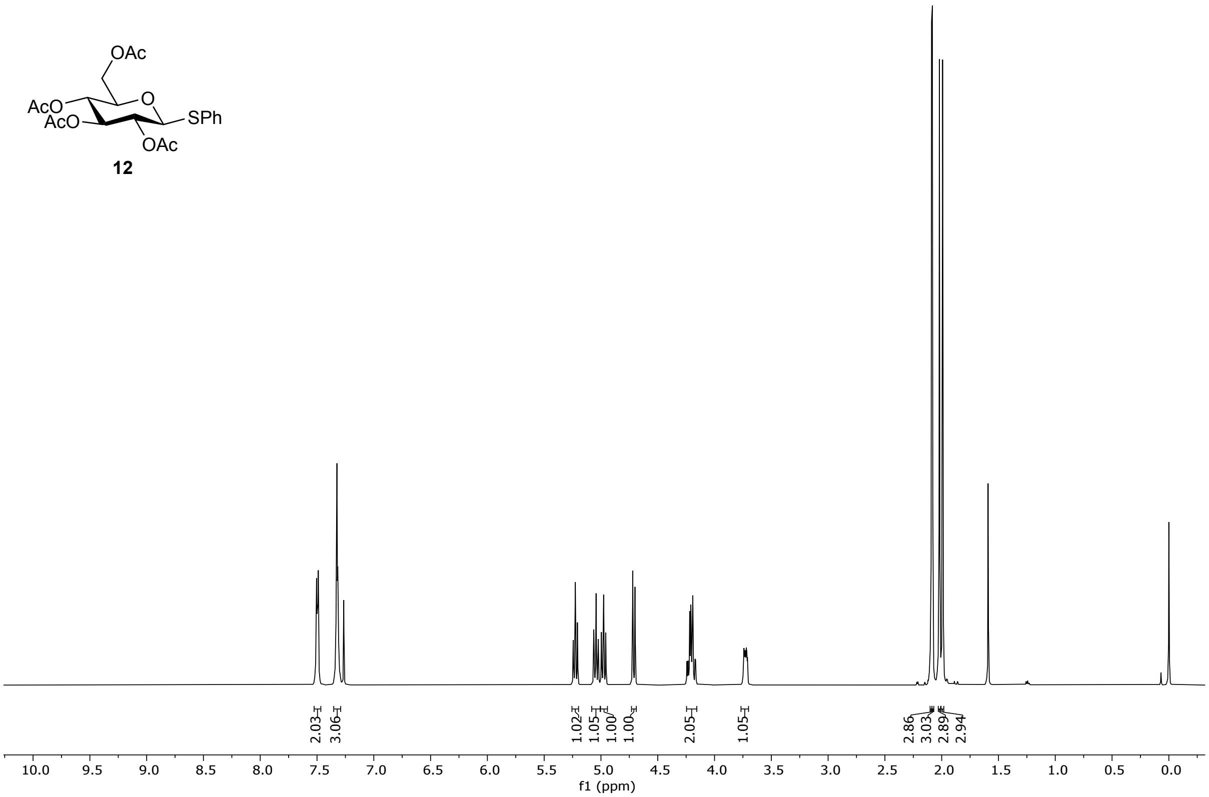

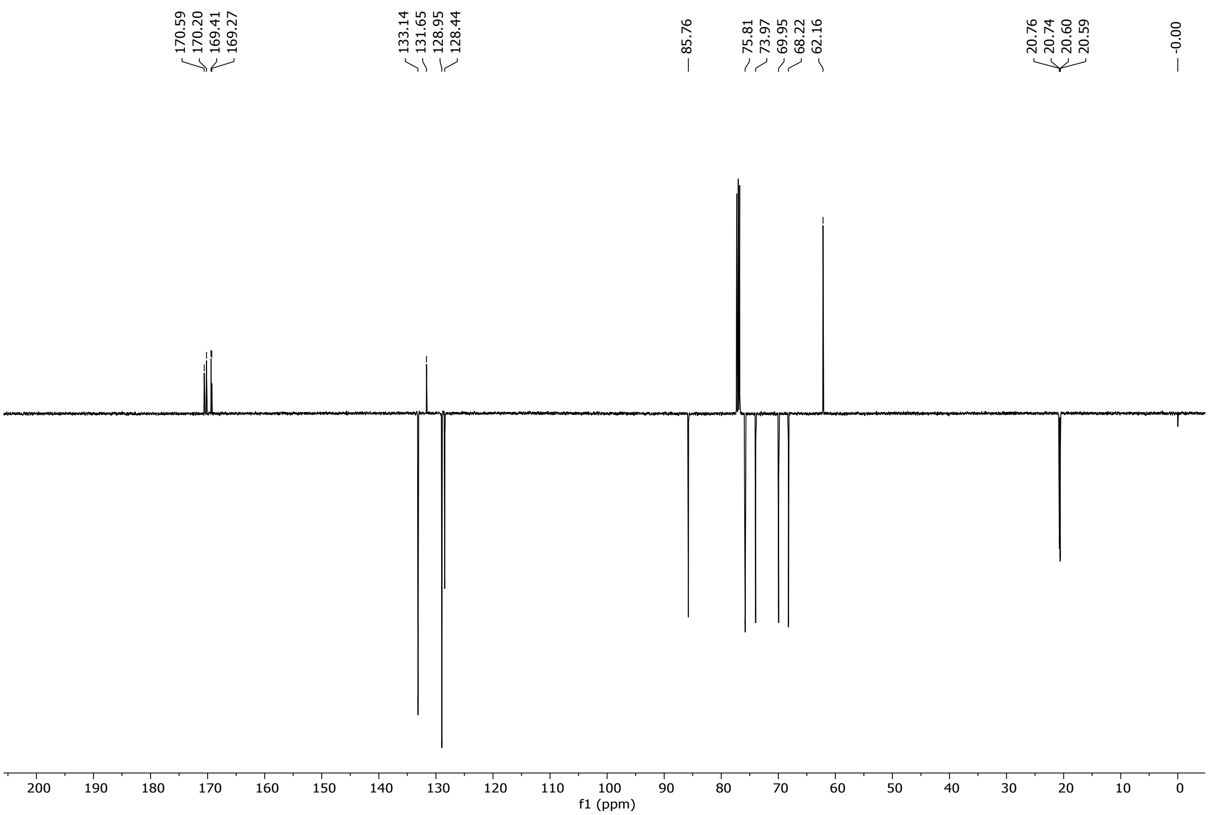

Figure S46: ^{1}H NMR spectrum (top) and ^{13}C NMR spectrum (bottom) of **12**.

Phenyl 1-thio-β-D-glucopyranoside (13)

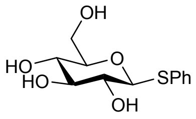

13

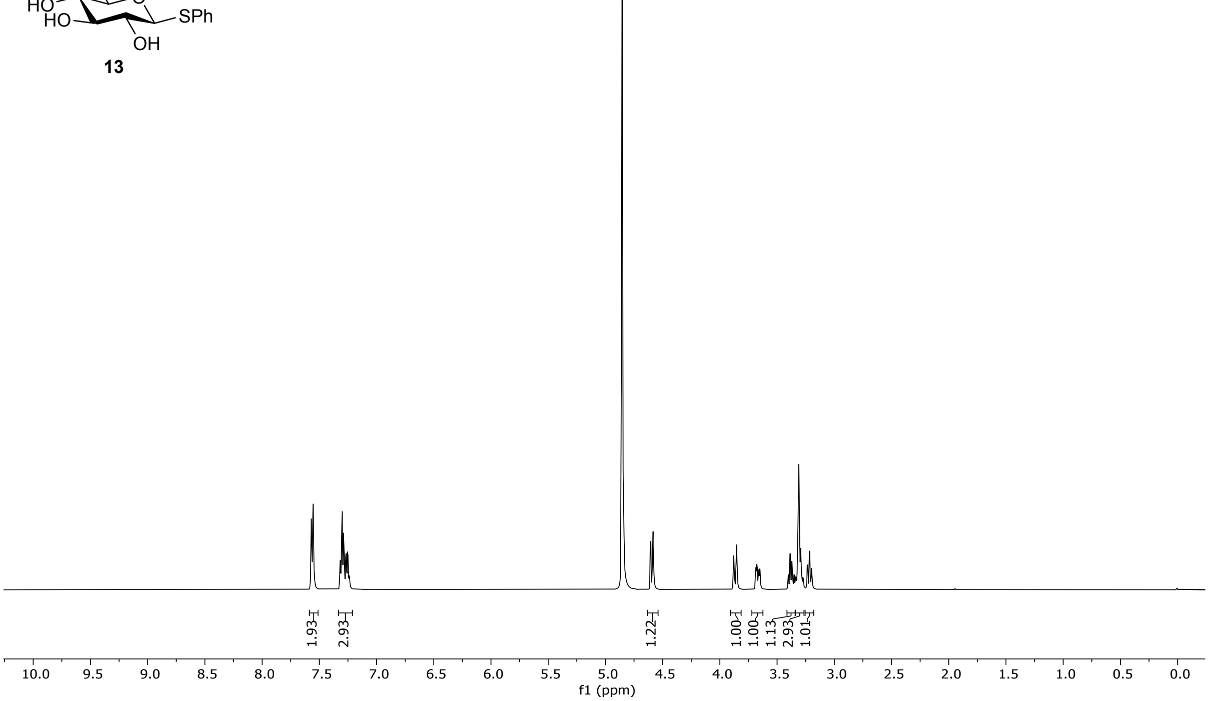

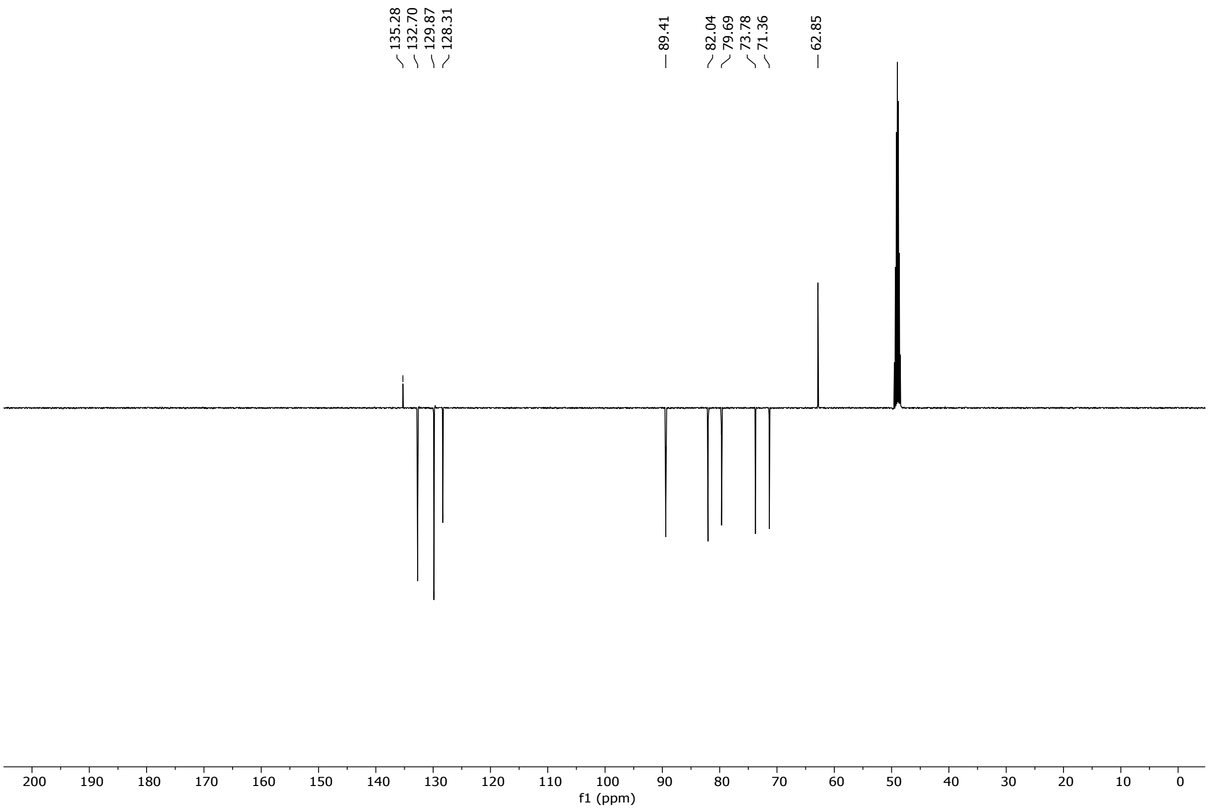

Figure S47: ^{1}H NMR spectrum (top) and ^{13}C NMR spectrum (bottom) of **13**.

Phenyl 4,6-*O*-benzylidene-1-thio-β-D-glucopyranoside (14)

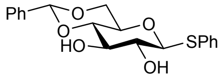

14

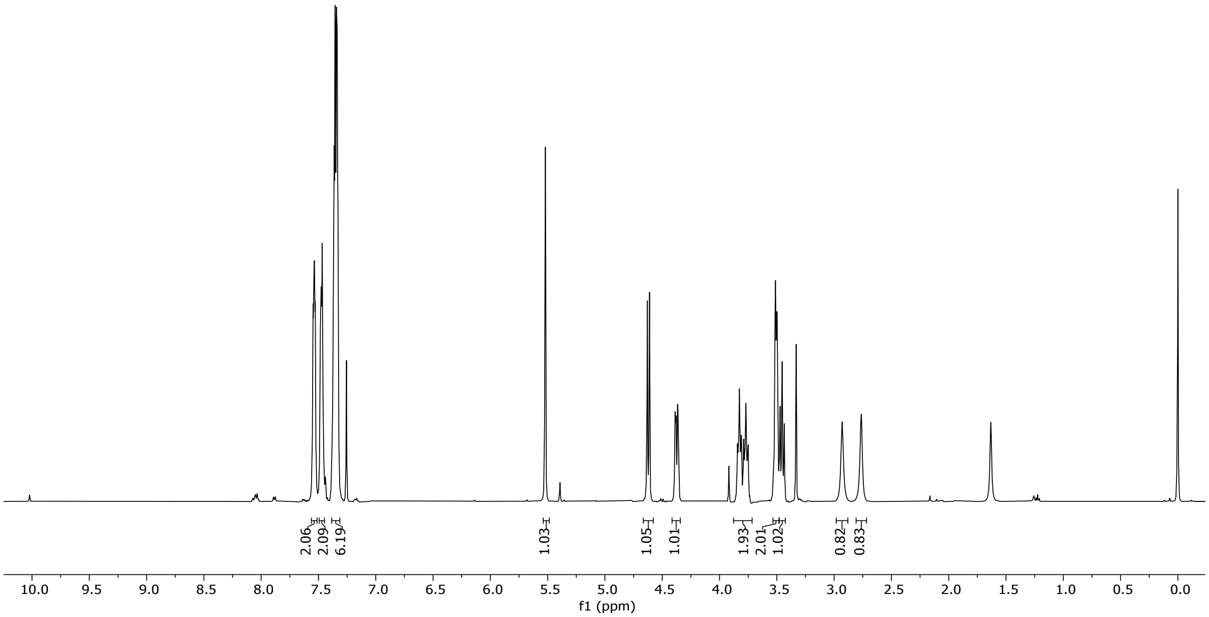

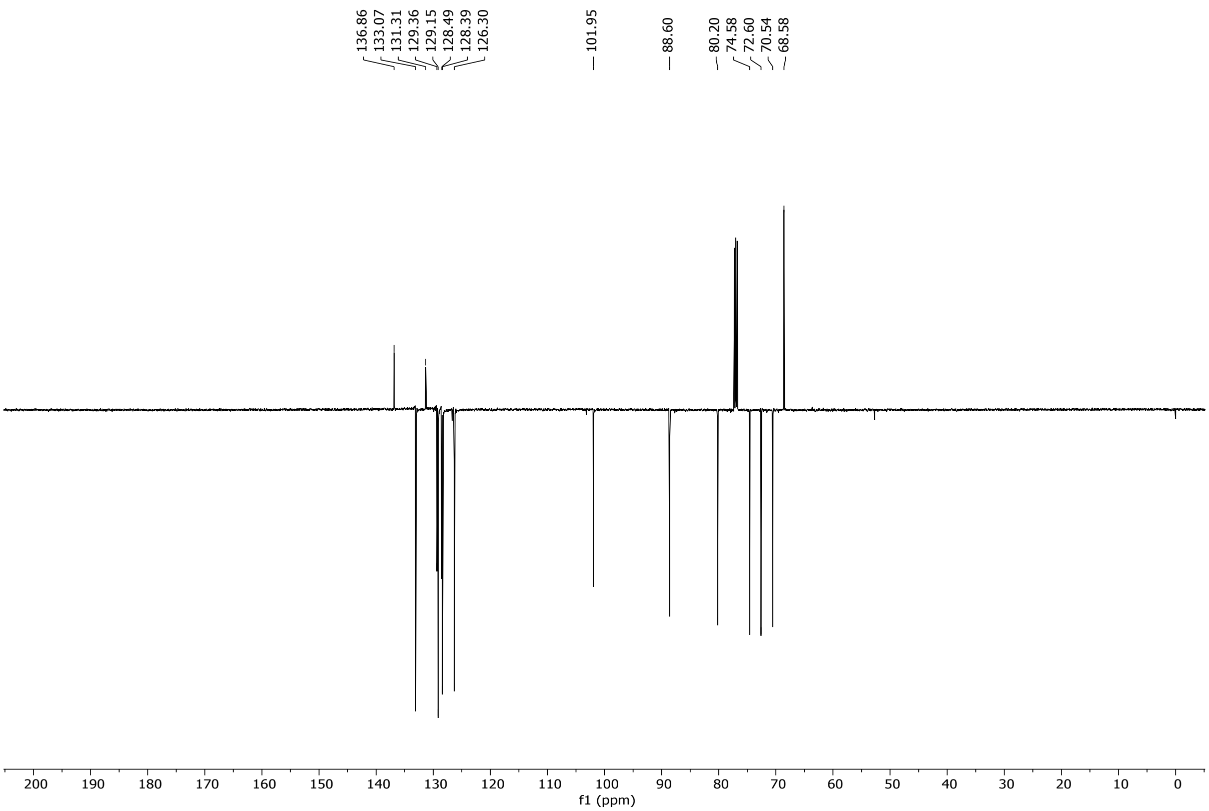

Figure S48: ^{1}H NMR spectrum (top) and ^{13}C NMR spectrum (bottom) of **14**.

Phenyl 3-*O*-(naphthalene-2-ylmethyl)-4,6-*O*-benzylidene-1-thio-β-D-glucopyranoside (15)

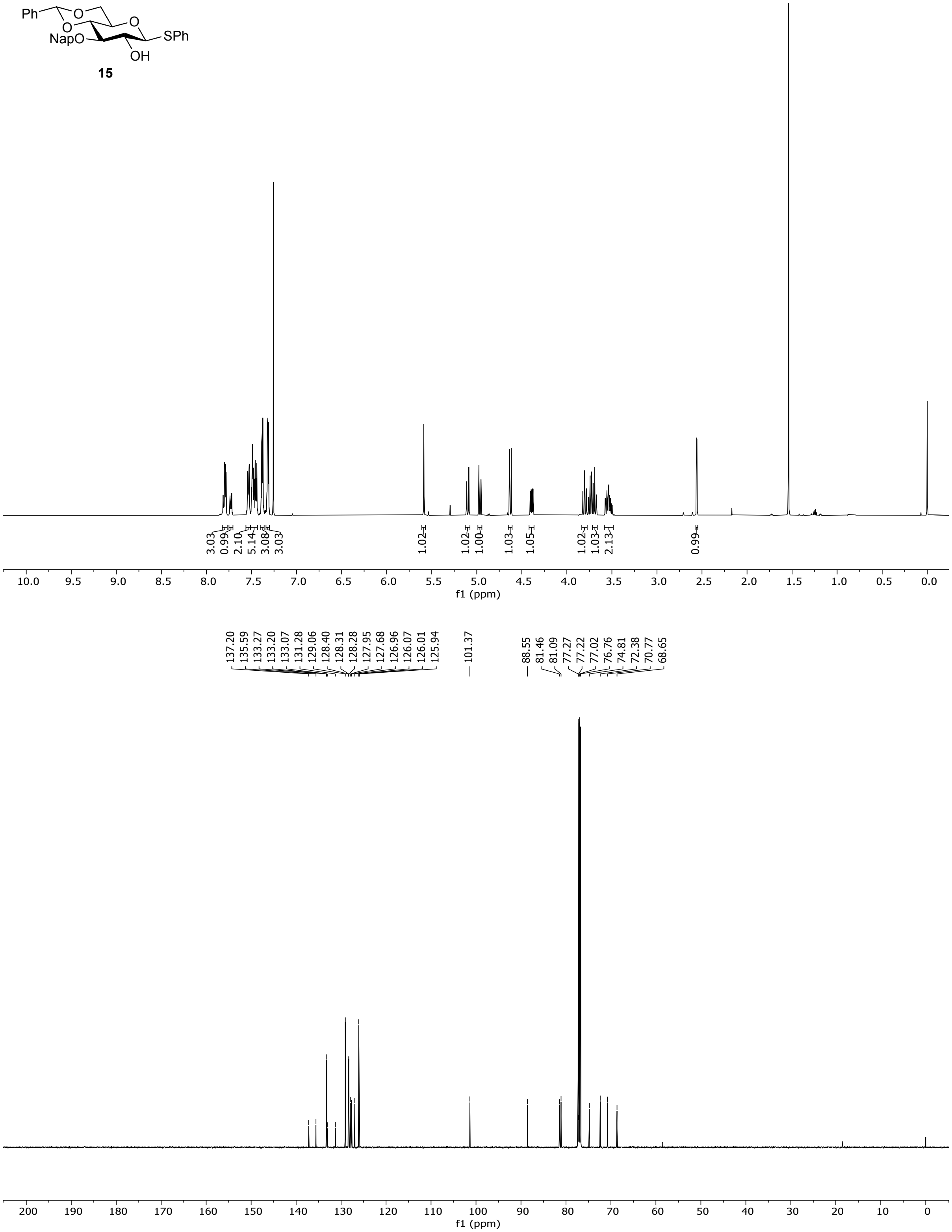

Figure S49: ^{1}H NMR spectrum (top) and ^{13}C NMR spectrum (bottom) of **15**.

Phenyl 2-*O*-benzyl-3-*O*-(naphthalene-2-ylmethyl)-4,6-*O*-benzylidene-1-thio-β-D-glucopyranoside (16)

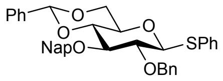

16

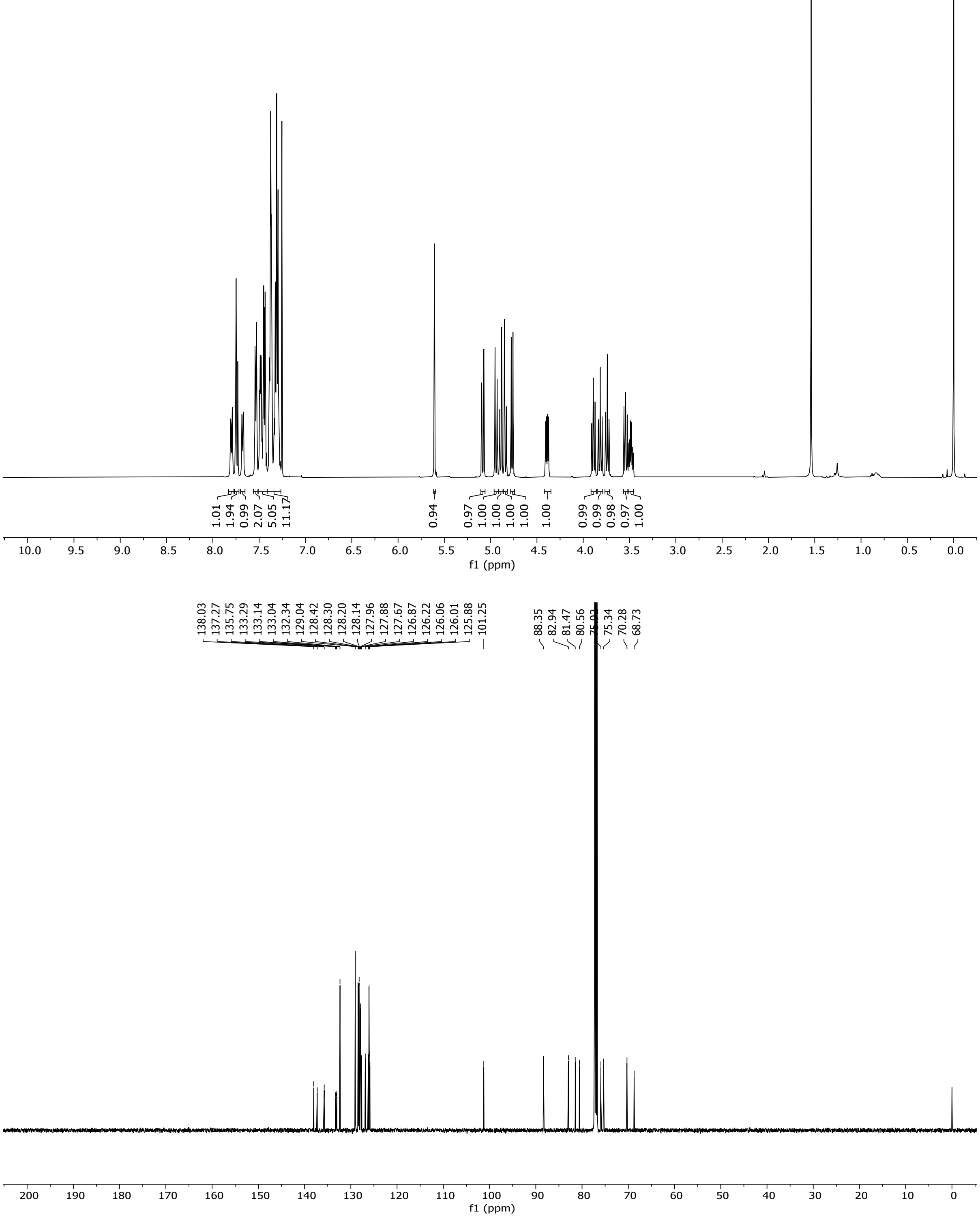

Figure S50: ^{1}H NMR spectrum (top) and ^{13}C NMR spectrum (bottom) of **16**.

Phenyl 2-*O*-benzyl-3-*O*-(naphthalene-2-ylmethyl)-1-thio-β-D-glucopyranoside (17)

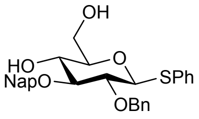

17

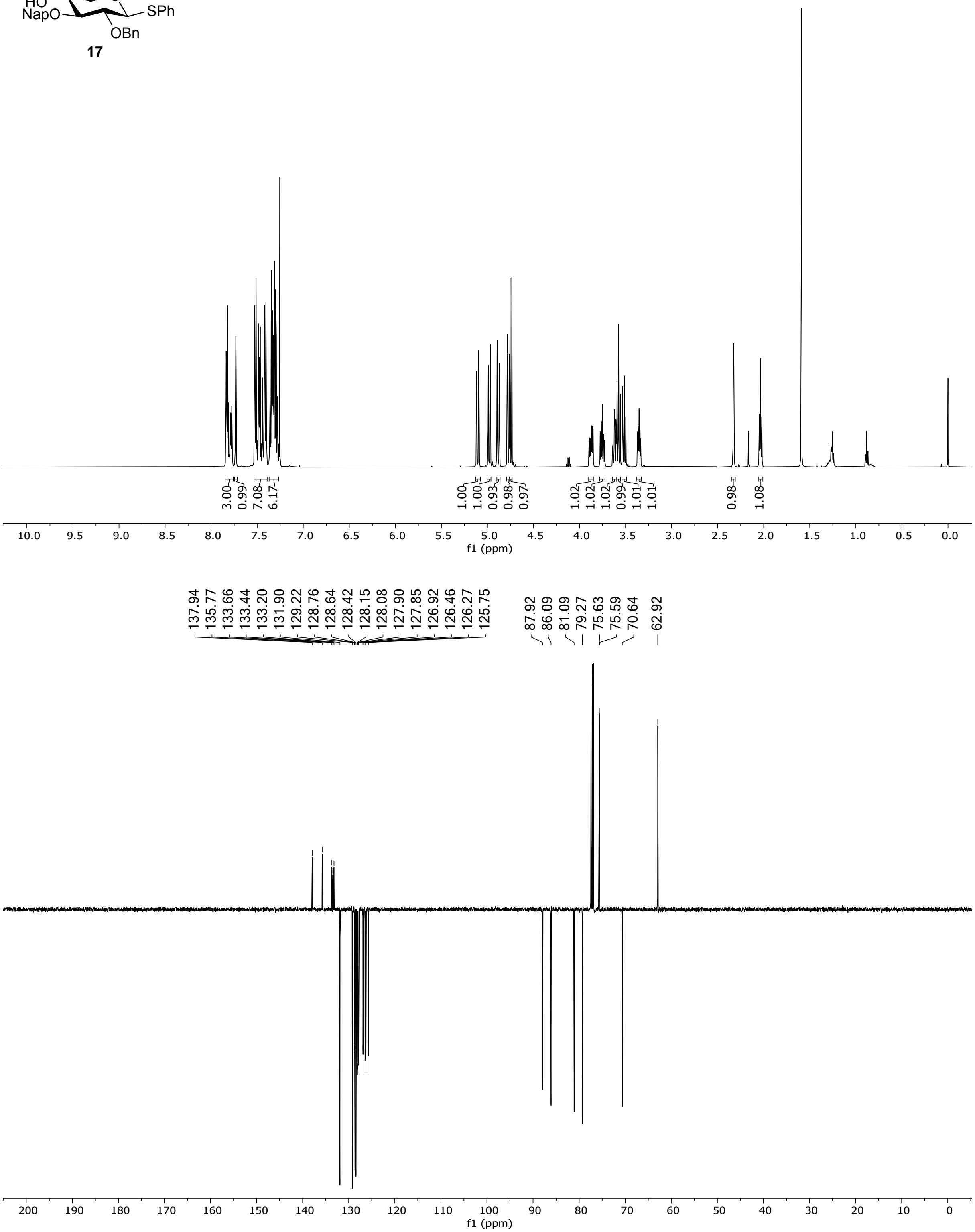

Figure S51: ^{1}H NMR spectrum (top) and ^{13}C NMR spectrum (bottom) of **17**.

Phenyl 2-*O*-benzyl-3-*O*-(naphthalene-2-ylmethyl)-1-thio-β-D-glucopyranosiduronic acid (18)

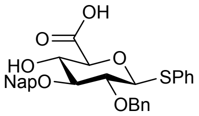

18

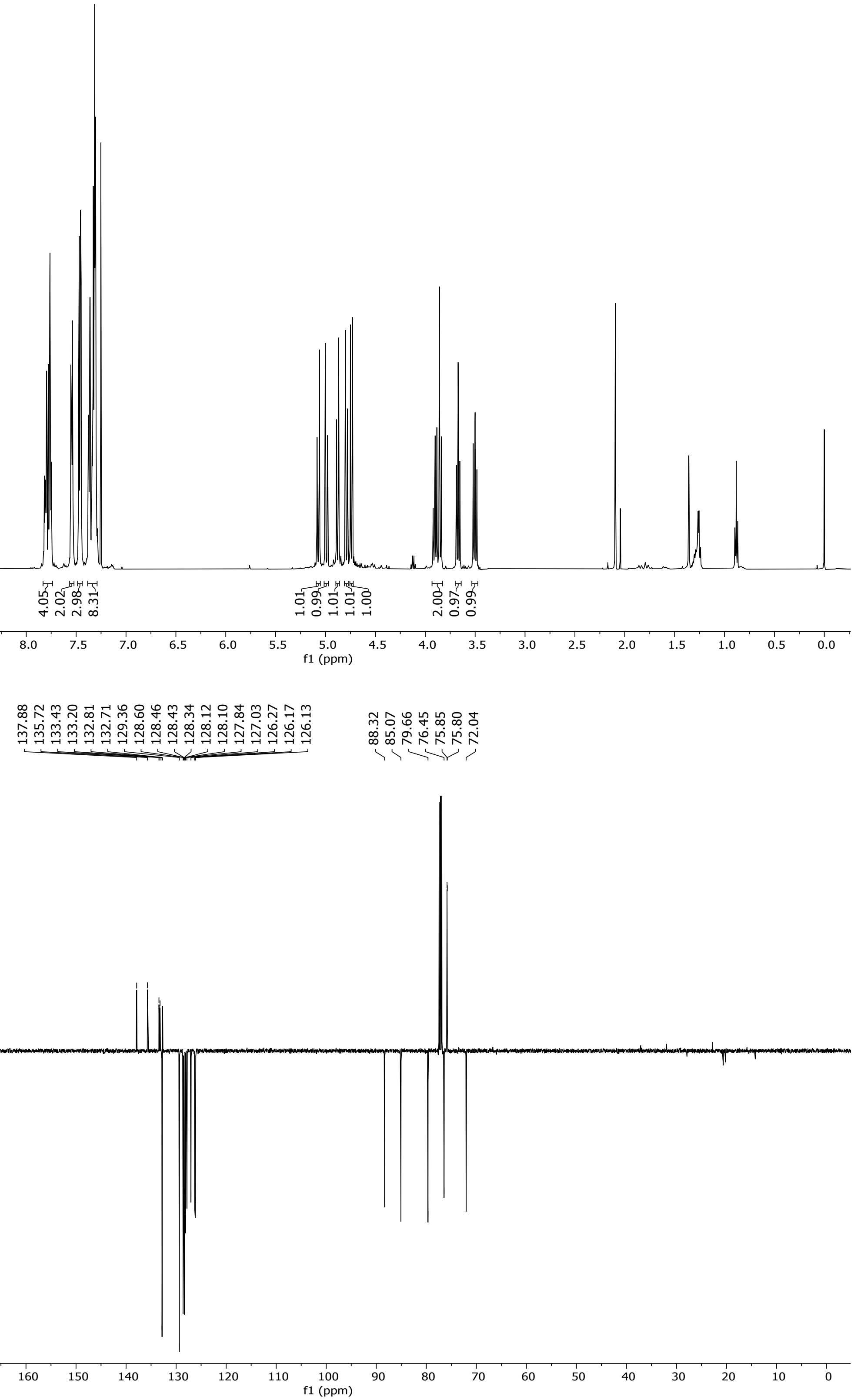

Figure S52: ^{1}H NMR spectrum (top) and ^{13}C NMR spectrum (bottom) of **18**.

Phenyl 2-*O*-benzyl-1-thio-β-D-glucopyranosiduronic acid (19)

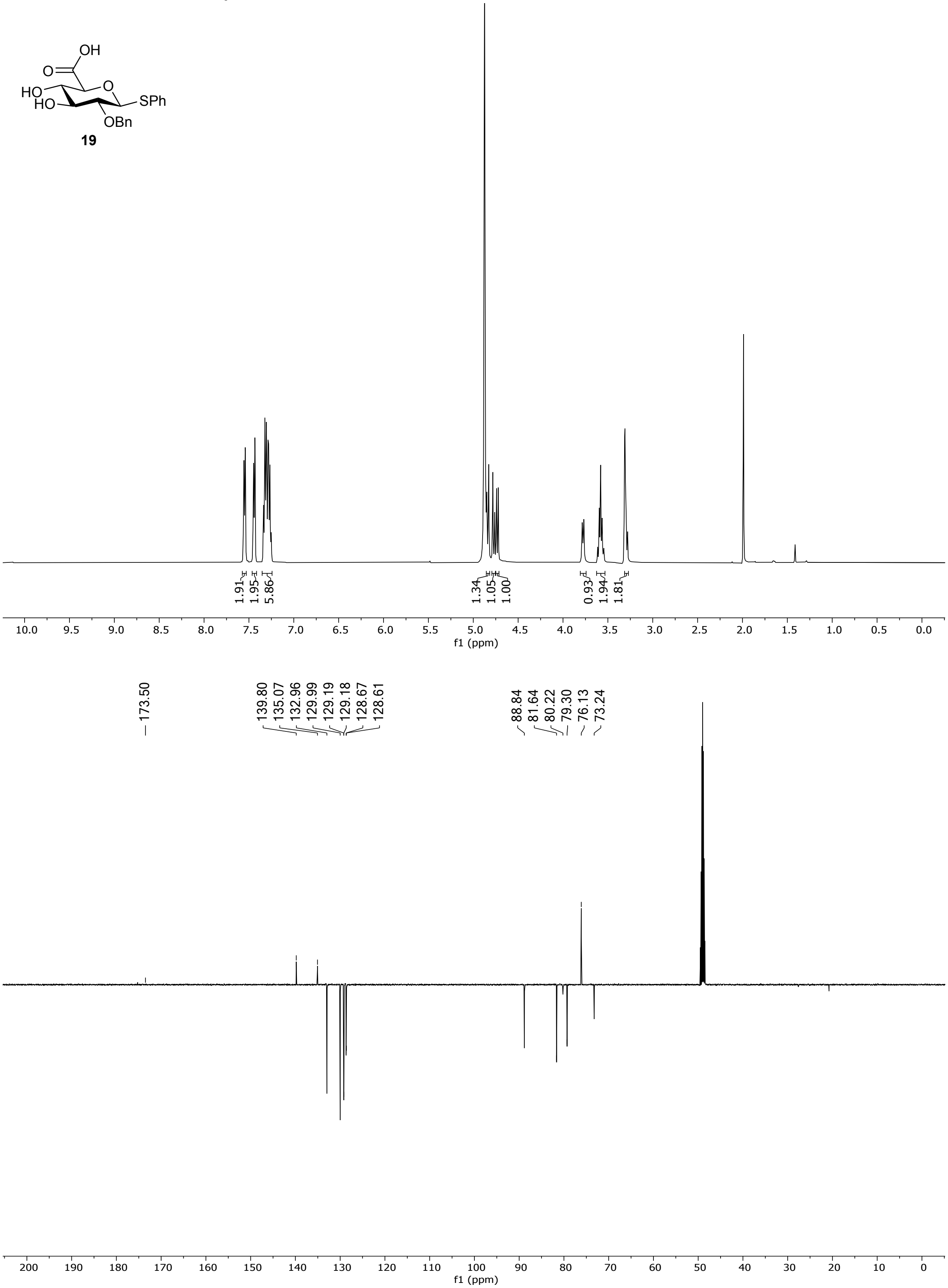

Figure S53: ^{1}H NMR spectrum (top) and ^{13}C NMR spectrum (bottom) of **19**.

4-*O*-Acetyl-2-*O*-benzyl-1-thio-α-D-glucopyranosidurono-6,3-lactone (2)

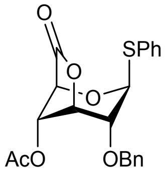

2

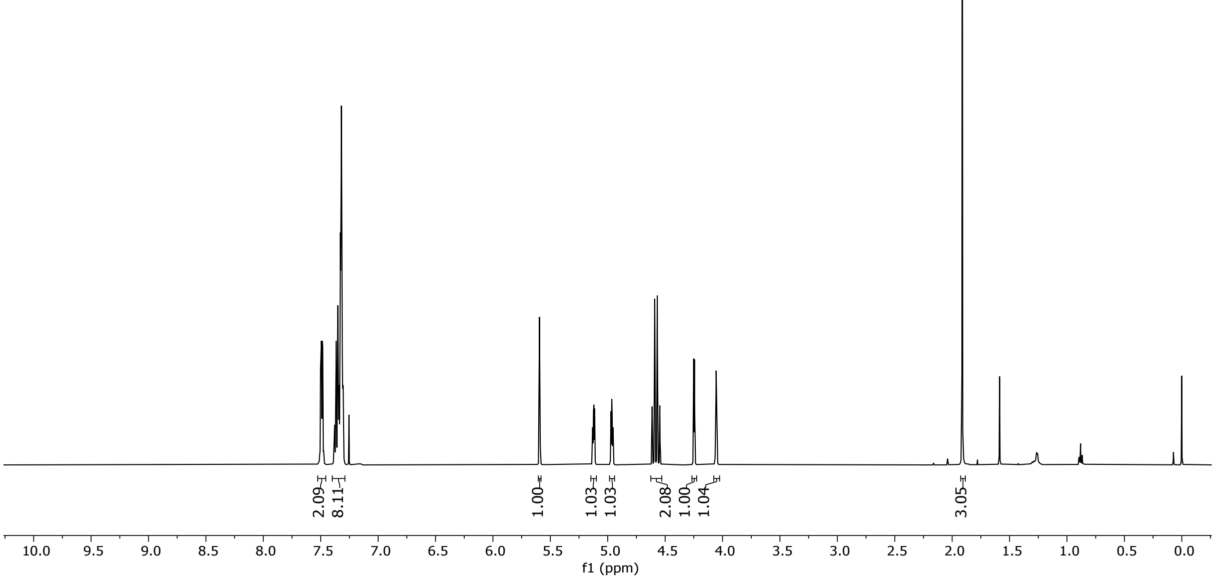

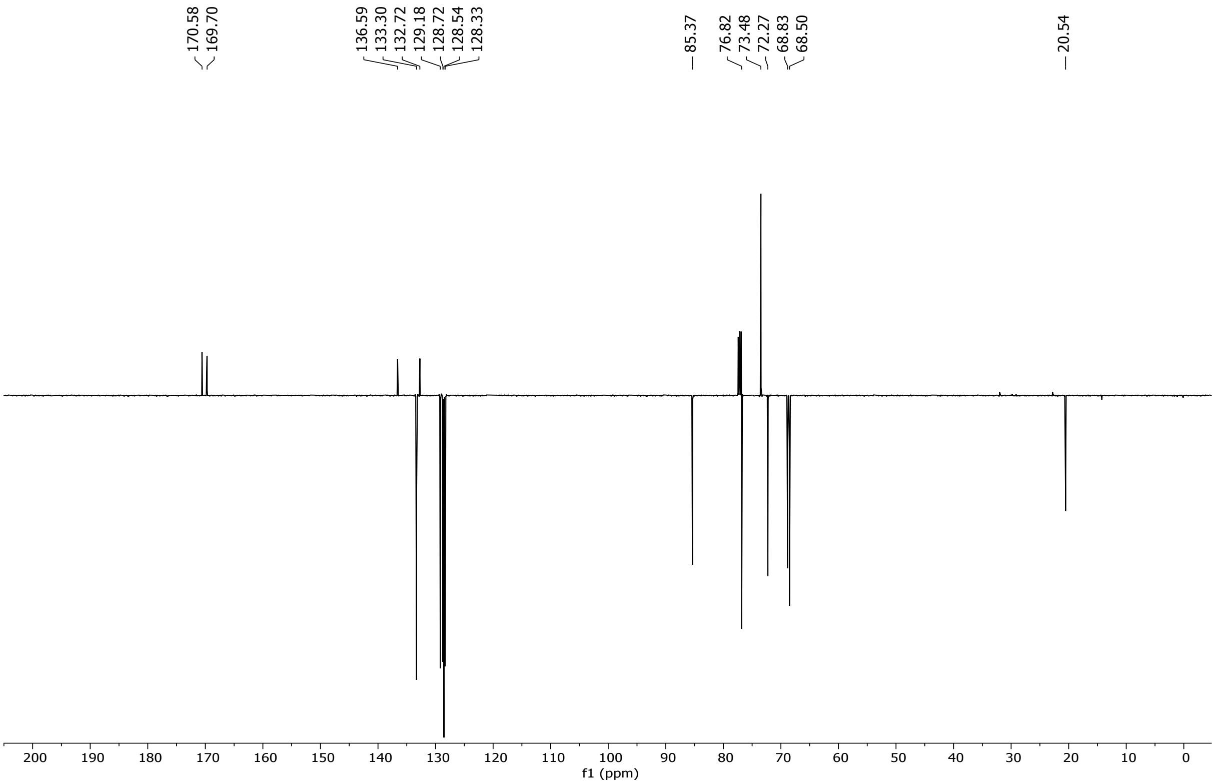

Figure S54: ^{1}H NMR spectrum (top) and ^{13}C NMR spectrum (bottom) of **2**.

References

1 Pracht, P., Bohle, F. & Grimme, S. Automated exploration of the low-energy chemical space with fast quantum chemical methods. *Physical Chemistry Chemical Physics* **22**, 7169-7192 (2020). https://doi.org/10.1039/c9cp06869d

2 Bannwarth, C., Ehlert, S. & Grimme, S. GFN2-xTB—An Accurate and Broadly Parametrized Self-Consistent Tight-Binding Quantum Chemical Method with Multipole Electrostatics and Density-Dependent Dispersion Contributions. *Journal of Chemical Theory and Computation* **15**, 1652-1671 (2019). https://doi.org/10.1021/acs.jctc.8b01176

3 Gaussian 16 Rev. C.02 (Wallingford, CT, 2016).

4 Neese, F. Software update: The ORCA program system—Version 5.0. *WIREs Computational Molecular Science* **12**, e1606 (2022). https://doi.org/https://doi.org/10.1002/wcms.1606

5 Elferink, H. *et al.* The glycosylation mechanisms of 6, 3-uronic acid lactones. *Angewandte Chemie International Edition* **58**, 8746-8751 (2019).

6 Wolfe, S., Pinto, B. M., Varma, V. & Leung, R. Y. N. The Perlin Effect: bond lengths, bond strengths, and the origins of stereoelectronic effects upon one-bond C–H coupling constants. *Canadian Journal of Chemistry* **68**, 1051-1062 (1990).

7 Juaristi, E. & Cuevas, G. Manifestations of stereoelectronic interactions in 1 JC–H one-bond coupling constants. *Accounts of Chemical Research* **40**, 961-970 (2007).
